# Supplementary material for: An Evolutionarily Conserved Function of Polycomb Silences the MHC Class I Antigen Presentation Pathway and Enables Immune Evasion in Cancer
Source: Cancer Cell. 2019 Oct 14;36(4):385–401.e8. doi: 10.1016/j.ccell.2019.08.008 (PMC6876280; doi:10.1016/j.ccell.2019.08.008)
Supplement: Document S1. Figures S1–S8 [file mmc1.pdf]

Cancer Cell, Volume *36*

Supplemental Information

An Evolutionarily Conserved Function of Polycomb Silences the MHC Class I Antigen Presentation Pathway and Enables Immune Evasion in Cancer

Marian L. Burr, Christina E. Sparbier, Kah Lok Chan, Yih-Chih Chan, Ariena Kersbergen, Enid Y.N. Lam, Elizabeth Azidis-Yates, Dane Vassiliadis, Charles C. Bell, Omer Gilan, Susan Jackson, Lavinia Tan, Stephen Q. Wong, Sebastian Hollizeck, Ewa M. Michalak, Hannah V. Siddle, Michael T. McCabe, Rab K. Prinjha, Glen R. Guerra, Benjamin J. Solomon, Shahneen Sandhu, Sarah-Jane Dawson, Paul A. Beavis, Richard W. Tothill, Carleen Cullinane, Paul J. Lehner, Kate D. Sutherland, and Mark A. Dawson

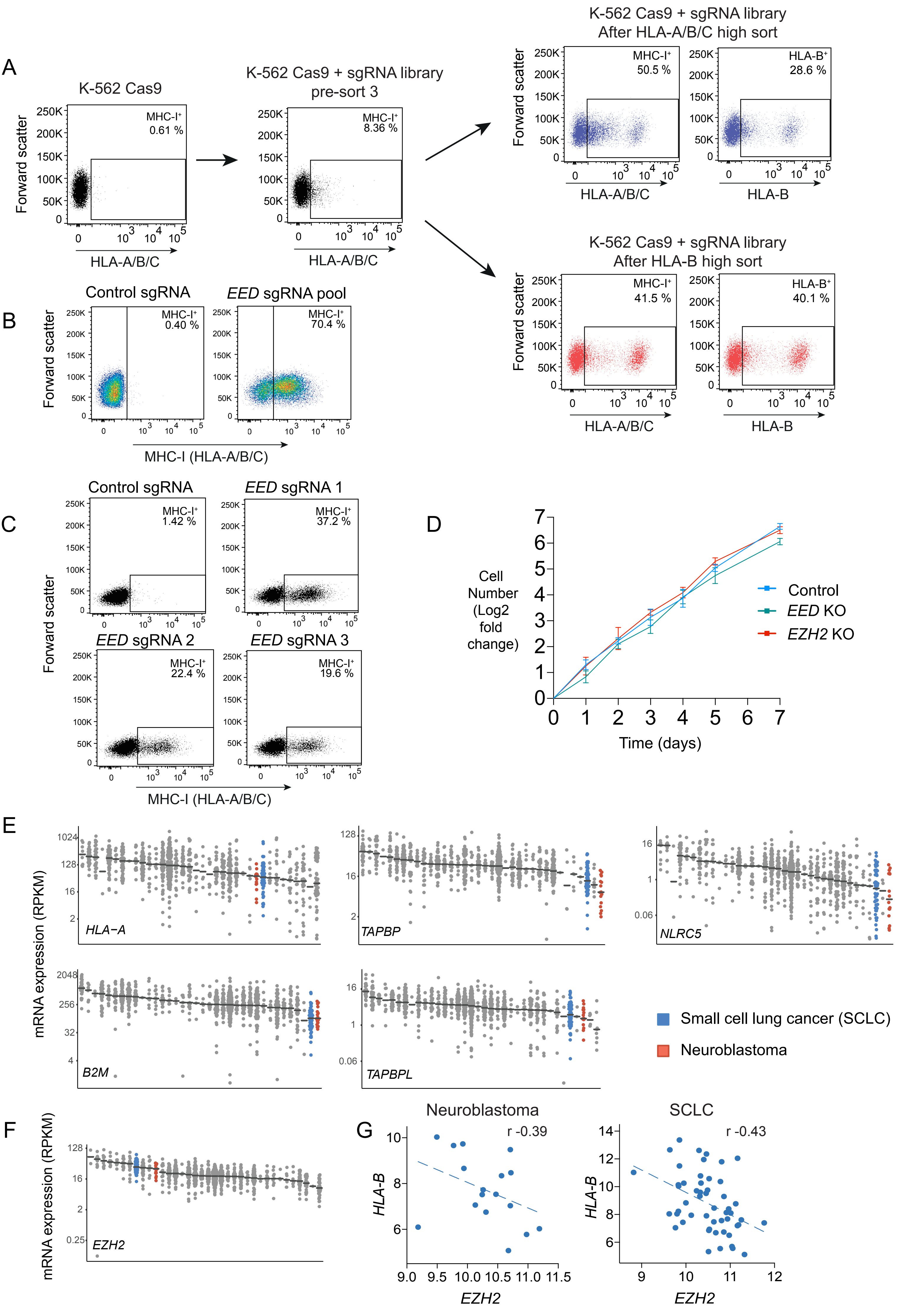
A
K-562 Cas9
MHC-I+ 0.61 %
K-562 Cas9 + sgRNA library pre-sort 3
MHC-I+ 8.36 %
K-562 Cas9 + sgRNA library After HLA-A/B/C high sort
MHC-I+ 50.5 %
HLA-B+ 28.6 %
K-562 Cas9 + sgRNA library After HLA-B high sort
MHC-I+ 41.5 %
HLA-B+ 40.1 %
Forward scatter
HLA-A/B/C
HLA-B
B
Control sgRNA
MHC-I+ 0.40 %
EED sgRNA pool
MHC-I+ 70.4 %
MHC-I (HLA-A/B/C)
C
Control sgRNA
MHC-I+ 1.42 %
EED sgRNA 1
MHC-I+ 37.2 %
EED sgRNA 2
MHC-I+ 22.4 %
EED sgRNA 3
MHC-I+ 19.6 %
MHC-I (HLA-A/B/C)
D
Cell Number (Log2 fold change)
Time (days)
Control
EED KO
EZH2 KO
E
mRNA expression (RPKM)
HLA−A
TAPBP
NLRC5
B2M
TAPBPL
Small cell lung cancer (SCLC)
Neuroblastoma
F
mRNA expression (RPKM)
EZH2
G
Neuroblastoma
r -0.39
SCLC
r -0.43
HLA-B
EZH2

Figure S1. Related to Figure 1. PRC2 components maintain silencing of MHC-I expression in MHC-I deficient cancers. (A) K-562 cells stably expressing Cas9 were mutagenized by infection with a pooled lentiviral sgRNA library and rare MHC-I high cells were enriched by 3 successive rounds of FACS sorting for mCherry positive (containing sgRNA vector) MHC-I positive cells. FACS plots show MHC-I expression in: unsorted K-562 Cas9 cells (left), K-562 Cas9 cells transduced with the CRISPR sgRNA library before the third sort enrichment (middle), and the same cells after sort enrichment with either a pan-HLA-A,B,C antibody (top right panels) or with an HLA-B specific antibody (bottom right panels). (B) Cell surface MHC-I in K-562 Cas9 cells transduced with a pool of 3 sgRNAs targeting *EED* or a control sgRNA. (C) Cell surface MHC-I in K-562 Cas9 cells transduced with individual sgRNAs targeting *EED* or a control sgRNA. (D) *In vitro* proliferation of *EED* and *EZH2* KO K-562 clones compared to control parental cells. Graph shows mean and SEM log2 fold change in cell number compared to baseline from 4 experiments. (E and F) mRNA expression of MHC-I genes (E) and *EZH2* (F) in 920 cell lines in Cancer Cell Line Encyclopedia. Each dot represents an individual cancer cell line, clustered by tumor type (RPKM log2 scale). Black line indicates median. (G) Correlation plot showing mRNA expression data for *EZH2* and *HLA-B* in neuroblastoma and SCLC from Cancer Cell Line Encyclopedia (accessed via http://www.cbioportal.org/). Pearson's correlation coefficient (r).

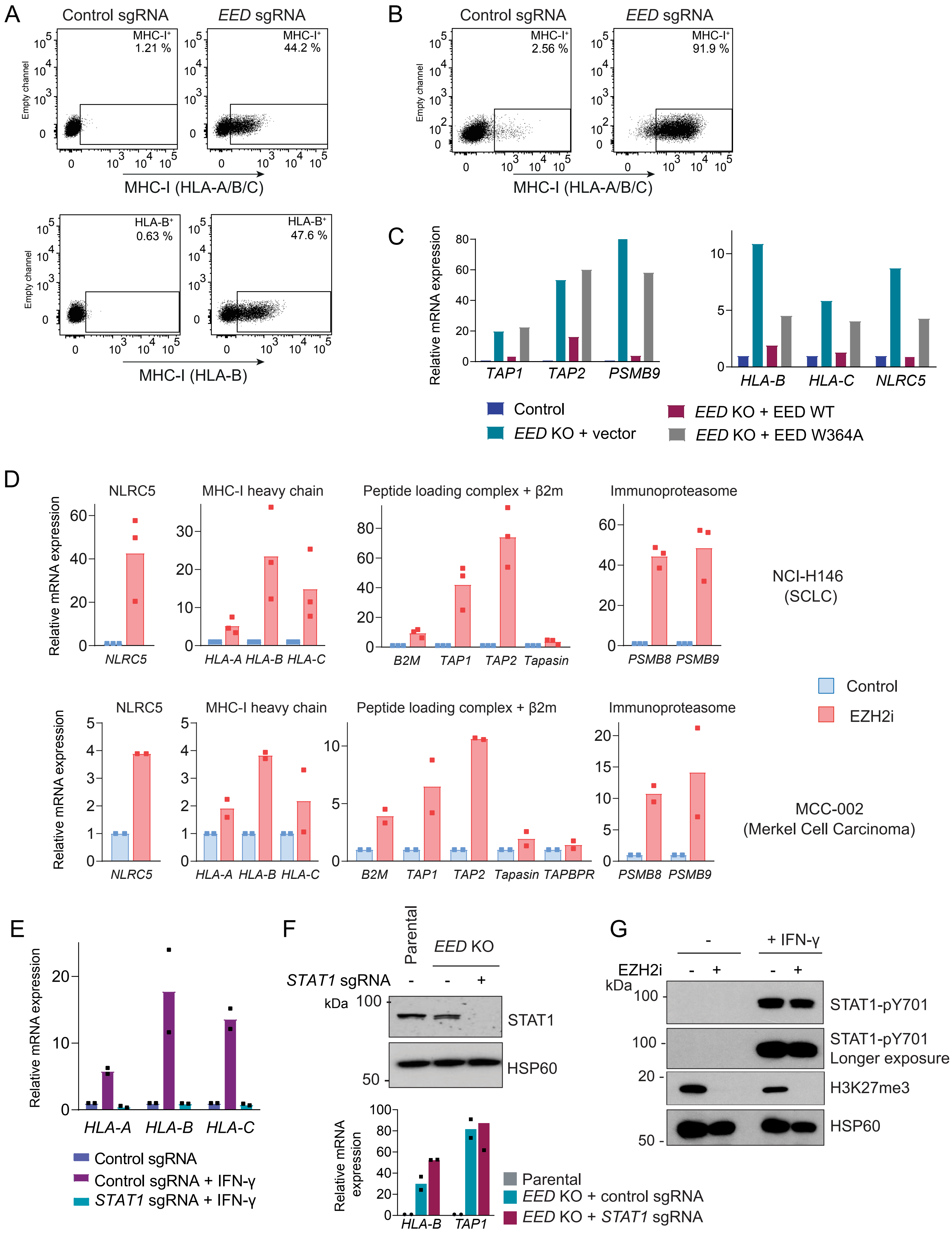
A
Control sgRNA
EED sgRNA
MHC-I+ 1.21 %
MHC-I+ 44.2 %
Empty channel
MHC-I (HLA-A/B/C)
HLA-B+ 0.63 %
HLA-B+ 47.6 %
MHC-I (HLA-B)
B
Control sgRNA
EED sgRNA
MHC-I+ 2.56 %
MHC-I+ 91.9 %
Empty channel
MHC-I (HLA-A/B/C)
C
Relative mRNA expression
TAP1 TAP2 PSMB9
HLA-B HLA-C NLRC5
Control
EED KO + vector
EED KO + EED WT
EED KO + EED W364A
D
NLRC5
MHC-I heavy chain
Peptide loading complex + β2m
Immunoproteasome
Relative mRNA expression
NLRC5
HLA-A HLA-B HLA-C
B2M TAP1 TAP2 Tapasin
PSMB8 PSMB9
NCI-H146 (SCLC)
Control
EZH2i
B2M TAP1 TAP2 Tapasin TAPBPR
MCC-002 (Merkel Cell Carcinoma)
E
Relative mRNA expression
HLA-A HLA-B HLA-C
Control sgRNA
Control sgRNA + IFN-γ
STAT1 sgRNA + IFN-γ
F
Parental
EED KO
STAT1 sgRNA
kDa
STAT1
HSP60
Relative mRNA expression
HLA-B TAP1
Parental
EED KO + control sgRNA
EED KO + STAT1 sgRNA
G
+ IFN-γ
EZH2i
kDa
STAT1-pY701
STAT1-pY701 Longer exposure
H3K27me3
HSP60

Figure S2. Related to Figure 2. Genetic disruption or pharmacological inhibition of PRC2 restores MHC-I expression in MHC-I low cancers. (A and B) Cell surface MHC-I in Cas9 expressing human neuroblastoma (Kelly) (A) or SCLC (NCI-H146) (B) following transduction with a pool of 2 sgRNAs targeting *EED* compared to control sgRNA. (C) qRT-PCR analysis of MHC-I gene expression in *EED* KO K-562 cells transduced with lentiviral vectors encoding wild-type EED, EED W364A or GFP (vector). Expression of MHC-I APP genes relative to control parental K-562 Cas9 cells. Bars show mean of technical triplicates from a representative experiment. (D) qRT-PCR analysis of MHC-I gene expression in cells treated with EZH2 inhibitor (EZH2i), GSK-503 (5 µM) in NCI-H146 and EPZ-011989 (3 µM) in MCC-002, compared to DMSO or ethanol (ETOH) treated control. Analyses performed after 10 days of treatment. (E) qRT-PCR analysis of K-562 Cas9 cells expressing *STAT1* targeting or control sgRNA treated with or without IFN-γ 10 ng/ml for 24 hr. (F) *EED* knockout K-562 Cas9 cells were transduced with control or *STAT1* targeting sgRNA and analyzed by immunoblot and qRT-PCR compared to parental K-562 Cas9 cells. (G) Immunoblot analysis of K-562 cells treated with EPZ-011989 3 µM or ETOH control for 10 days and pulsed with or without IFN-γ 10 ng/ml for 24 hr. For D, E and F, bars indicate the mean fold change from independent experiments and points denote the mean of technical triplicates from individual experiments.

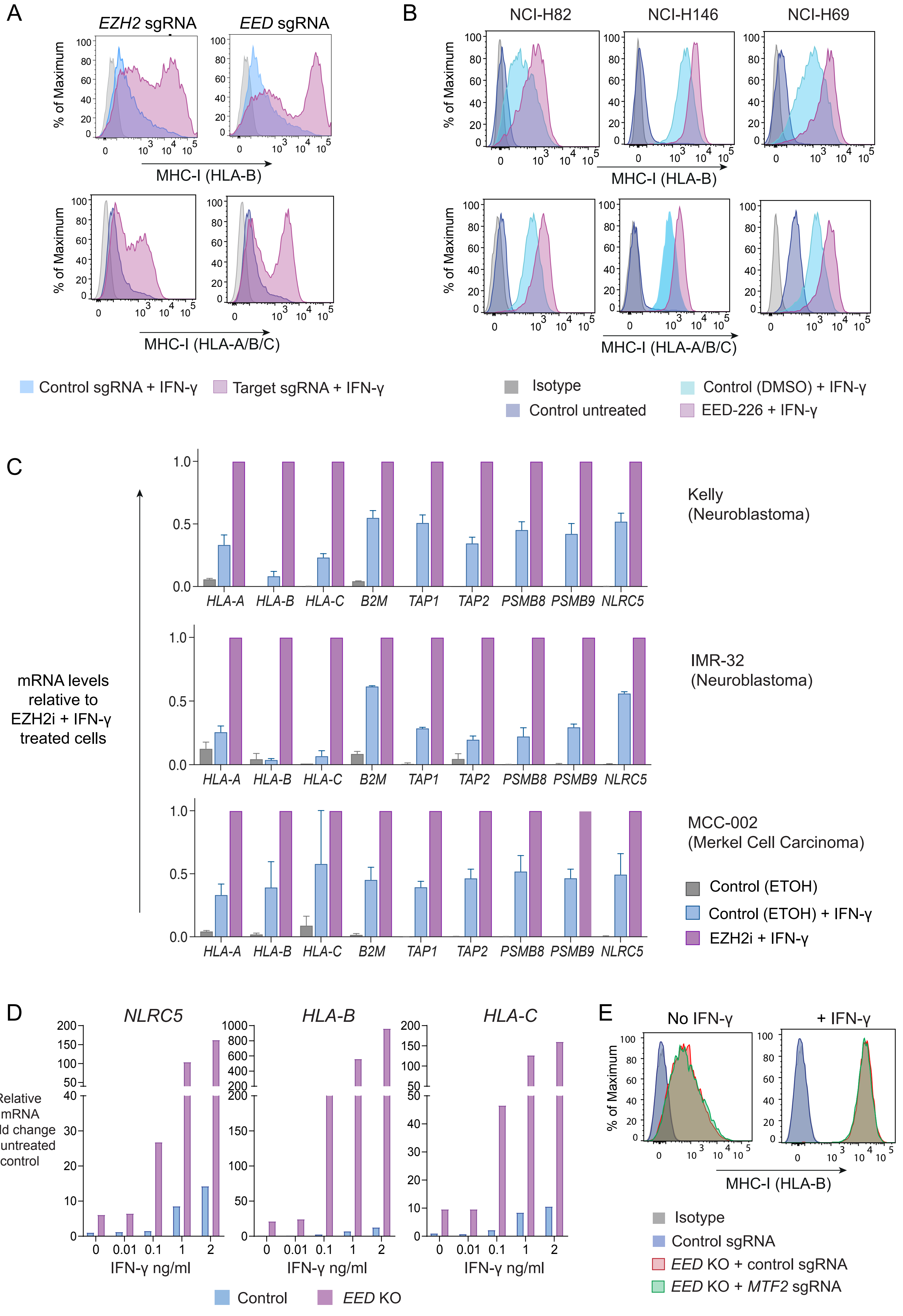
A
EZH2 sgRNA
EED sgRNA
% of Maximum
MHC-I (HLA-B)
MHC-I (HLA-A/B/C)
Control sgRNA + IFN-γ
Target sgRNA + IFN-γ
B
NCI-H82
NCI-H146
NCI-H69
% of Maximum
MHC-I (HLA-B)
MHC-I (HLA-A/B/C)
Isotype
Control untreated
Control (DMSO) + IFN-γ
EED-226 + IFN-γ
C
mRNA levels relative to EZH2i + IFN-γ treated cells
HLA-A HLA-B HLA-C B2M TAP1 TAP2 PSMB8 PSMB9 NLRC5
Kelly (Neuroblastoma)
IMR-32 (Neuroblastoma)
MCC-002 (Merkel Cell Carcinoma)
Control (ETOH)
Control (ETOH) + IFN-γ
EZH2i + IFN-γ
D
NLRC5
HLA-B
HLA-C
Relative mRNA Fold change vs untreated control
IFN-γ ng/ml
Control
EED KO
E
No IFN-γ
+ IFN-γ
% of Maximum
MHC-I (HLA-B)
Isotype
Control sgRNA
EED KO + control sgRNA
EED KO + MTF2 sgRNA

Figure S3. Related to Figure 3. Loss of PRC2 function augments IFN-γ induced MHC-I expression in MHC-I low cancers. (A) Cell surface MHC-I in K-562 Cas9 cells transduced with sgRNA targeting *EED* or *EZH2*, or control sgRNA, and pulsed with IFN-γ 10 ng/ml for 24 hr. (B) Cell surface MHC-I in human SCLC incubated with EED inhibitor (EED-226 5 μM) or DMSO control for 10 days and pulsed with IFN-γ 10 ng/ml for 24 hr. (C) qRT-PCR analysis in the indicated human cancer cells treated with EPZ-011989 3 μM or ETOH control for 10 days and pulsed with IFN-γ 10 ng/ml for 24 hr. mRNA levels are expressed proportional to expression in cells treated with combination of EZH2i and IFN-γ. Graphs show mean ± SEM from independent experiments. (D) qRT-PCR analysis of MHC-I gene expression in *EED* KO or parental control K-562 cells incubated with the indicated concentrations of IFN-γ for 24 hr. (E) Cell surface HLA-B in *EED* knockout K-562 Cas9 cells expressing control sgRNA or two *MTF2*-specific sgRNAs. In right panel, cells were incubated with IFN-γ 10 ng/ml for 24 hr.

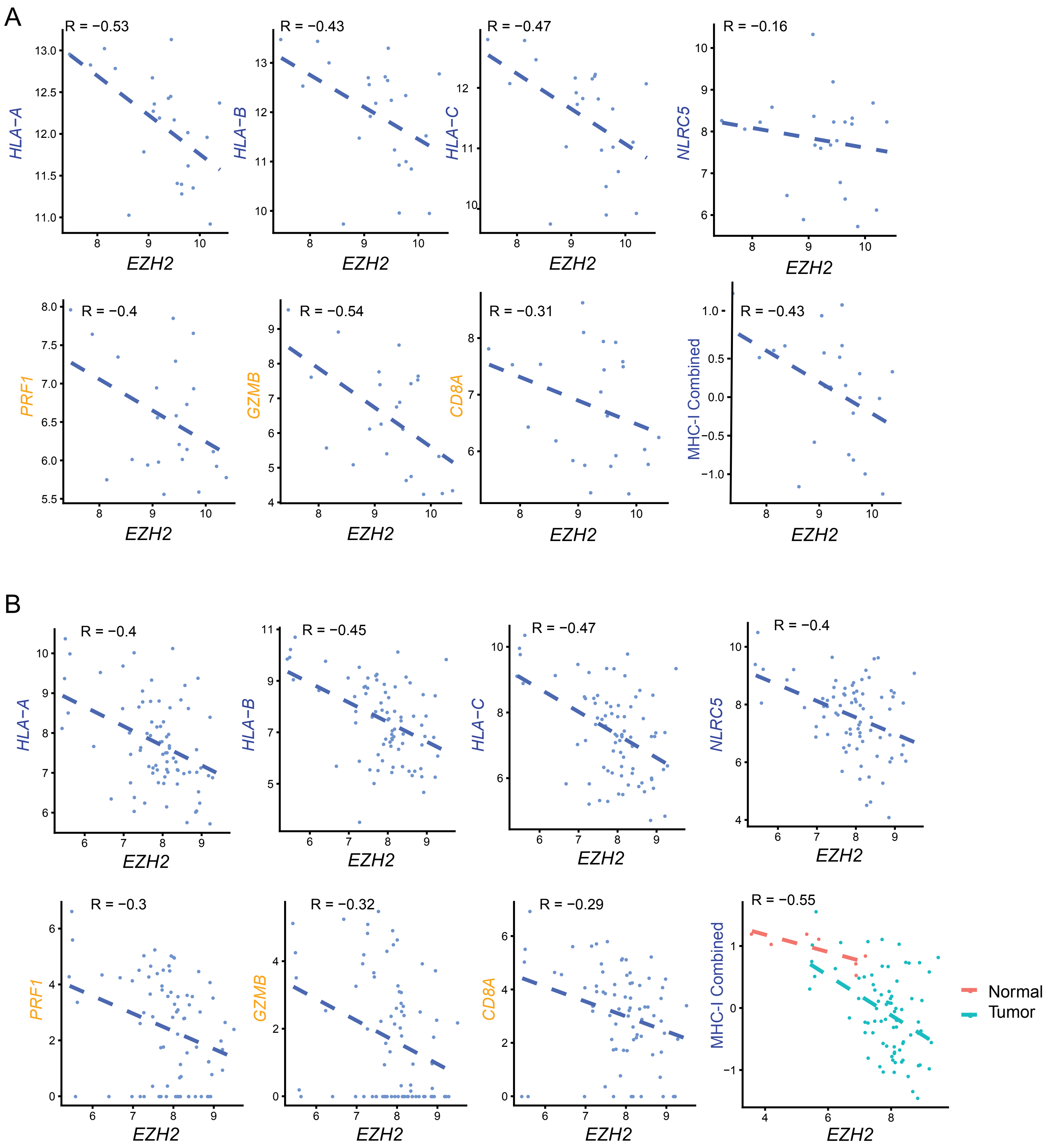
A
R = −0.53
HLA−A
R = −0.43
HLA−B
R = −0.47
HLA−C
R = −0.16
NLRC5
R = −0.4
PRF1
R = −0.54
GZMB
R = −0.31
CD8A
R = −0.43
MHC-I Combined
EZH2
B
R = −0.4
HLA−A
R = −0.45
HLA−B
R = −0.47
HLA−C
R = −0.4
NLRC5
R = −0.3
PRF1
R = −0.32
GZMB
R = −0.29
CD8A
R = −0.55
MHC-I Combined
Normal
Tumor
EZH2

Figure S4. Related to Figures 4 and 6. *EZH2* expression is negatively correlated with expression of MHC-I genes and markers of CD8$^+$ T cells in primary human SCLC tumor samples. (A and B) Correlation plots show expression of MHC-I genes, T cell genes and *EZH2* in 23 primary SCLC tumor samples analyzed using Affymetrix Human Genome Array (A) and 79 primary SCLC tumors and 7 normal lung tissue samples analyzed by RNA-sequencing (B). Plots show normalized log2 mRNA expression of the indicated genes and the Pearson's correlation coefficient (R). The combined MHC-I score represents the mean normalized log2 expression of multiple MHC-I genes (*HLA-A, HLA-B, HLA-C, TAP1, TAP2, TAPBP, B2M, NLRC5, PSMB8, PSMB9, ERAP1, ERAP2*). MHC-I genes are indicated in blue and CD8$^+$ T cell genes in orange. *PRF1* (perforin); *GZMB* (granzyme B). Data in A are from GSE43346 (Sato et al, 2013) and data in B are from GSE60052 (Jiang et al, 2016).

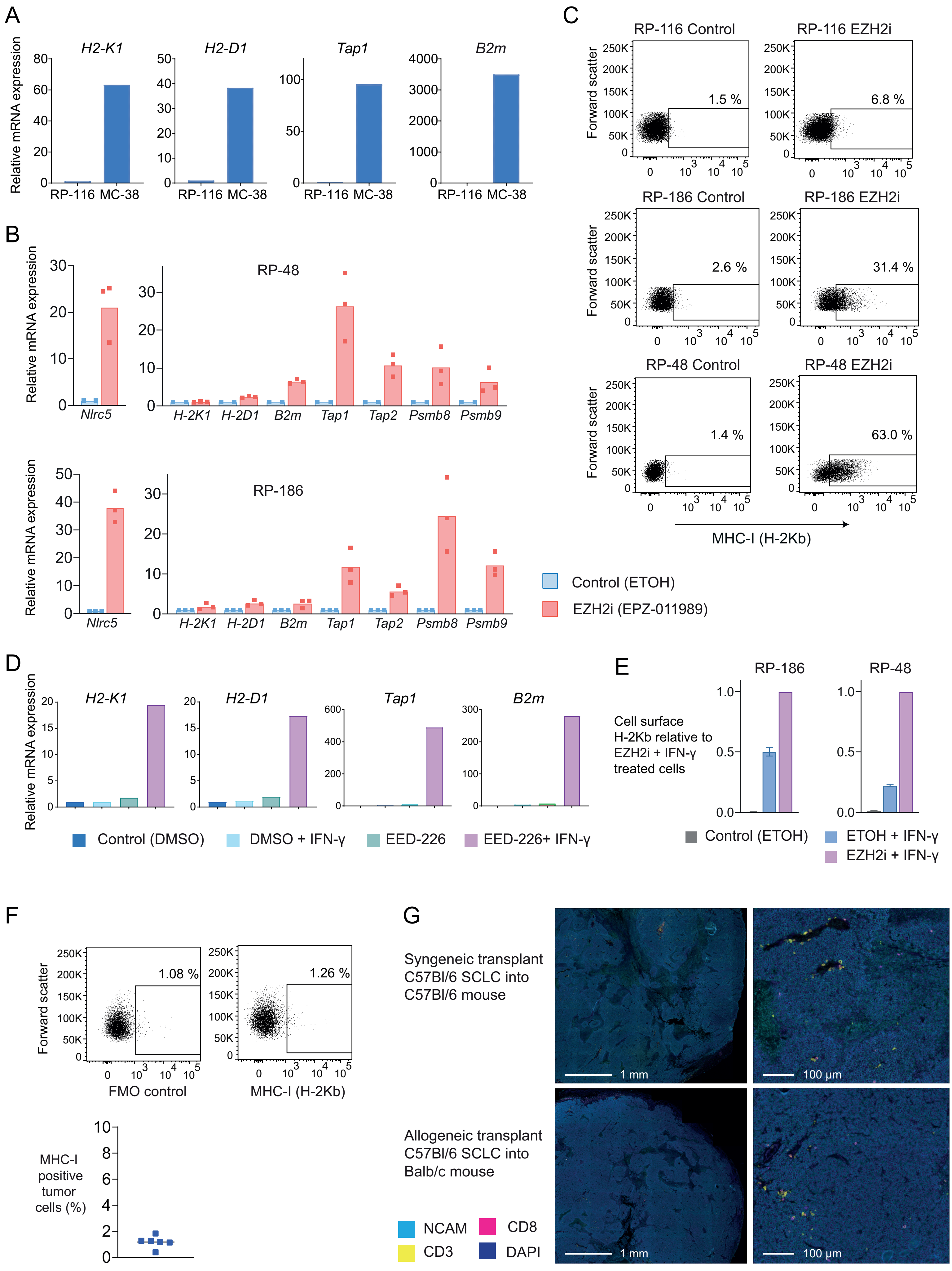

A
Relative mRNA expression
H2-K1
H2-D1
Tap1
B2m
RP-116 MC-38
B
RP-48
RP-186
Nlrc5
H-2K1 H-2D1 B2m Tap1 Tap2 Psmb8 Psmb9
Control (ETOH)
EZH2i (EPZ-011989)
C
RP-116 Control
RP-116 EZH2i
RP-186 Control
RP-186 EZH2i
RP-48 Control
RP-48 EZH2i
Forward scatter
1.5 %
6.8 %
2.6 %
31.4 %
1.4 %
63.0 %
MHC-I (H-2Kb)
D
Control (DMSO)
DMSO + IFN-γ
EED-226
EED-226+ IFN-γ
E
RP-186
RP-48
Cell surface H-2Kb relative to EZH2i + IFN-γ treated cells
Control (ETOH)
ETOH + IFN-γ
EZH2i + IFN-γ
F
1.08 %
1.26 %
FMO control
MHC-I (H-2Kb)
MHC-I positive tumor cells (%)
G
Syngeneic transplant C57Bl/6 SCLC into C57Bl/6 mouse
Allogeneic transplant C57Bl/6 SCLC into Balb/c mouse
1 mm
100 µm
NCAM
CD8
CD3
DAPI

Figure S5. Related to Figures 4 and 6. PRC2 silences MHC-I expression in mouse *Trp53⁻ Rb1⁻* SCLC tumors. (A) qRT-PCR analysis of MHC-I gene expression in mouse SCLC RP-116 cells and mouse colorectal carcinoma MC-38 cells. Bars depict mean fold difference in mRNA expression in MC-38 relative to RP-116 from a representative experiment**.** (B and C) qRT-PCR (B) and flow cytometry analysis (C) of MHC-I expression in the indicated mSCLC cells treated with EZH2i (EPZ-011989 3 µM) or ETOH control. Analysis after 10 days of treatment. For B, bars show mean fold change in mRNA expression compared to control from 3 experiments and individual points denote the mean of technical triplicates from each experiment. (D) qRT-PCR analysis in RP-116 cells treated with EED-226 5 µM or DMSO control for 10 days and pulsed with or without IFN-γ 10 ng/ml for 24 hr. (E) Flow cytometry analysis of cell surface MHC-I (H-2Kb) in mSCLC RP-186 and RP-48. Cells treated with EZH2i (EPZ-011989 3 µM) or ETOH control for 10 days and pulsed with IFN-γ 10 ng/ml for 24 hr. Relative MFI is expressed proportional to MFI of cells treated with combination of EZH2i and IFN-γ. Mean ± SEM of 3 experiments. (F) Cell surface MHC-I expression in mouse SCLC (RP-116) tumors harvested and digested following subcutaneous transplant into C57Bl/6 mice. Tumor cells were identified as the viable cells negative for CD45, CD31 and Ter199 and MHC-I positive cells were gated with reference to fluorescence minus one (FMO) control. Graph shows results from 6 mice. Horizontal black line indicates mean. (G) Multiplex immunohistochemistry of mSCLC RP-116 tumors transplanted into C57Bl/6 or Balb/c mice. Tumors were harvested and fixed at a size of 500 mm^3. NCAM marks SCLC tumor cells and T cells were stained with CD3 and CD8. Representative images of intratumoral staining are shown.

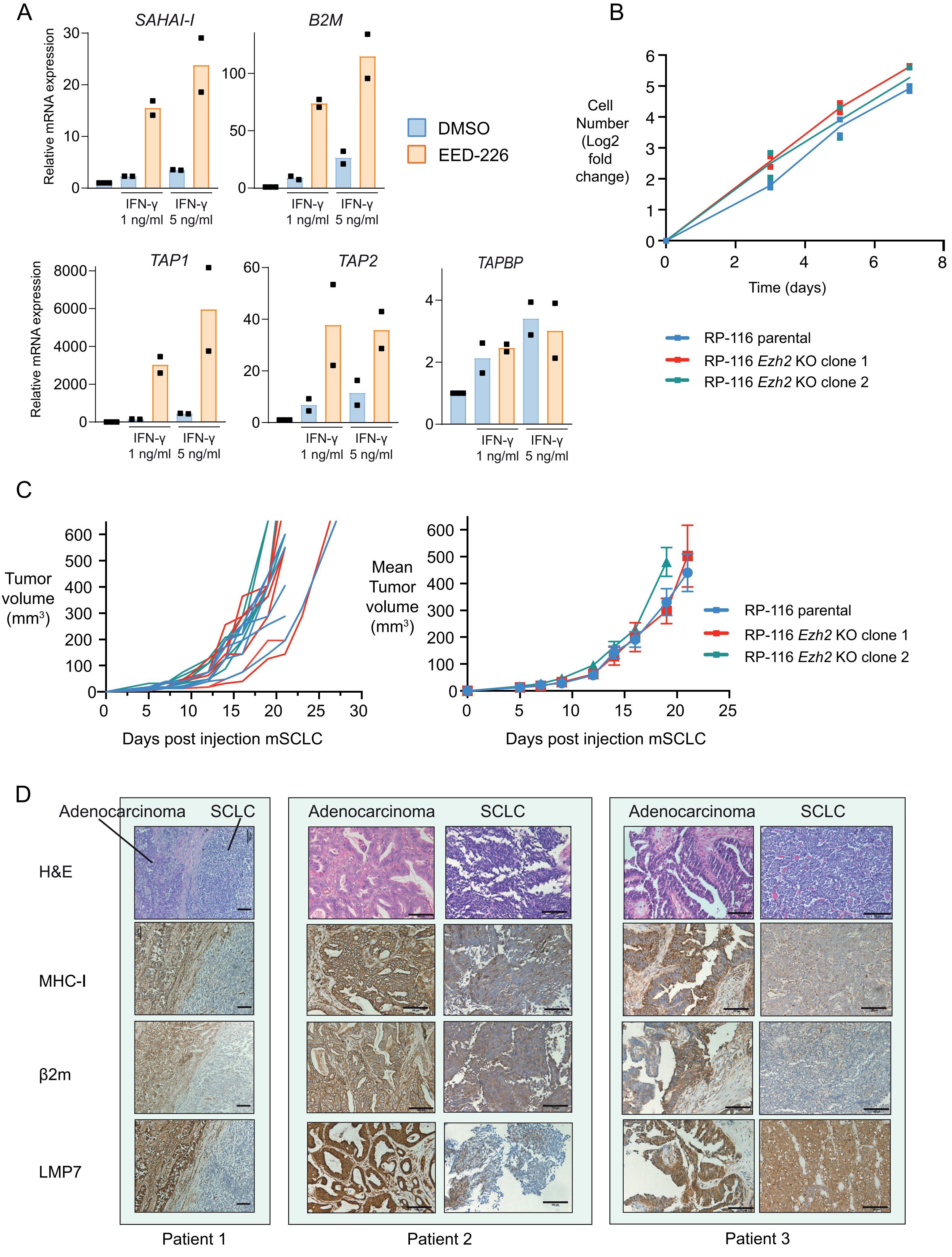
A
SAHAI-I
B2M
TAP1
TAP2
TAPBP
Relative mRNA expression
IFN-γ 1 ng/ml
IFN-γ 5 ng/ml
DMSO
EED-226
B
Cell Number (Log2 fold change)
Time (days)
RP-116 parental
RP-116 Ezh2 KO clone 1
RP-116 Ezh2 KO clone 2
C
Tumor volume (mm³)
Days post injection mSCLC
Mean Tumor volume (mm³)
D
Adenocarcinoma
SCLC
H&E
MHC-I
β2m
LMP7
Patient 1
Patient 2
Patient 3

Figure S6. Related to Figure 6. PRC2-mediated silencing of MHC-I antigen presentation promotes immune evasion. (A) qRT-PCR analysis of MHC-I gene expression in DFT1 cells C5065 treated with EED-226 3 μM or DMSO control for 10 days and pulsed with the indicated doses of devil IFN-γ for 6 hr. Bars denote mean fold change in mRNA expression relative to non-interferon treated DMSO control from independent experiments and individual points denote the mean of technical triplicates from each experiment. *SAHAI-1* encodes Tasmanian devil MHC-I. (B) Proliferation of parental RP-116 and RP-116 *Ezh2* knockout clones *in vitro*. Line indicates mean log2 fold change in cell number compared to baseline. Individual points indicate the results of independent experiments. (C) mSCLC tumor growth following subcutaneous transplant of 10^6 parental or *Ezh2* knockout RP-116 cells into NOD-scid IL2Rγ^{null} mice. Endpoint at a tumor volume of 500 mm^3. 6 mice per group. Right panel shows mean ± SEM of tumor growth. *Ezh2* KO clone 1 used for experiment in Figure 6C and 6D. (D) Immunohistochemistry for MHC-I, β2m and LMP7 (immunoproteasome) in patients with transformation of EGFR mutant lung adenocarcinoma to SCLC. Patient 1 was described in Figure 6E, additional staining of post-mortem tumor samples is shown. Clinical information relating to patient 2 and patient 3 is described in the methods. All scale bars are 100 μm.

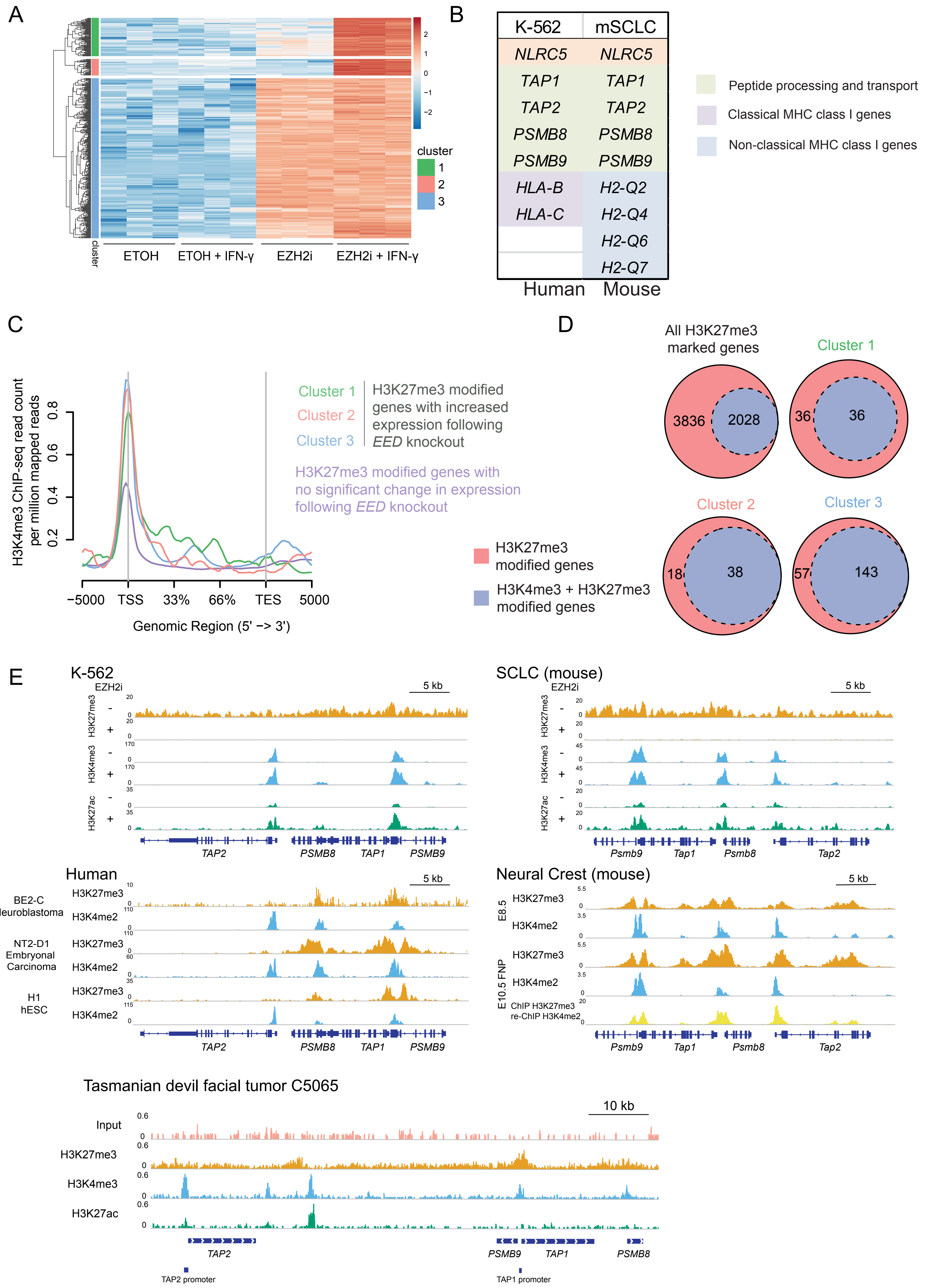

A
B
C
D
E
ETOH
ETOH + IFN-γ
EZH2i
EZH2i + IFN-γ
cluster
K-562
mSCLC
NLRC5
TAP1
TAP2
PSMB8
PSMB9
HLA-B
HLA-C
H2-Q2
H2-Q4
H2-Q6
H2-Q7
Human
Mouse
Peptide processing and transport
Classical MHC class I genes
Non-classical MHC class I genes
H3K4me3 ChIP-seq read count per million mapped reads
Genomic Region (5' -> 3')
Cluster 1
Cluster 2
Cluster 3
H3K27me3 modified genes with increased expression following EED knockout
H3K27me3 modified genes with no significant change in expression following EED knockout
All H3K27me3 marked genes
3836
2028
36
36
18
38
57
143
H3K27me3 modified genes
H3K4me3 + H3K27me3 modified genes
K-562
SCLC (mouse)
Human
Neural Crest (mouse)
BE2-C Neuroblastoma
NT2-D1 Embryonal Carcinoma
H1 hESC
E8.5
E10.5 FNP
ChIP H3K27me3 re-ChIP H3K4me2
Tasmanian devil facial tumor C5065
Input
H3K27me3
H3K4me3
H3K27ac
H3K4me2
TAP2
PSMB8
TAP1
PSMB9
Psmb9
Tap1
Psmb8
Tap2
TAP2 promoter
TAP1 promoter
5 kb
10 kb

Figure S7. Related to Figure 7. An evolutionarily conserved function of PRC2 maintains bivalency at MHC-I gene promoters in MHC-I low cancers. (A) Heatmap displaying H3K27me3 modified genes upregulated by > 1.5 log2 fold change in mouse SCLC cells (RP-116) treated with EPZ-011989 3 μM for 10 days and pulsed with IFN-γ 10 ng/ml compared to ETOH treated control cells. Genes were clustered using Euclidean clustering, and clusters were separated using a kmeans of 3. Red indicates higher expression and blue indicates lower expression. (B) H3K27me3 modified MHC-I genes identified in clusters 2 and 3 in either K-562 cells or mSCLC cells. (C) H3K4me3 average ChIP-seq read density profiles in H3K27me3 modified genes derepressed following *EED* depletion in K-562 cells (all genes in heatmap in figure 7A, separated by cluster) compared to the H3K4me3 read density profile of H3K27me3 modified genes that are not induced. (D) Venn diagrams show the number H3K27me3 modified genes with concurrent H3K4me3 modification (purple) identified within each cluster of the heatmap in K-562 cells. (E) Bivalent H3K27me3 and H3K4me3 modification of MHC-I gene promoters in human, mouse and Tasmanian devil cells and mouse neural crest cells. K-562, mSCLC (RP-116) and Tasmanian devil ChIP-seq was performed in house. K-562 and mSCLC were treated with EPZ-011989 3 μM or ETOH control for 10 days prior to harvest. Neural crest data from GSE89435 (Minoux et al., 2017), BE2-C (neuroblastoma) from GSE80151 (Zeid et al., 2018), H1-hESC from ENCODE (https://www.encodeproject.org/).

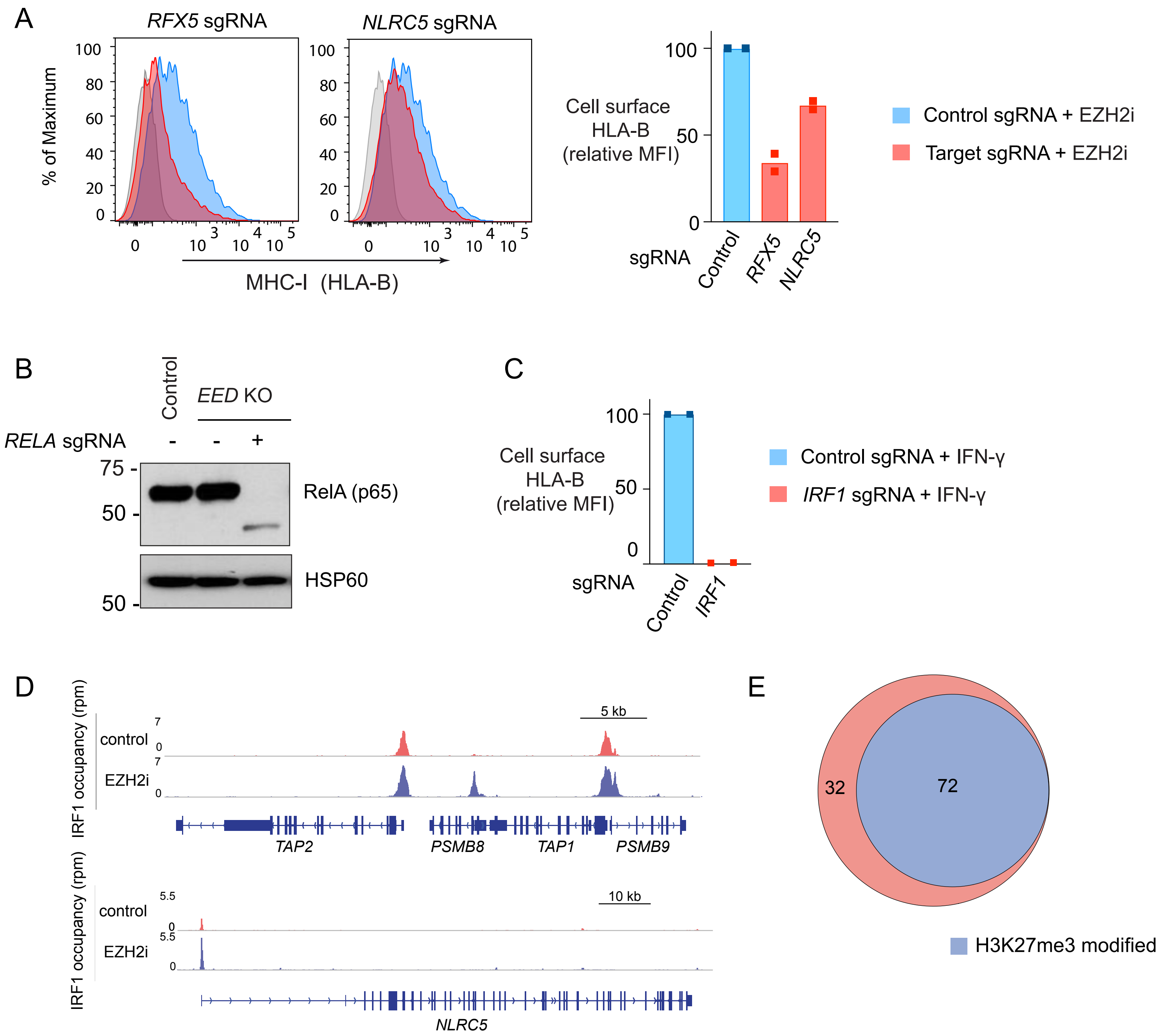

A
RFX5 sgRNA
NLRC5 sgRNA
% of Maximum
MHC-I (HLA-B)
Cell surface HLA-B (relative MFI)
sgRNA
Control
RFX5
NLRC5
Control sgRNA + EZH2i
Target sgRNA + EZH2i
B
Control
EED KO
RELA sgRNA
RelA (p65)
HSP60
C
Cell surface HLA-B (relative MFI)
sgRNA
Control
IRF1
Control sgRNA + IFN-γ
IRF1 sgRNA + IFN-γ
D
IRF1 occupancy (rpm)
control
EZH2i
5 kb
TAP2
PSMB8
TAP1
PSMB9
10 kb
NLRC5
E
32
72
H3K27me3 modified

Figure S8. Related to Figure 8. Loss of PRC2 provides a permissive chromatin environment to facilitate MHC-I expression. (A) Kelly Cas9 cells transduced with either control, *NLRC5*- or *RFX5*-specific sgRNA were treated with EPZ-011989 3 μM for 10 days prior to analysis for cell surface HLA-B. Bars represent mean fold change in HLA-B mean fluorescence intensity (MFI) in *RFX5* or *NLRC5* depleted cells compared to control cells from independent experiments, indicated by individual points. (B) RelA immunoblot in *EED* knockout K-562 Cas9 cells transduced with control or *RELA*-specific sgRNA. (C) Cell surface HLA-B in K-562 Cas9 cells expressing control or *IRF1*-specific sgRNA following incubation with IFN-γ 10 ng/ml for 24 hr. Bars represent mean fold change in HLA-B MFI from independent experiments. (D) IRF1 occupancy at MHC-I APP genes. ChIP-seq using an IRF1-specific antibody in K-562 cells treated with EPZ-011989 3μM or ETOH control for 10 days. (E) Venn diagram contains genes showing increased IRF1 binding (fold increase > 4 at tss) in EZH2i treated compared to control cells (n = 104). The number of these genes that were H3K27me3 modified is indicated in purple (n=72). Overall, IRF1 binding was detected at the tss of 1169 genes, of which 161 were H3K27me3 modified. IRF1 and H3K27me3 ChIP-seq were performed in K-562 cells treated with EPZ-011989 3μM or ETOH control for 10 days.
