# Supplementary material for: An Evolutionarily Conserved Function of Polycomb Silences the MHC Class I Antigen Presentation Pathway and Enables Immune Evasion in Cancer
Source: Cancer Cell. 2019 Oct 14;36(4):385–401.e8. doi: 10.1016/j.ccell.2019.08.008 (PMC6876280; doi:10.1016/j.ccell.2019.08.008)
Supplement: Document S2. Article plus Supplemental Information [file mmc5.pdf]

Article

Cancer Cell

An Evolutionarily Conserved Function of Polycomb Silences the MHC Class I Antigen Presentation Pathway and Enables Immune Evasion in Cancer

Graphical Abstract

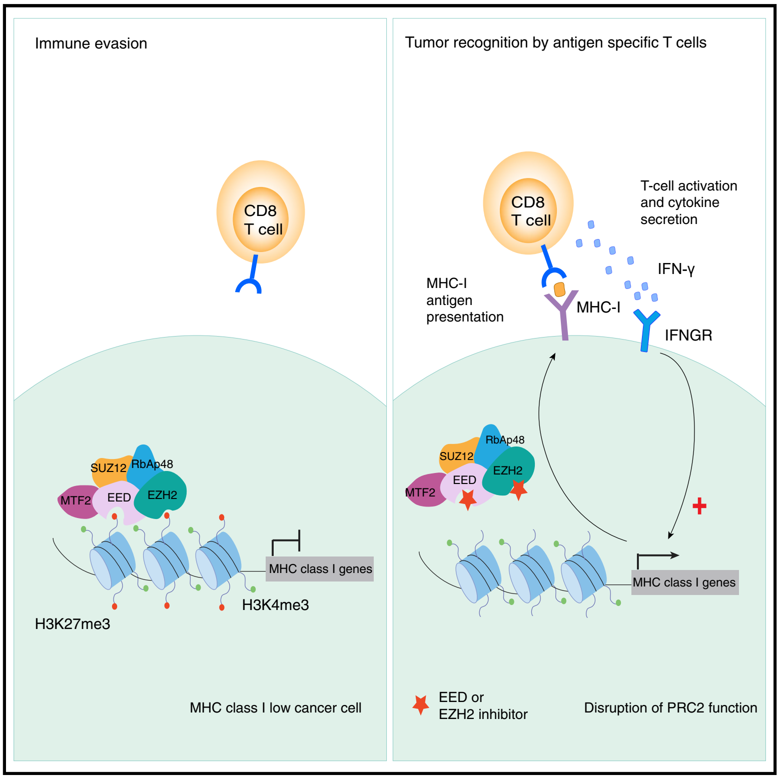

Highlights

- PRC2 maintains bivalency at MHC-I antigen-processing genes silencing MHC-I expression
- Cancer cells co-opt this conserved, lineage-specific PRC2 function to evade T cells
- Pharmacological inhibition of PRC2 in MHC-I low cancers restores anti-tumor immunity
- Immunotherapy resistance may arise via non-genomic routes that exploit PRC2 activity

Authors

Marian L. Burr, Christina E. Sparbier, Kah Lok Chan, ..., Paul J. Lehner, Kate D. Sutherland, Mark A. Dawson

Correspondence

marian.burr@petermac.org (M.L.B.), mark.dawson@petermac.org (M.A.D.)

In Brief

Burr et al. show that cancer cells co-opt PRC2 to evade immune surveillance. PRC2 maintains bivalency at the promoters of MHC-I antigen-processing pathway (MHC-I APP) genes to repress their basal and cytokine-activated expression. Inhibition of PRC2 restores the MHC-I APP and T cell-mediated anti-tumor immunity.

Burr et al., 2019, Cancer Cell *36*, 385–401
October 14, 2019 © 2019 The Authors. Published by Elsevier Inc.
https://doi.org/10.1016/j.ccell.2019.08.008

CellPress

An Evolutionarily Conserved Function of Polycomb Silences the MHC Class I Antigen Presentation Pathway and Enables Immune Evasion in Cancer

Marian L. Burr,[1,2,3,*] Christina E. Sparbier,[1,2,12] Kah Lok Chan,[1,2,12] Yih-Chih Chan,[1] Ariena Kersbergen,[4] Enid Y.N. Lam,[1,2] Elizabeth Azidis-Yates,[1] Dane Vassiliadis,[1,2] Charles C. Bell,[1,2] Omer Gilan,[1,2] Susan Jackson,[1] Lavinia Tan,[1,2] Stephen Q. Wong,[1,2] Sebastian Hollizeck,[1,2] Ewa M. Michalak,[1,2] Hannah V. Siddle,[5,11] Michael T. McCabe,[6] Rab K. Prinjha,[6,7] Glen R. Guerra,[1,2] Benjamin J. Solomon,[1,2] Shahneen Sandhu,[1,2] Sarah-Jane Dawson,[1,2,8] Paul A. Beavis,[1,2] Richard W. Tothill,[2,8,9] Carleen Cullinane,[1,2] Paul J. Lehner,[3] Kate D. Sutherland,[4,10] and Mark A. Dawson[1,2,8,13,*]
[1]Peter MacCallum Cancer Centre, 305 Grattan Street, Melbourne, VIC 3000, Australia
[2]Sir Peter MacCallum Department of Oncology, University of Melbourne, Parkville, VIC 3052, Australia
[3]Cambridge Institute for Medical Research, Cambridge Biomedical Campus, Hills Road, Cambridge CB2 0XY, UK
[4]ACRF Cancer Biology and Stem Cell Division, Walter and Eliza Hall Institute of Medical Research, Parkville, VIC 3052, Australia
[5]Department of Biological Sciences, University of Southampton, Southampton, UK
[6]Epigenetics Research Unit, Oncology R&D, GlaxoSmithKline, Collegeville, PA, USA
[7]Epigenetics Research Unit, GlaxoSmithKline, Stevenage, UK
[8]Centre for Cancer Research, University of Melbourne, Parkville, Australia
[9]Department of Clinical Pathology, University of Melbourne, Melbourne, VIC, Australia
[10]Department of Medical Biology, The University of Melbourne, Parkville, VIC 3010, Australia
[11]Institute for Life Sciences, University of Southampton, Southampton, UK
[12]These authors contributed equally
[13]Lead Contact
*Correspondence: marian.burr@petermac.org (M.L.B.), mark.dawson@petermac.org (M.A.D.)
https://doi.org/10.1016/j.ccell.2019.08.008

SUMMARY

Loss of MHC class I (MHC-I) antigen presentation in cancer cells can elicit immunotherapy resistance. A genome-wide CRISPR/Cas9 screen identified an evolutionarily conserved function of polycomb repressive complex 2 (PRC2) that mediates coordinated transcriptional silencing of the MHC-I antigen processing pathway (MHC-I APP), promoting evasion of T cell-mediated immunity. MHC-I APP gene promoters in MHC-I low cancers harbor bivalent activating H3K4me3 and repressive H3K27me3 histone modifications, silencing basal MHC-I expression and restricting cytokine-induced upregulation. Bivalent chromatin at MHC-I APP genes is a normal developmental process active in embryonic stem cells and maintained during neural progenitor differentiation. This physiological MHC-I silencing highlights a conserved mechanism by which cancers arising from these primitive tissues exploit PRC2 activity to enable immune evasion.

INTRODUCTION

Cancer immune surveillance requires co-operation of innate and adaptive immune systems to eradicate developing tumors. The key effectors of tumor elimination are CD8$^+$ cytotoxic T cells that recognize and kill cells displaying foreign antigens bound to MHC class I (MHC-I) molecules (Gubin et al., 2014; Tumeh et al., 2014). Viruses have evolved a multitude of mechanisms to decrease MHC-I expression in infected cells to evade T cell surveillance and, similarly, reduced expression of MHC-I in cancer cells

Significance

Here we show that cancer cells co-opt an evolutionarily conserved, lineage-specific function of PRC2 to silence the MHC-I antigen processing and presentation pathway and evade immune surveillance. Importantly, PRC2 mediated silencing of MHC-I APP genes is reversible following pharmacological inhibition of EED or EZH2 and EZH1, leading to re-establishment of effective T cell-mediated anti-tumor immunity and providing a rationale for combining PRC2 inhibitors with immunotherapy to treat these aggressive MHC-I-deficient malignancies. Our findings suggest that resistance to cancer immunotherapies may occur not only through genomic mutations that inactivate the MHC-I APP but also through non-genomic mechanisms that exploit the activity of repressive chromatin complexes such as PRC2.

© 2019 The Authors. Published by Elsevier Inc.
This is an open access article under the CC BY license (http://creativecommons.org/licenses/by/4.0/).

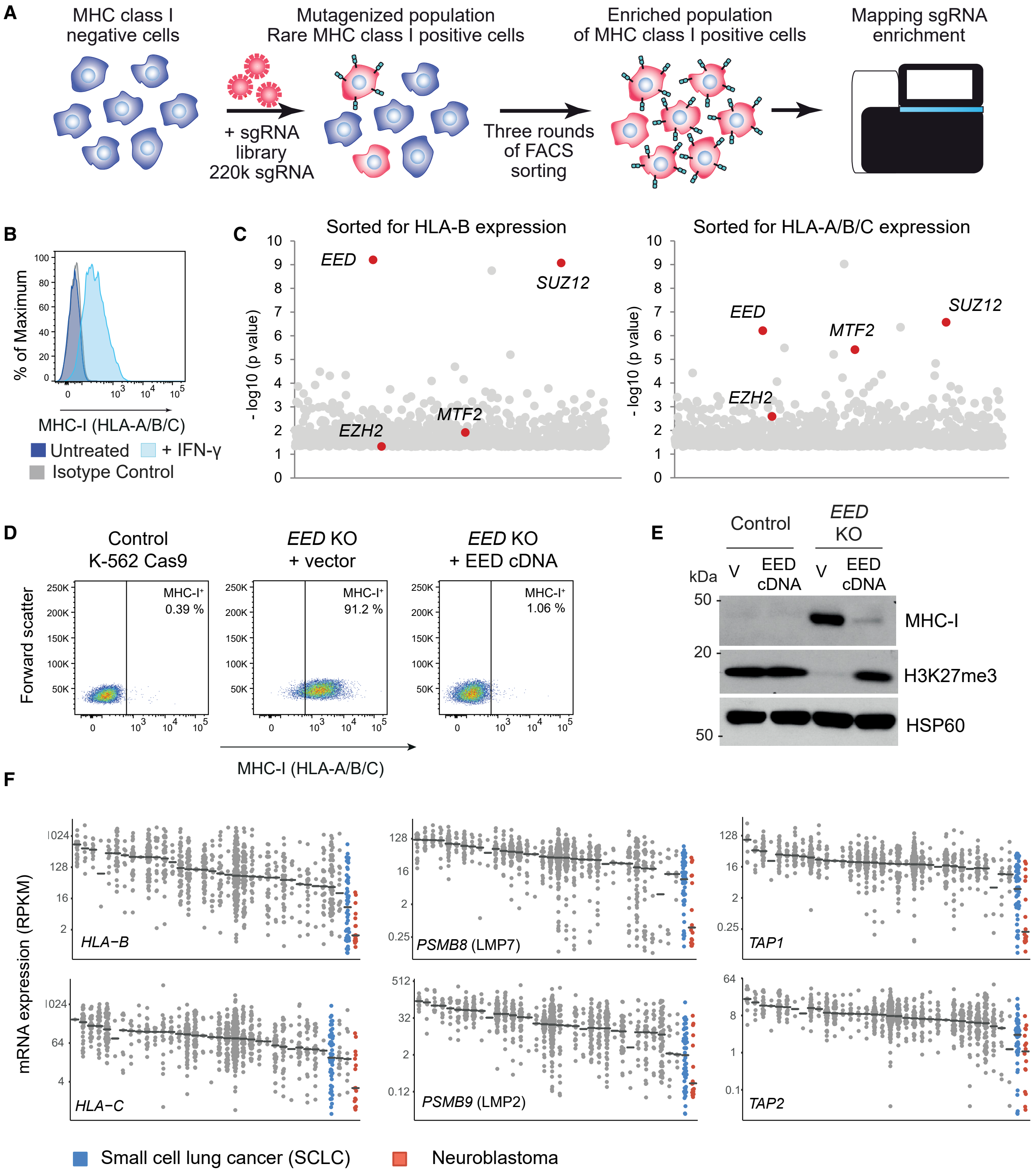

Figure 1. A Whole Genome CRISPR Screen Identifies a Critical Role for PRC2 in Silencing MHC-I Expression in Cancer Cells

(A) CRISPR screen. K-562 cells were mutagenized by infection with a pooled lentiviral library comprising 220,000 sgRNA and MHC-I high cells were enriched by three successive sorts using fluorescence-activated cell sorting.

(B) Cell surface MHC-I in K-562 cells following incubation ± IFN-γ 10 ng/mL for 24 h.

(C) Bubble plots show the top 1,000 enriched genes identified in the CRISPR screen. PRC2 genes indicated in red. p values calculated using the RSA algorithm (Konig et al., 2007).

(legend continued on next page)

provides a mechanism of resistance to CD8+ T cell-mediated immune attack. Recent major breakthroughs in cancer immunotherapy, particularly the capacity of antibodies targeting the T cell inhibitory receptors PD-1 and CTLA-4 to induce sustained disease remission in a proportion of cancer patients, has renewed interest in the molecular understanding of tumor antigen presentation. Consistent with a central role for cytotoxic T cells as the key effector cell activated in immune checkpoint inhibitor (ICI)-treated patients, mutations in genes encoding components of the MHC-I antigen processing pathway (APP) or the interferon-γ (IFN-γ) response pathway have emerged as a frequent cause of both primary and acquired resistance (Manguso et al., 2017; Zaretsky et al., 2016; Shin et al., 2017; Gao et al., 2016; Pan et al., 2018; Patel et al., 2017; Kearney et al., 2018). Conversely, augmenting the IFN-γ response and MHC-I expression enhances T cell-mediated anti-tumor immunity (Patel et al., 2017; Pan et al., 2018; Manguso et al., 2017). Together these studies emphasize the central importance of understanding the regulation of MHC-I antigen presentation in cancer cells to enable effective targeting with immunotherapy.

Transcriptional repression of MHC-I has been observed in a range of cancers, including neuroendocrine tumors such as N-myc-driven neuroblastoma, small-cell lung cancer (SCLC) and Merkel cell carcinoma (MCC), and has recently been identified as a mechanism of resistance to immunotherapy (Bernards et al., 1986; Restifo et al., 1993; Paulson et al., 2018). While MHC-I is universally expressed on normal nucleated cells, the quantitative expression of individual HLA genes is regulated in tissue- and cell-type-specific patterns to modulate immune function (Rene et al., 2016). During fetal development, selective transcriptional downregulation of HLA-A and HLA-B in the fetal extravillous trophoblast facilitates maternal tolerance of paternally encoded MHC-I alleles (Tilburgs and Strominger, 2013). Downregulation of MHC-I genes has also been observed in adult quiescent tissue-specific stem cells in the hair follicle and muscle (Agudo et al., 2018). These observations imply the existence of conserved physiological epigenetic regulatory mechanisms to suppress MHC-I expression that could provide a survival advantage to cancer cells under immune selection pressure. In contrast to mutations in MHC-I APP genes, transcriptional downregulation of MHC-I is potentially reversible and thus amenable to pharmacological manipulation. Here we aim to identify and characterize the molecular mechanisms underpinning MHC-I silencing in MHC-I low cancers.

RESULTS

A Whole Genome CRISPR/Cas9 Screen Identifies an Essential Role for Polycomb Repressive Complex 2 in Maintaining Transcriptional Repression of MHC-I

To identify conserved negative regulators of MHC-I expression, we performed a positive selection flow cytometry-based whole genome CRISPR/Cas9 screen in K-562 cells (Figure 1A), a well-characterized erythroleukemia line that lacks cell surface expression of all classical HLA alleles in the absence of IFN-γ stimulation (Figure 1B). Supporting a key role for epigenetic regulation of MHC-I expression in cancer cells, all the top candidate genes identified from cells with reactivated MHC-I detected with either an HLA-B-specific or a pan-HLA-A/B/C antibody encoded epigenetic regulators (Figures 1C and S1A; Table S1). Several of the targets are known to function together in multicomponent complexes, supporting the validity of these hits. The most significantly enriched of these were single guide RNAs (sgRNAs) targeting *EED* and *SUZ12*, encoding core components of polycomb repressive complex 2 (PRC2). *MTF2*, encoding a facultative subunit of PRC2.1 subcomplexes that has been implicated in PRC2 recruitment, was also among the top hits (Figure 1C; Table S1). PRC2 is a well-known epigenetic repressive complex that has a critical role in modulating gene expression during embryonic development, therefore this complex was prioritized for validation. Depletion of *EED* using independent sgRNAs restored MHC-I to the cell surface of K-562 cells (Figures 1D, S1B, and S1C), while established *EED* and *EZH2* knockout (KO) cells maintained MHC-I expression without substantial impairment in cell proliferation (Figure S1D). Importantly, reconstituting PRC2 function by expression of EED cDNA in *EED* KO cells was sufficient to restore H3K27me3 levels and reinstate silencing of actively transcribed MHC-I genes (Figures 1D and 1E). These findings demonstrate a critical role for PRC2 in both establishing and maintaining transcriptional repression of MHC-I genes.

Among all tumor types represented in the Cancer Cell Line Encyclopedia (Barretina et al., 2012), SCLC and neuroblastoma exhibit the lowest expression of multiple MHC-I APP genes, implying broad repression of the MHC-I APP in these neuroendocrine tumors (Figures 1F and S1E) (Restifo et al., 1993; Bernards et al., 1986). Notably, high expression of EZH2, the catalytic component of PRC2, is a typical feature of both SCLC and neuroblastoma (Figures S1F and S1G), and has been implicated in pathogenesis and therapy resistance (Gardner et al., 2017; Chen et al., 2018).

Supporting a conserved function for PRC2 in MHC-I silencing, *EED* KO restored cell surface expression of MHC-I in human SCLC and neuroblastoma cells (Figures S2A and S2B). Trimethylation of histone H3 on lysine 27 (H3K27me3), the hallmark of genes repressed by PRC2, is catalyzed by the lysine methyltransferase EZH2. Several potent and highly selective S-adenosyl-methionine (SAM)-competitive inhibitors of EZH2 methyltransferase activity have been developed and are in clinical trials in a range of malignancies. Treatment with these inhibitors substantially depleted H3K27me3 levels concomitant with transcriptional induction of MHC-I genes (Figures 2A and 2B). Importantly, pharmacological inhibition of EZH2 restored cell surface MHC-I in K-562 and cell lines representative of neuroblastoma, SCLC, and MCC, a neuroendocrine cancer recently

(D and E) *EED* KO K-562 cells were transduced with a lentiviral vector encoding either EED cDNA or GFP (vector, V) and analyzed by flow cytometry (D) and immunoblot (E).

(F) mRNA expression (reads per kilobase of transcript per million mapped reads) of MHC-I genes in 920 cancer cell lines in the Cancer Cell Line Encyclopedia. Each dot represents an individual cancer cell line, clustered by tumor type (log_2 scale, black line indicates median).

See also Figure S1 and Table S1.

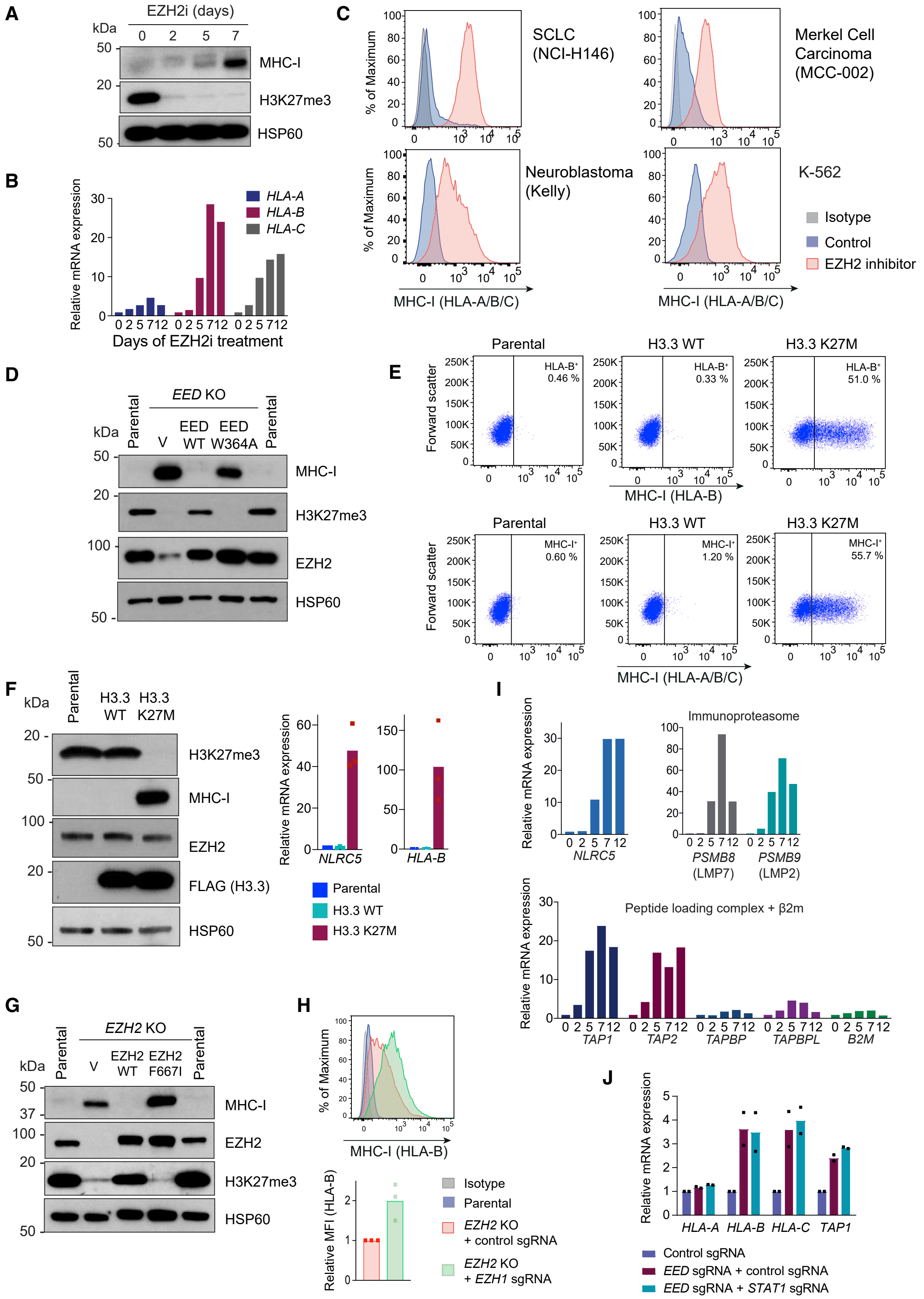

Figure 2. PRC2 Maintains Coordinated Silencing of Antigen Processing Genes in MHC-I-Deficient Cancers

(A and B) K-562 cells incubated with 3 μM EPZ-011989 (EZH2i) for the indicated times were analyzed by immunoblot (A) and qRT-PCR (B).

(C) Cell surface MHC-I in EZH2i-treated cells (GSK-503 5 μM in NCI-H146 and EPZ-011989 3 μM in Kelly, MCC-002, and K-562) after 10 days of treatment.

(legend continued on next page)

shown to escape from immunotherapy through transcriptional downregulation of MHC-I genes (Figure 2C) (Paulson et al., 2018).

The enzymatic function of EZH2 is markedly potentiated by the WD40 repeats of EED, which form an aromatic cage that binds to H3K27me3 to allosterically activate EZH2 and perpetuate H3K27me3 deposition (Margueron et al., 2009; Ueda et al., 2012). Genetic disruption of the EED aromatic cage impaired the ability of PRC2 to reinstate transcriptional repression of MHC-I, arguing that H3K27 is the critical target of EZH2 methyltransferase activity for this process (Figures 2D and S2C). To test this more directly, we took advantage of an H3 variant oncohistone with a lysine-27 to methionine mutation (H3.3 K27M), which is a major driver of pediatric diffuse intrinsic pontine glioma and acts in a dominant negative manner to inhibit allosteric activation of PRC2 and spreading of the H3K27me3 repressive mark (Stafford et al., 2018; Harutyunyan et al., 2019). Expression of H3.3 K27M in K-562 cells potently induced MHC-I expression (Figures 2E and 2F), demonstrating a pivotal role for the establishment of H3K27me3 repressive chromatin domains to maintain MHC-I silencing. In keeping with this, mutation of a critical catalytic residue in the EZH2 SET domain (EZH2 F667I) abrogated its methyltransferase activity and its ability to restore MHC-I repression (Figure 2G). Interestingly, we noted that expression of the EZH2 F667I mutant in *EZH2* KO cells exerted a dominant negative effect, further increasing MHC-I levels and implying the presence of residual PRC2 activity in the absence of EZH2. Consistent with this, loss of EED more potently induced MHC-I than loss of EZH2, accounting for the lesser enrichment of sgRNAs targeting *EZH2* compared with those targeting *EED* in the CRISPR screen (Figure 1C). It is well established that the EZH2 paralog, EZH1, can also associate with EED and SUZ12 to form functional PRC2 complexes. Although EZH1-containing PRC2 complexes have less efficient lysine methyltransferase activity than EZH2-containing complexes, they may compensate for the loss of EZH2 in specific cellular contexts (Wassef and Margueron, 2017). Deletion of *EZH1* in an *EZH2* null clone induced additional MHC-I upregulation, identifying a role for EZH1-mediated PRC2 activity in maintaining MHC-I repression in the absence of EZH2 (Figure 2H). While most currently available SAM-competitive and allosteric PRC2 inhibitors selectively target EZH2 with lesser potency for inhibition of EZH1 activity (Lee et al., 2018), our data suggest that inhibitors that effectively target both EZH2 and EZH1 catalytic activity may more effectively induce MHC-I expression in MHC-I low cancers.

Disruption of PRC2 Function Induces Expression of Multiple Components of the MHC-I APP Independently of STAT1 Signaling

MHC-I antigen processing is orchestrated by a multistep pathway that involves the coordinated regulation of multiple components. Across a range of tumor types we find that PRC2 silences critical genes essential for MHC-I antigen processing, leading to upregulation of multiple MHC-I APP genes following PRC2 inhibition, including those encoding an MHC-I transactivator (*NLRC5*), immunoproteasome components (*PSMB8* and *PSMB9*), peptide transporters associated with antigen processing (*TAP1* and *TAP2*), and MHC-I heavy chains (*HLA-B* and *HLA-C*) (Figures 2I and S2D). Recent studies have shown that inhibition of other epigenetic repressors such as DNA methyltransferases (DNMTs) and KDM1A (LSD1) augment MHC-I expression in cancer cells via derepression of endogenous retroviruses, which activates type I IFN signaling (Chiappinelli et al., 2015; Roulois et al., 2015; Sheng et al., 2018). STAT1 is an essential mediator of the transcriptional response to types I and II IFNs (Durbin et al., 1996) and, as expected, disruption of *STAT1* abolished IFN-γ-induced MHC-I upregulation (Figure S2E). However, in contrast to the findings with KDM1A and DNMT inhibitors (Sheng et al., 2018; Chiappinelli et al., 2015; Roulois et al., 2015), initiation and maintenance of MHC-I transcription following disruption of PRC2 function was independent of STAT1, suggesting that this was not solely a consequence of enhanced IFN signaling (Figures 2J and S2F). Consistent with this, phosphorylated STAT1 was not detected following EZH2 inhibition alone (Figure S2G). Collectively our findings imply a conserved role for the PRC2 complex in the direct repression of the multicomponent MHC-I APP and demonstrate that inhibition of PRC2 function is sufficient to restore cell surface MHC-I expression.

PRC2 Restricts Transcriptional Induction of MHC-I in Response to Cytokine Stimulation

While PRC2 inhibition alone increased MHC-I APP gene expression in multiple different MHC-I low tumor types (Figure 2C), in the absence of additional cytokine stimulation the degree of induction of cell surface MHC-I was variable in individual cancer cell lines. It is well established that IFN-γ-induced activation of *STAT1* and *IRF1* augments MHC-I antigen presentation and modulates the repertoire of peptides presented via induction of the immunoproteasome. Consistent with the notion that PRC2 inhibition can potentiate the transcriptional response to IFN-γ (Zingg et al., 2017; Peng et al., 2015; Abou El et al., 2015), genetic disruption of PRC2 dramatically augmented IFN-γ-induced MHC-I expression (Figure S3A). Expression of H3.3 K27M had similar effects to PRC2 disruption suggesting that the enhanced IFN-γ-induced MHC-I expression is a direct consequence of H3K27me3 loss (Figure 3A), rather than targeting of non-histone substrates (Ardehali et al., 2017). These effects were conserved in multiple MHC-I low cancers and pharmacological inhibition of PRC2 using either SAM-competitive EZH2 inhibitors (EZH2i) or

(D) Immunoblot in *EED* KO K-562 cells transduced with lentiviral vectors encoding WT EED, EED W364A mutant, or GFP control (V).

(E and F) Flow cytometry (E) and immunoblot and qRT-PCR (F) in K-562 cells transduced with lentiviral vectors encoding H3.3 WT or K27M.

(G) Immunoblot in *EZH2* KO K-562 cells transduced with lentiviral vectors encoding WT EZH2, EZH2 F667I, or GFP control (V).

(H) Cell surface MHC-I in *EZH2* KO K-562 cells transduced with either control sgRNA or two *EZH1*-specific sgRNAs.

(I) qRT-PCR analysis in K-562 cells incubated with 3 μM EPZ-011989 for the indicated times (days). Bars indicate the mean of technical triplicates from a representative experiment.

(J) qRT-PCR in K-562 Cas9 cells expressing control or *STAT1* sgRNA, 7 days following transduction with sgRNA targeting *EED*.

For (F), (H), and (J), points indicate mean fold change in expression from individual experiments and bars show mean fold change across experiments. See also Figure S2.

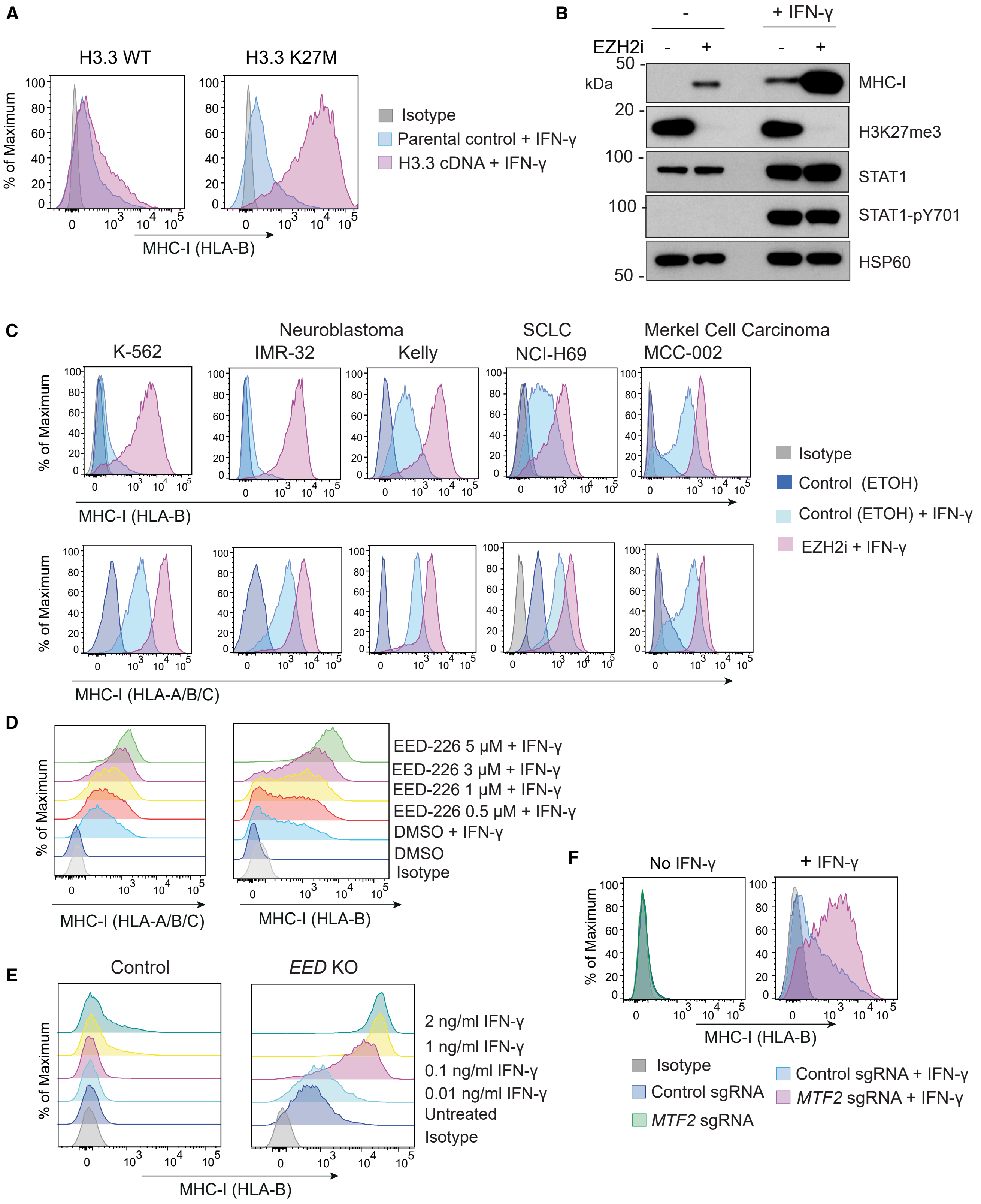

Figure 3. PRC2 Restricts the Interferon-Induced Activation of MHC-I APP Genes in Multiple MHC-I Low Tumor Types

(A) Cell surface MHC-I in K-562 cells expressing H3.3 WT or H3.3 K27M treated with IFN-γ 10 ng/mL for 24 h.

(B) Immunoblot in neuroblastoma (Kelly) cells treated with EPZ-011989 3 μM for 10 days ± IFN-γ 10 ng/mL for 24 h.

(legend continued on next page)

EED-226, an inhibitor that blocks H3K27me3 binding and allosteric activation of EZH2, markedly amplified IFN-γ-induced MHC-I upregulation (Figures 3B–3D, S3B, and S3C) (Qi et al., 2017) and reduced the threshold concentration of IFN-γ required to induce MHC-I gene expression (Figures 3E and S3D). Together these data demonstrate that PRC2 not only results in baseline repression of MHC-I genes, but also restricts transcriptional induction of these genes in response to cytokine stimulation.

Core PRC2 members EED, SUZ12, and EZH1/EZH2 can form various subcomplexes defined by their association with specific accessory proteins. Depletion of *MTF2*, which was identified as a hit in the CRISPR screen and encodes a component of PRC2.1 complexes, also augmented IFN-γ-induced MHC-I expression (Figure 3F). MTF2 binds to both non-methylated CpG motifs and H3K36me3 (Li et al., 2017), and has been proposed to modulate PRC2 recruitment to specific loci in embryonic stem cells (Holoch and Margueron, 2017). Consistent with an epistatic role for MTF2 in maintaining MHC-I repression via modulation of PRC2 function, loss of MTF2 had no additional effect on MHC-I in *EED* KO cells (Figure S3E).

PRC2-Mediated Silencing of MHC-I Drives Resistance to T Cell Killing in SCLC

SCLC is a highly aggressive neuroendocrine carcinoma that is strongly linked with smoking and has an extremely poor prognosis. Notably, the rates of response to ICIs have been lower than seen in other cancers with a similarly high mutational burden (Alexandrov et al., 2013; Sabari et al., 2017). We found that expression of EZH2 is negatively correlated with expression of both MHC-I genes and markers of $CD8^+$ T cells in primary SCLC samples (Figure S4). To explore the effects of PRC2 inhibition on T cell responses to SCLC, we established primary lung tumor cell lines from a genetically engineered mouse model (GEMM) driven by biallelic loss of *Trp53* and *Rb1.* Biallelic inactivation of *TP53* and *RB1* is a hallmark of human SCLC, and this GEMM closely mimics human SCLC (Sutherland et al., 2011; Meuwissen et al., 2003; George et al., 2015). Like human neuroendocrine tumors, mouse SCLC (mSCLC) lacked cell surface MHC-I and exhibited low expression of multiple MHC-I APP genes (Figures 4A and S5A), which were induced following genetic disruption or pharmacological inhibition of PRC2 (Figures 4B, 4C, S5B, and S5C). As in K-562 cells, re-expression of wild type (WT), but not catalytically inactive (F667I) EZH2 reinstated MHC-I repression in *Ezh2* KO mSCLC (Figure 4C). Like human neuroendocrine tumor lines (Figure 3D), these early-passage mSCLC lines showed variable MHC-I induction in response to IFN-γ stimulation (Figure 4A), while pretreating cells with PRC2 inhibitors markedly potentiated IFN-γ-induced MHC-I upregulation (Figures 4D, S5D, and S5E).

To explore the functional consequences of low MHC-I expression in these mSCLC cells, we pulsed cells with ovalbumin (OVA) peptide (SIINFEKL), which binds to cell surface MHC-I (H-2Kb). Cells were washed to remove unbound peptide, before co-culture with TCR transgenic OT-I T cells that specifically recognize H-2Kb bound OVA peptide (Figure 4E). mSCLC cells were highly resistant to antigen-specific T cell killing and failed to induce T cell cytokine production (Figures 4F and 4G). Importantly, this resistance was dramatically reversed by pretreating SCLC with both EZH2i and IFN-γ to maximally induce MHC-I expression before peptide pulsing, which led to effective T cell activation (Figures 4F and 4G). These data clearly establish the functional importance of MHC-I cell surface expression in this model of SCLC.

We next investigated whether PRC2 inhibition restores intracellular MHC-I antigen processing by engineering mSCLC cells to express the full-length foreign protein chicken OVA (Figure 5A). Intracellular processing of OVA generates the SIINFEKL peptide, which is loaded onto MHC-I (H-2Kb) and specifically recognized by the OT-I T cell receptor. Treatment of mSCLC-OVA cells with EZH2i enabled IFN-γ-inducible expression of OVA peptide bound to MHC-I (SIINFEKL:Kb), which was not detected in IFN-γ-treated control cells (Figure 5B). In contrast to the peptide pulsing assay (Figures 4E–4G), pretreatment with EZH2i alone was sufficient to prime mSCLC-OVA cells to induce activation of co-cultured OT-I T cells leading to pro-inflammatory cytokine production (Figure 5C) and effective tumor cell killing over 96 h (Figure 5D), establishing the functional importance of reversing PRC2-mediated repression of the MHC-I APP. While pulsed OVA peptide binds only to MHC-I already present at the cell surface before adding T cells (Figures 4E–4G), co-culture of EZH2i pretreated, but not control, OVA-expressing cells with OT-I T cells induced MHC-I upregulation via a feedforward loop of T cell activation (Figure 5E). Here, PRC2 inhibition alleviates transcriptional repression of MHC-I leading initially to low level cell surface expression, which primes SCLC to induce $CD8^+$ T cell activation and cytokine production. These cytokines further enhance MHC-I antigen processing, resulting in efficient antigen-specific T cell activation and killing.

PRC2 Facilitates Transmission of MHC-I-Silenced Cancers to Non-histocompatible Recipients

To explore whether MHC-I downregulation evokes resistance to T cell killing of SCLC cells *in vivo*, we subcutaneously transplanted C57Bl/6 (H-2b haplotype)-derived mSCLC cells into BALB/c (H-2d haplotype) mice. Cells with foreign MHC are expected to be eliminated by an allogeneic T cell response; however, these tumors were transmissible to BALB/c mice (Figure 6A) and lacked cell surface MHC-I with few tumor infiltrating $CD8^+$ T cells in either the allogeneic or syngeneic setting (Figures S5F and S5G). The ability of SCLC cells to engraft and grow in non-histocompatible recipients resembles that of rare transmissible cancers such as the Tasmanian devil facial tumor (DFT), which exemplifies immune evasion by cancer cells. Devils exhibit modest diversity at MHC-I loci that should be sufficient to trigger a T cell response; however, these tumors escape rejection by the

(C and D) Cell surface MHC-I following incubation with IFN-γ 10 ng/mL for 24 h in indicated cell lines pretreated with EPZ-011989 3 μM (C) and K-562 cells pretreated with EED-226 (D). Analysis after 10 days of inhibitor treatment.

(E) Cell surface MHC-I in *EED* KO or parental control K-562 cells incubated with the indicated concentrations of IFN-γ for 24 h.

(F) Cell surface MHC-I in K-562 Cas9 cells expressing control sgRNA or two *MTF2*-specific sgRNAs incubated ± IFN-γ 10 ng/mL for 24 h.

See also Figure S3.

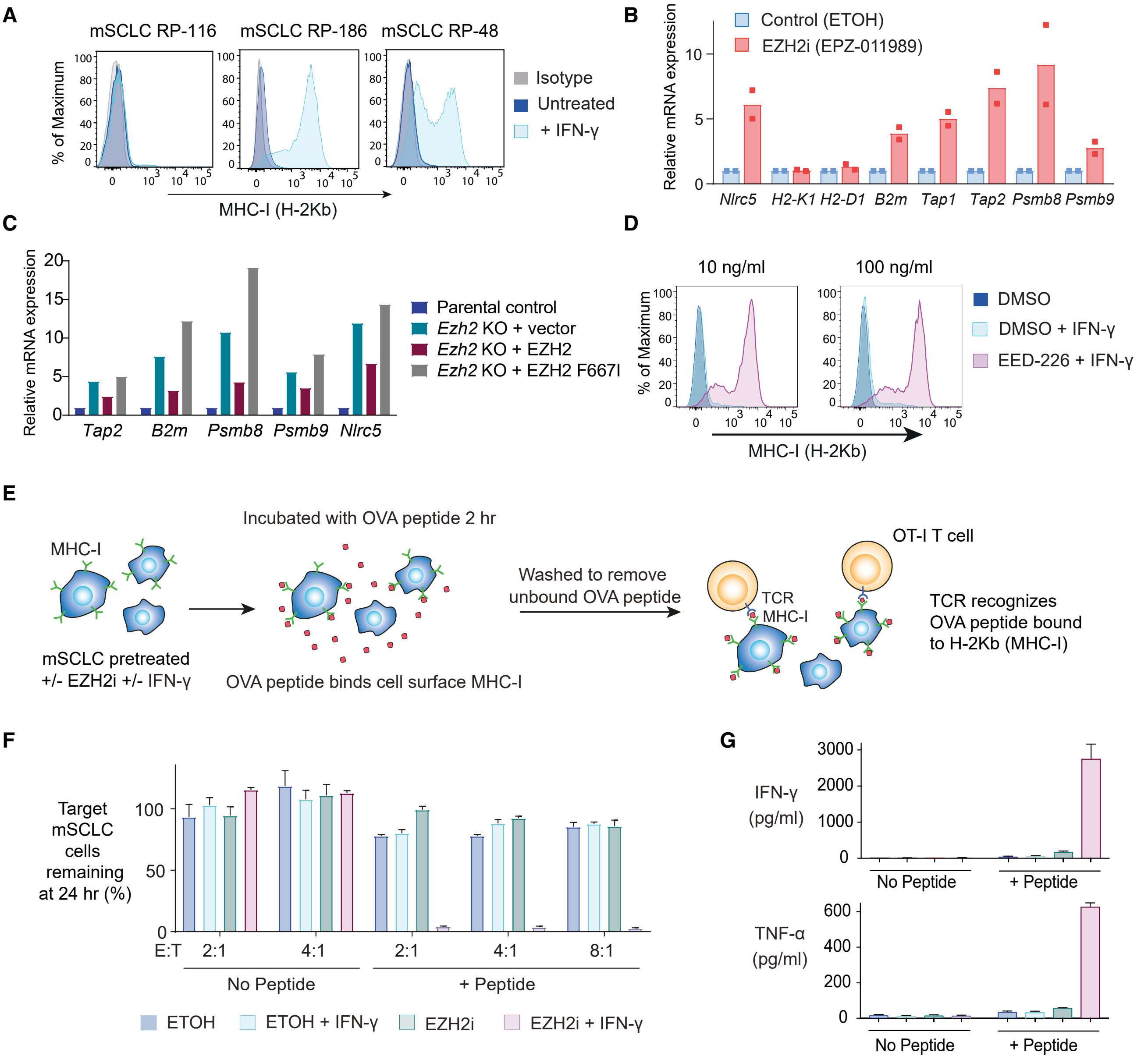

Figure 4. PRC2-Mediated Silencing of MHC-I Antigen Presentation in a Mouse Model of SCLC Drives Resistance to T Cell Killing

(A) Cell surface MHC-I in mSCLC cell lines following incubation ± IFN-γ 10 ng/mL for 24 h.

(B) qRT-PCR for MHC-I genes in mSCLC RP-116 cells treated with 3 μM EPZ-011989 or ethanol control for 10 days. Bars depict mean fold change in expression from independent experiments and points indicate the mean of technical triplicates from individual experiments.

(C) qRT-PCR in *Ezh2* KO mSCLC RP-116 transduced with a lentiviral vector encoding WT EZH2, EZH2 F667I, or GFP vector control. Bars show mean fold change in expression from a representative experiment.

(D) Cell surface MHC-I in RP-116 cells treated with IFN-γ for 24 h following pretreatment ± EED-226 3 μM for 10 days.

(E) Peptide pulsing assay. mSCLC RP-116 cells were pretreated as indicated with 3 μM EPZ-011989 or ethanol control for 10 days ± IFN-γ 10 ng/mL for 24 h before pulsing with OVA peptide.

(F) Percent remaining live target tumor cells following 24 h incubation with OT-I T cells at the indicated effector:target (E:T) ratios.

(G) Cytometric bead array (CBA) assay for T cell effector cytokines following 24 h co-culture with mSCLC cells pretreated as indicated. (F and G) Mean and SEM from a representative experiment performed in triplicate. Each experiment was performed independently three times with consistent results.

See also Figures S4 and S5.

devil immune system (Siddle, 2017). Interestingly, DFT1 tumors lack cell surface MHC-I due to low expression of MHC-I APP genes (Siddle et al., 2013). Like neuroblastoma, DFT is of neural crest origin (Murchison et al., 2010) raising the possibility that similar mechanisms of MHC-I silencing may operate in these tumors. Supporting an evolutionarily conserved role for PRC2 in the repression of MHC-I antigen presentation, PRC2 inhibition induced expression of multiple MHC-I genes in DFT cells

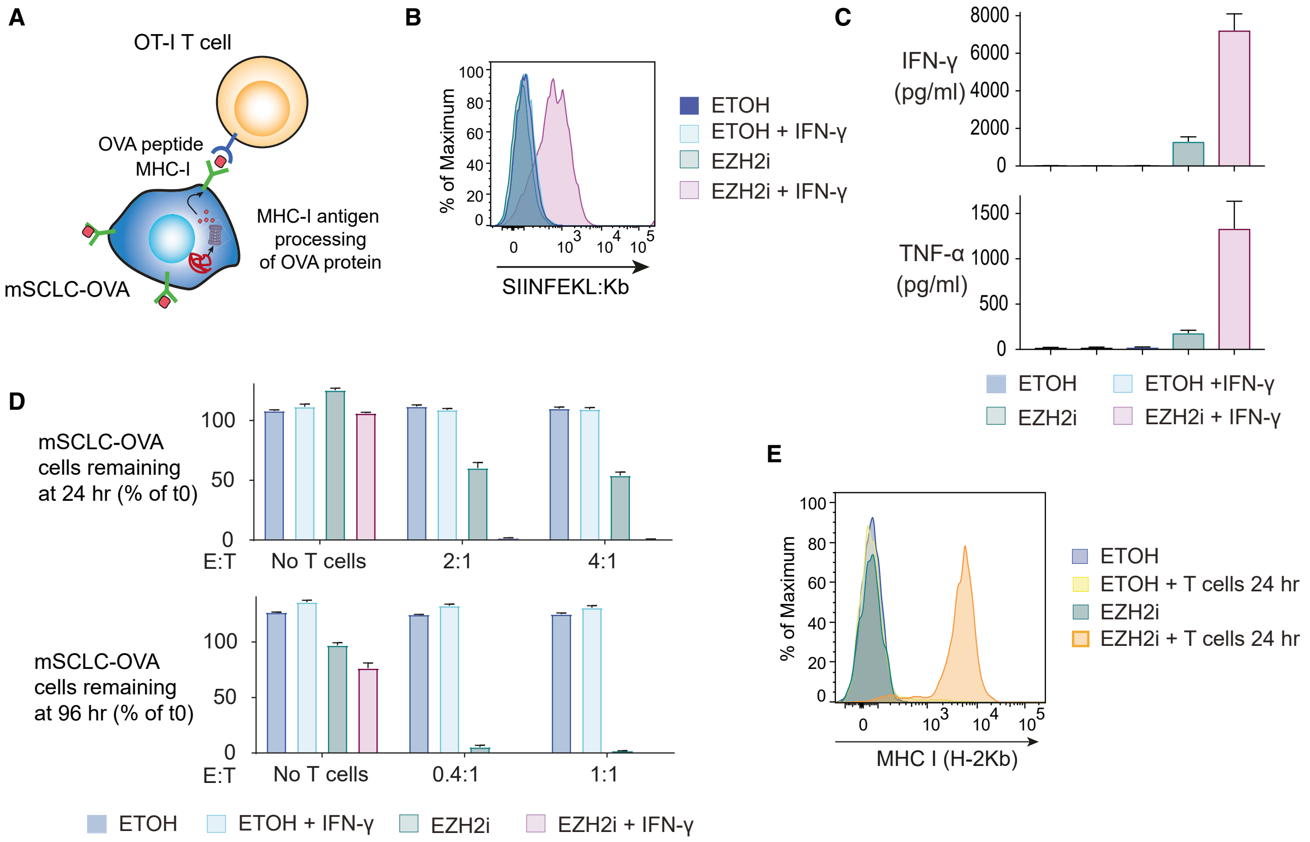

Figure 5. Pharmacological Inhibition of EZH2 Overcomes Resistance to T Cell Killing in SCLC

(A) Schematic showing mSCLC-OVA co-culture assay. Stable expression of full-length OVA in mSCLC cells allows functional evaluation of the intracellular MHC-I antigen-processing pathway. Processed OVA peptide bound to H2-Kb is presented at the tumor cell surface and recognized by co-cultured antigen-specific OT-I T cells.

(B) Cell surface SIINFEKL:Kb (MHC-I bound OVA peptide) in OVA-expressing mSCLC RP-116 cells pretreated as indicated with 3 μM EPZ-011989 for 10 days ± IFN-γ 10 ng/mL for 24 h.

(C) CBA assay for T cell effector cytokines following 24 h co-culture with mSCLC-OVA cells pretreated as indicated.

(D) Percent remaining live mSCLC-OVA cells following incubation with OT-I T cells at the indicated effector:target (E:T) ratios.

(E) Cell surface MHC-I levels of mSCLC-OVA cells pretreated with EZH2 inhibitor ±24 h co-culture with OT-I T cells. (C and D) Mean and SEM from a representative experiment performed in triplicate. Each experiment was performed three times with consistent results.

(Figure 6B) and potentiated MHC-I induction following IFN-γ stimulation (Figure S6A). To evaluate the importance of PRC2-mediated MHC-I repression for evasion of the potent allogeneic $CD8^+$ T cell-mediated anti-tumor response, we performed allogeneic transplants with *Ezh2* KO mSCLC cells. Loss of EZH2 led to universal rejection of mSCLC tumors in BALB/c mice (Figures 6C, 6D), while the growth of EZH2-deficient mSCLC tumors was not impaired either *in vitro* or following injection into immunodeficient non-obese diabetic-severe combined immunodeficiency IL2Rγ^{null} mice that lack functional T, B, and natural killer cells (Figures S6B and S6C). These findings confirm a critical role for tumor-intrinsic PRC2 function in promoting immune evasion *in vivo*.

To illustrate the clinical importance of our findings, we evaluated MHC-I expression in a patient who presented with an epidermal growth factor receptor (EGFR) mutant lung adenocarcinoma that subsequently transformed to SCLC following treatment with the EGFR inhibitor Erlotinib (Figure 6E). Neuroendocrine transformation of both lung and prostate adenocarcinoma is increasingly been recognized as a mechanism of escape from targeted therapies (Oser et al., 2015; Aggarwal et al., 2018). In this patient, while being clonally related to the adenocarcinoma and not showing any coding mutations in MHC-I genes, the transformed SCLC exhibited substantially reduced expression of components of the MHC-I APP, including the MHC-I heavy chain, β2-microglobulin, and the immunoproteasome component LMP7 (encoded by *PSMB8*) (Figures 6E and S6D). Consistent with loss of MHC-I antigen presentation, the tumor failed to respond to anti-PD-1 immunotherapy. Similar findings were noted in two additional patients with neuroendocrine transformation of EGFR mutant lung adenocarcinoma following treatment with EGFR inhibitors, both of whom showed reduced MHC-I staining in the transformed SCLC (Figure S6D). Together these data provide an example of how neuroendocrine transformation, arising as a mechanism of resistance to targeted therapies, may simultaneously confer immune privilege to a tumor through loss of MHC-I antigen presentation.

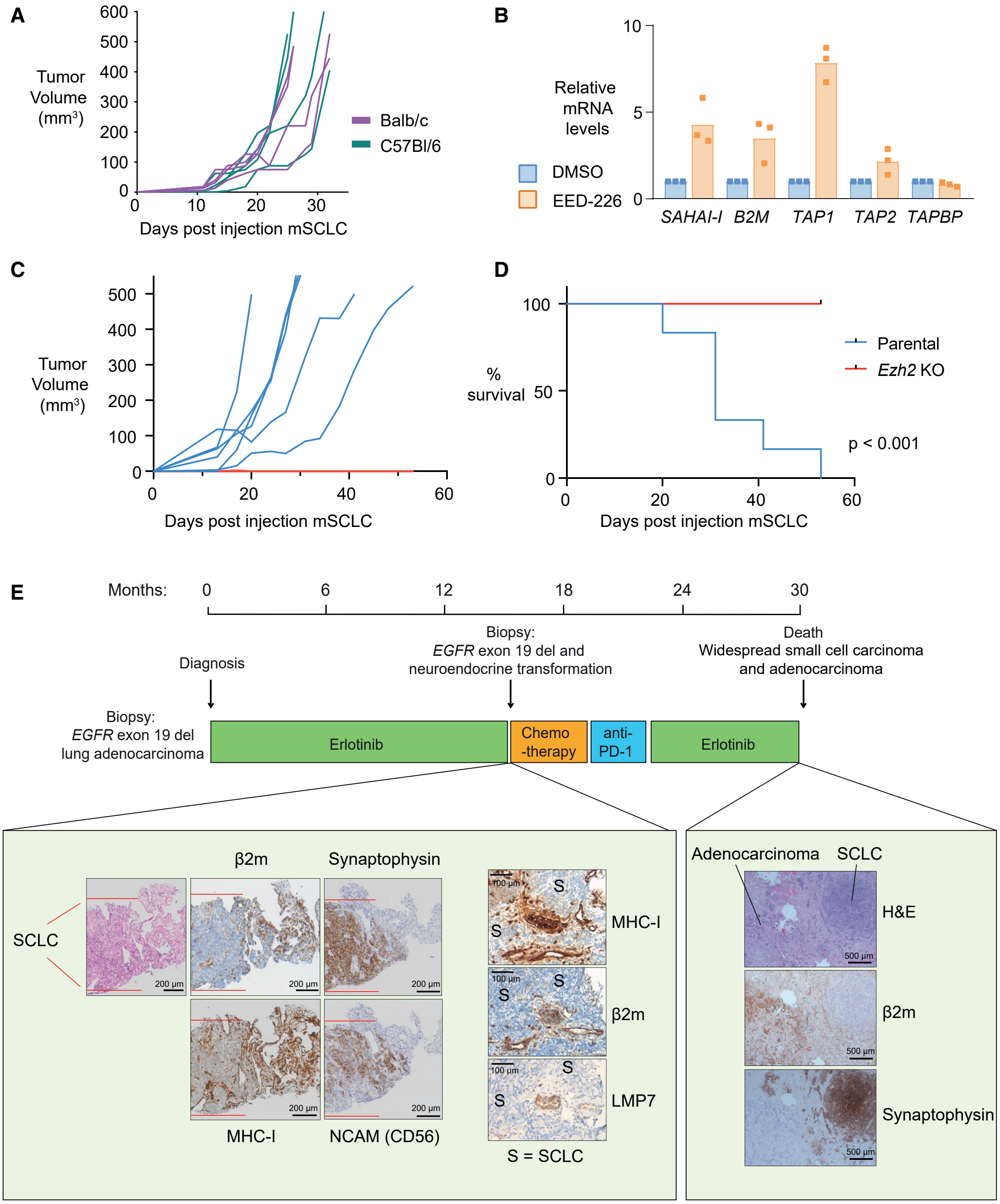

Figure 6. Silencing of MHC-I Antigen Processing Is a Conserved Function of PRC2 that Facilitates Immune Evasion in Transmissible Cancers

(A) mSCLC RP-116 tumor growth following subcutaneous transplant into syngeneic C57BL/6 mice (100,000 cells) or allogeneic BALB/c mice (500,000 cells). Endpoint at a tumor volume of 500 mm^3.

(B) qRT-PCR analysis of MHC-I gene expression in DFT1 cells C5065 following treatment with 3 μM EED-226 for 10 days. Bars depict mean fold change in expression from independent experiments and points indicate the mean of technical triplicates from individual experiments. *SAHAI-1* encodes Tasmanian devil MHC-I.

(C and D) Tumor growth in (C) and survival of (D) BALB/c mice subcutaneously injected with 10^6 *Ezh2* KO or parental mSCLC RP-116 cells. Endpoint at a tumor volume of 500 mm^3. Six mice per group. p value calculated using Mantel-Cox test.

(E) Progression biopsy (left) and postmortem histology (right) in patient 1. Immunohistochemistry for neuroendocrine markers (synaptophysin and NCAM) and MHC-I APP components (MHC-I, β2m, and LMP7). Area between the red lines in biopsy contains SCLC.

See also Figures S4 and S6.

H3K27me3 Modification of MHC-I Genes

Although PRC2 complex components are ubiquitously expressed, the genes repressed by PRC2 are highly cell-type specific (Comet et al., 2016; Wassef and Margueron, 2017), highlighting the importance of understanding the molecular basis of PRC2 function in different cellular contexts. Our observations that PRC2 inhibition potentiates IFN-γ-induced MHC-I expression prompted us to couple H3K27me3 chromatin immunoprecipitation sequencing with RNA sequencing (RNA-seq) analyses following genetic or pharmacological inhibition of PRC2 in the presence and absence of IFN-γ. These experiments were conducted in human and mouse cells to gain further insights into the conserved specificity of PRC2 function in cancers with low MHC-I. While most H3K27me3-modified PRC2-regulated genes were unaffected by exposure to IFN-γ (cluster 3; Figures 7A and S7A), in both cell types we identified clusters of genes that showed an augmented response to IFN-γ following disruption of PRC2 (clusters 1 and 2; Figures 7A and S7A). Consistent with the known lineage-specific function of PRC2, the defined set of PRC2-regulated IFN responsive genes in each cell type were largely distinct, with the notable exception of MHC-I APP genes (Figure S7B; Table S2). While PRC2 can both directly and indirectly modulate cytokine signaling (Zingg et al., 2017; Abou El et al., 2015; Canadas et al., 2018), our findings suggest that H3K27me3 modification of MHC-I APP genes plays a critical role in maintaining MHC-I repression in cancer cells. Supporting this, deletion of *EED* results in loss of H3K27me3 at MHC-I APP gene promoter regions and MHC-I derepression, which can be reinstated by WT EED but not an EED mutant that restores EZH2 levels but has impaired binding to H3K27me3 (Figures 7B and 2D).

Conserved Bivalent H3K4me3 and H3K27me3 Modification of MHC-I Genes in Multipotent Stem Cells and Cancer Cells

Critical genes that are poised for expression in a tissue-specific context are often concurrently marked by activating H3K4me3 and repressive H3K27me3 histone modifications, which maintain genes in a silent but "poised" state to allow either rapid transcriptional activation or progression to stable silencing (Bernstein et al., 2006; Voigt et al., 2013). Given the evidence that MHC-I expression is regulated during development and tissue-specific differentiation, we explored whether silenced MHC-I genes are marked by bivalent histone modifications. H3K27me3-modified genes that were derepressed following PRC2 disruption had higher average H3K4me3 levels at their promoters compared with other H3K27me3-modified genes for which expression did not significantly increase (Figure S7C; clusters 1–3 compared with other H3K27me3-modified genes). Indeed, a substantial proportion of the H3K27me3-modified genes responding to PRC2 inhibition and IFN-γ, including MHC-I APP genes, also possessed H3K4me3 at their promoters (Figures 7C, S7D, and S7E). The presence of bivalent H3K4me3 and H3K27me3 modifications at MHC-I APP gene promoters was observed in a range of human MHC-I-deficient cancers as well as mouse and Tasmanian devil tumors (Figures 7C and S7E). This conservation across different tumor types and species implied that bivalency could represent a physiological mechanism of MHC-I regulation. Both neuroblastoma and DFT1 arise from cells of the embryonic neural crest and share phenotypic features with SCLC and MCC (Murchison et al., 2010). Examination of MHC-I genes in neural crest cells revealed that these genes contain bivalent chromatin at their promoters and are transcriptionally repressed (Figures 7C and S7E) (Minoux et al., 2017). Similarly, while MHC-I genes are active in adult somatic tissues, we found that pluripotent tissues such as embryonic stem cells exhibit bivalency at MHC-I gene promoters, which is maintained during differentiation into neural progenitors but is resolved during mesenchymal differentiation (Figures 7D and 7E).

Disruption of PRC2 Leads to Derepression of NLRC5 and Enhanced IRF1 Binding at MHC-I Gene *cis*-Regulatory Regions

A number of transcription factors are known to bind MHC-I gene promoters and contribute to MHC-I expression (Rene et al., 2016). Among these, IRF-1, nuclear factor κB (NF-κB), and NLRC5 are key factors required for the transcriptional induction of MHC-I genes in response to cytokine stimulation (Kobayashi and van den Elsen, 2012). Interestingly, *NLRC5* was repressed by H3K27me3 in MHC-I low cancers and induced following PRC2 disruption (Figure 8A). *NLRC5* is an IFN-inducible gene encoding a transactivator that selectively binds MHC-I APP gene promoters in association with a multisubunit complex termed the MHC enhanceosome and plays a pivotal role in the regulation of MHC-I expression (Figure 8B) (Kobayashi and van den Elsen, 2012). To identify the transcription factors driving MHC-I expression following loss of PRC2, we used targeted CRISPR-mediated gene disruption in *EED*/*EZH2* KO cells. While IFN-γ-induced upregulation of MHC-I is largely dependent on RFX5/NLRC5 enhanceosome function (Figure 8C), deletion of either *NLRC5* or *RFX5* only partially attenuated MHC-I upregulation following *EED* KO or EZH2 inhibition, suggesting that MHC-I induction following loss of PRC2 is not driven through *NLRC5* derepression alone (Figures 8D and S8A). We also observed a partial requirement for IRF1, but not the NF-κB transcriptional activator p65 (RelA), for MHC-I upregulation following *EED* KO (Figures 8D, S8B, and S8C). Consistent with a role for IRF1 in driving MHC-I expression following PRC2 loss, IRF1 binding at MHC-I gene promoters increased following PRC2 inhibition (Figures 8E and S8D). Genome-wide, 72 of 104 genes showing increased IRF1 occupancy following PRC2 inhibition were modified by H3K27me3, implying that PRC2 restricts IRF1 binding at these genes (Figure S8E). Notably, although IFN-γ stimulation leads to a greater transcriptional induction of *IRF1* and *NLRC5* than *EED* KO alone, the relative expression of *HLA-B* is similar in the two contexts (Figure 8F). Together these findings are consistent with additional restriction of *HLA-B* expression in the presence of an intact PRC2 complex and suggest that loss of PRC2 activity and the consequent decrease in H3K27me3 at the MHC-I APP genes is the initiating process for gene expression. The loss of H3K27me3 provides a permissive chromatin environment that alleviates the constraints on cytokine stimulation and enables the subsequent binding of transcription factors that further potentiate the expression of MHC-I genes.

DISCUSSION

While cancers have previously been shown to corrupt the resolution of bivalently marked oncogenes and tumor suppressor

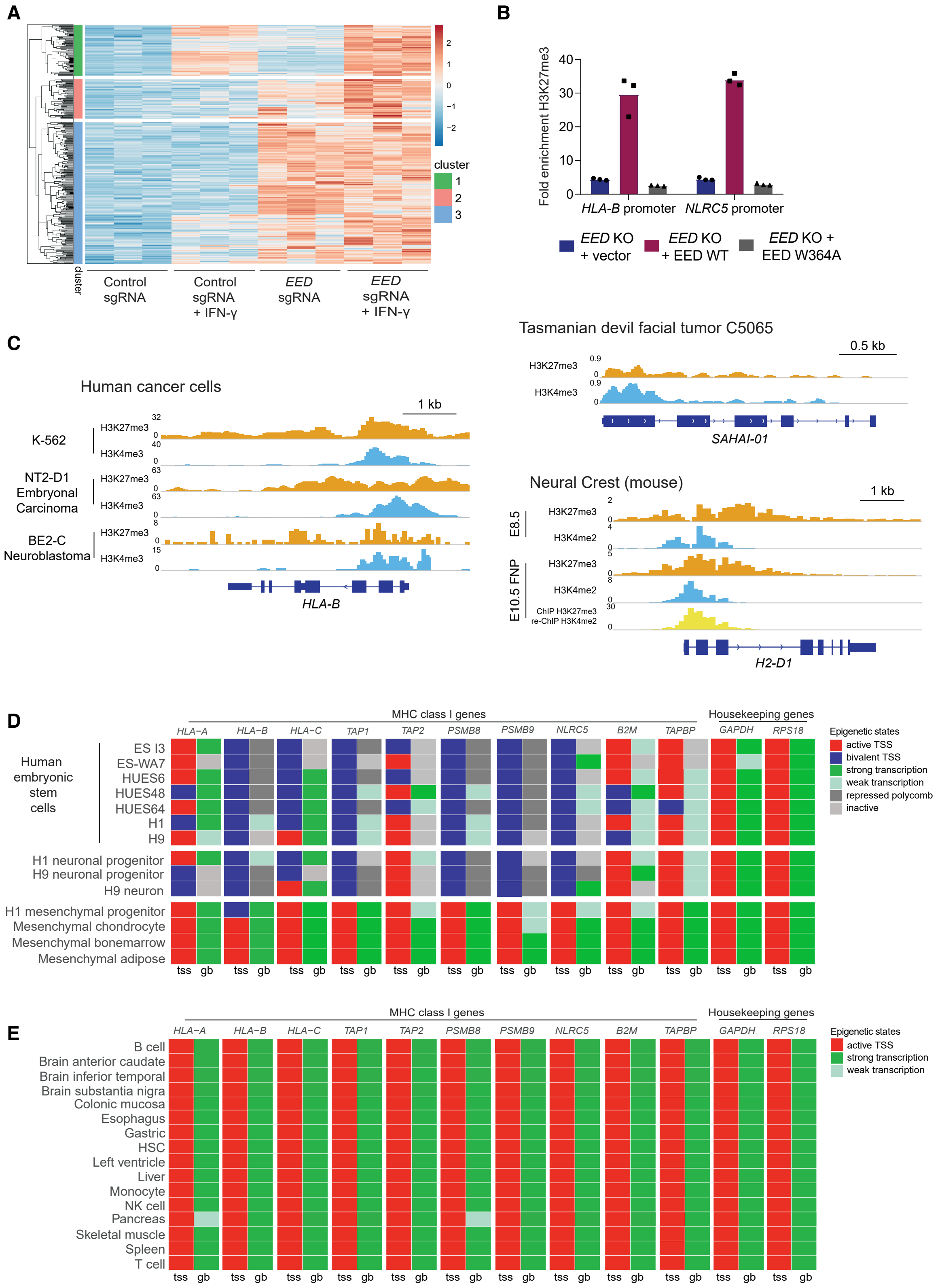

(legend on next page)

genes to impair differentiation pathways and promote survival, here we show how PRC2 function can be co-opted to maintain bivalency at a subset of genes to evade immune surveillance. The cell of origin appears to be central to this process and PRC2-mediated silencing of MHC-I in both embryonic and tissue-specific stem cell subsets likely evolved to protect these cells from inflammation-related pathology (Agudo et al., 2018). However, cancers that arise from these lineages can exploit the activity of PRC2 to silence MHC-I antigen presentation and gain immune privilege. Importantly, genetic disruption or pharmacological inhibition of PRC2 restores tumor MHC-I antigen presentation, allowing effective targeting by $CD8^+$ T cells.

Our findings demonstrate a critical role for the PRC2 complex in maintaining the coordinated silencing of critical components of the MHC-I APP, including the immunoproteasome and TAP peptide transporter as well as the MHC-I heavy chain genes. In addition to direct modification of MHC-I APP genes, PRC2 also targets *NLRC5*, derepression of which contributes to driving MHC-I gene expression following PRC2 inhibition. It is well recognized that only a proportion of PRC2 occupied and H3K27me3 marked genes become transcriptionally active following disruption of PRC2 function and loss of H3K27me3. The transcriptional induction of PRC2 target genes following PRC2 inhibition is largely determined by transcription factor expression and therefore is cell type and context specific (Wassef and Margueron, 2017). We observed variability in the degree to which PRC2 inhibition alone induced MHC-I gene expression in individual neuroendocrine tumor lines, which may in part reflect low basal expression of IRF1 and NF-κB components in these malignancies (van 't Veer et al., 1993). In contrast, PRC2 inhibition consistently augmented MHC-I gene expression following IFN-γ stimulation, which induces high levels of IRF1 and activated phosphorylated STAT1, highlighting the importance of PRC2 as a barrier to cytokine-mediated transcriptional activation of MHC-I APP genes in these cancers. While our data highlight a pivotal role for PRC2 in MHC-I APP regulation, our unbiased CRISPR screen also identified other candidates. Future work will provide insight into how these other chromatin regulators cooperate with PRC2 and/or nuance its function to regulate cell context dependent antigen presentation.

Here we have demonstrated a tumor-intrinsic function of PRC2 in the regulation of antigen presentation within tumors that arise from certain cell lineages. Our findings contribute to an emerging body of literature that ascribes a key immune modulatory function to PRC2. In addition to directly targeting MHC-I APP genes, EZH2 inhibition can enhance tumor immunogenicity through derepression of endogenous retroviruses, enhanced IFN signaling, and increased production of pro-inflammatory chemokines CXCL9 and CXCL10 (Canadas et al., 2018; Peng et al., 2015; Zingg et al., 2017; Wee et al., 2014). EZH2 also plays a pivotal role in modulating immune cell differentiation (Karantanos et al., 2016), and targeted EZH2 disruption in regulatory T cells has been shown to enhance anti-tumor immune responses in mouse tumor models (Wang et al., 2018; Goswami et al., 2018). In conjunction with these studies, our demonstration that tumor-intrinsic disruption of PRC2 leads to re-establishment of effective T cell-mediated anti-tumor immunity in MHC-I-deficient cancers provides a rationale for combining inhibitors of the PRC2 complex with immunotherapy to treat these aggressive malignancies.

MHC-I-deficient tumors such as neuroblastoma and SCLC are characterized by highly aggressive growth with early and widespread metastasis, features which epitomize failure to elicit an effective anti-tumor adaptive immune response. Importantly, SCLC and MCC possess potential foreign antigens, either neoantigens because of a high mutational burden or Merkel cell polyomavirus antigens. Silencing of MHC-I genes, likely coupled to the neuroendocrine differentiation program characteristic of these cancers, could therefore facilitate evasion of immune surveillance from an early stage in their development. Melanomas are immunogenic tumors that typically express high levels of MHC-I; however, melanocytes originate from the neural crest, and selective transcriptional downregulation of HLA-B has been reported in melanoma cell lines (Marincola et al., 1994), while melanoma dedifferentiation with expression of neural crest markers has been linked to acquired immunotherapy resistance (Landsberg et al., 2012; Mehta et al., 2018). Dedifferentiation or transformation of tumors is an emerging theme in the context of targeted therapy. As illustrated by our clinical cases, neuroendocrine transformation may provide an additional advantage to the tumor by coupling therapeutic resistance with suppression of antigen presentation. The molecular insights described here provide the rationale for future therapeutic strategies that incorporate PRC2 inhibition to negate this immune privilege. In summary, our findings provide a detailed molecular understanding of how MHC-I-deficient cancers co-opt an evolutionarily conserved, lineage-specific function of an epigenetic complex to evade immune surveillance. They also raise the prospect that resistance to cancer immunotherapies may occur not only through genomic mutations that inactivate the MHC-I APP but

Figure 7. An Evolutionarily Conserved Function of PRC2 Maintains Bivalency at MHC-I Gene Promoters in Embryonic Stem and Tissue-Specific Progenitor Cells and in MHC-I Low Cancers

(A) RNA-seq heatmap displaying H3K27me3-modified genes upregulated by >1.5 $\log_2$FC in K-562 7 days following transduction with *EED* sgRNA compared with control sgRNA. Cells were additionally pulsed ± IFN-γ 10 ng/mL for 24 h. Genes were clustered using Euclidean clustering, and clusters were separated using a K_{means} of 3. Red indicates higher expression and blue indicates lower expression.

(B) H3K27me3 chromatin immunoprecipitation (ChIP) qPCR at *HLA-B* and *NLRC5* promoters in *EED* KO K-562 transduced with control GFP vector, WT EED, or EED W364A. Fold enrichment was calculated relative to the signal at the *GAPDH* promoter (negative control). Bars show the mean of three technical replicates.

(C) Bivalent H3K27me3 and H3K4me3 modification of MHC-I gene promoters in human and Tasmanian devil cancer cells and mouse neural crest cells. ChIP sequencing (ChIP-seq) in K-562 and Tasmanian devil cells was performed in-house. Neural crest data are from GEO: GSE89435 (Minoux et al., 2017), BE2-C from GEO: GSE80151 (Zeid et al., 2018), and NT2-D1 from GEO: GSE31755 (ENCODE Project Consortium, 2012). y axes indicate reads per million (rpm).

(D and E) Chromatin state discovery tracks in selected human embryonic stem and progenitor cells (D) and normal adult human tissues (E) generated using ChromHMM software integrating H3K27me3, H3K4me3, H3K27ac, and H3K36me3 ChIP-seq data from NIH Roadmap Epigenomics Mapping Consortium (Kundaje et al., 2015). Generated chromatin annotation states for the TSS (tss) and gene body (gb) of the indicated genes are shown.

See also Figure S7 and Table S2.

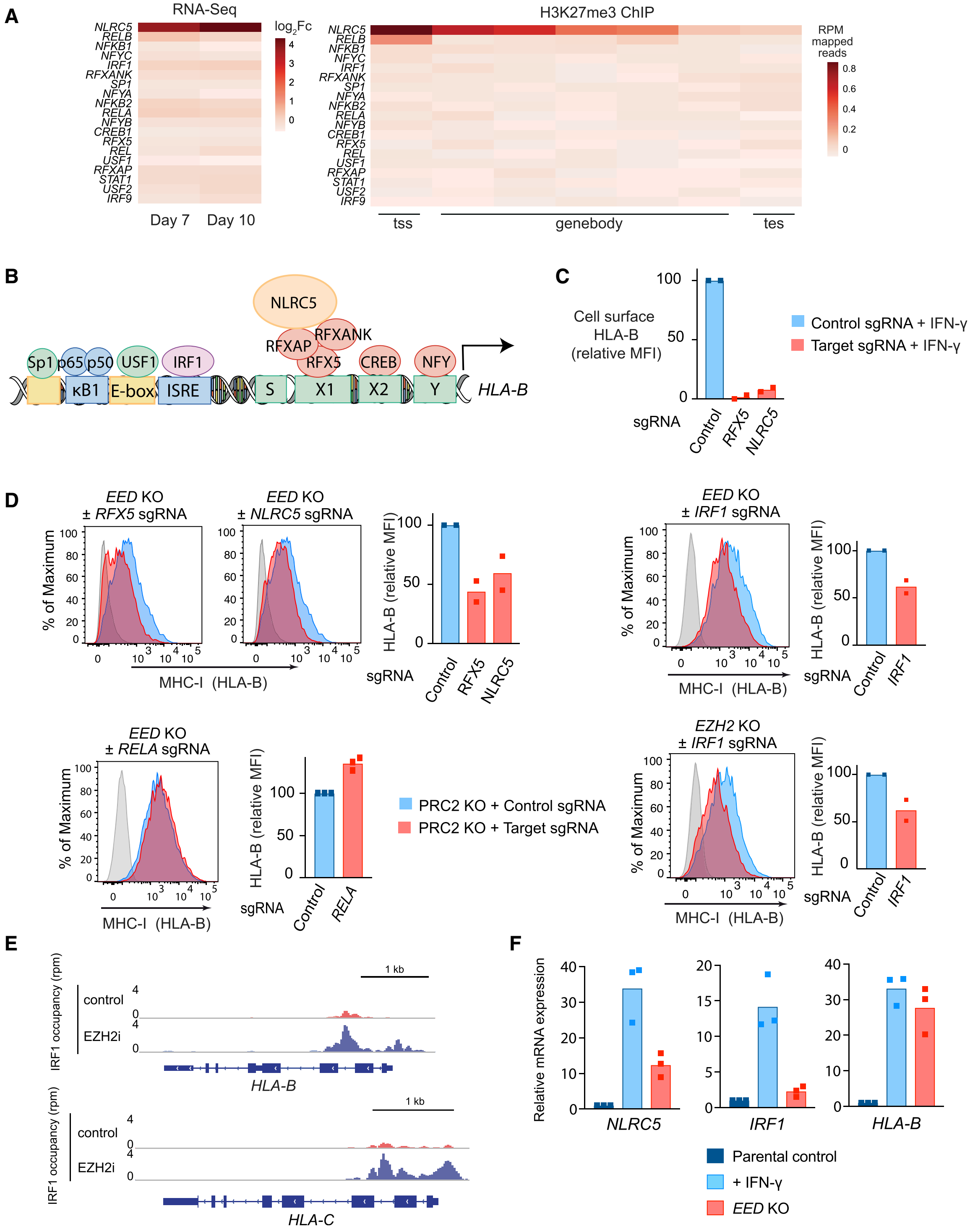

Figure 8. Disruption of PRC2 Leads to Derepression of *NLRC5* and Enhanced IRF1 Binding at MHC-I Gene *cis*-Regulatory Regions

(A) RNA-seq and H3K27me3 ChIP-seq data in K-562 cells displaying reported transcriptional regulators of MHC-I expression. RNA-seq heatmap shows log$_2$FC gene expression of K-562 cells expressing an *EED* sgRNA compared with control sgRNA at 7 and 10 days post-transduction. The H3K27me3 ChIP-seq heatmap shows the number of reads at 2 kb upstream of tss, genebody and 2 kb downstream of tes (transcription end site) of each gene.

(B) A schematic presentation of *cis*-regulatory elements in the *HLA-B* promoter. NLRC5 forms an enhanceosome with the regulatory factor X (RFX) complex, comprising RFX5, RFXANK (RFX-associated ankyrin-containing protein), and RFXAP (RFX-associated protein). CREB (cAMP-responsive-element-binding protein 1); NFY (nuclear transcription factor Y); ISRE (IFN-stimulated response element); USF1 (upstream stimulatory factor 1).

(C) Cell surface MHC-I in K-562 Cas9 cells expressing control, *NLRC5*-, or *RFX5*-specific sgRNA following incubation with IFN-γ 10 ng/mL for 24 h.

(legend continued on next page)

also through non-genomic mechanisms that exploit the activity of repressive chromatin complexes such as PRC2.

STAR★METHODS

Detailed methods are provided in the online version of this paper and include the following:

- KEY RESOURCES TABLE
- LEAD CONTACT AND MATERIALS AVAILABILITY
- EXPERIMENTAL MODEL AND SUBJECT DETAILS
  - Human Lung Cancer Samples
  - Clinical Details
  - Cell Lines
  - Animal Experiments
- METHOD DETAILS
  - Chemicals
  - Plasmids
  - CRISPR sgRNA Library
  - CRISPR Screen
  - CRISPR/Cas9-Mediated Gene Disruption and Generation of Knockout Clones
  - Lentiviral Production and Transduction
  - Flow Cytometry
  - Immunoblotting
  - Fluorescent Multiplex Immunohistochemistry
  - Isolation and In Vitro Activation of $CD8^+$ T Cells
  - T-cell Cytotoxicity Assays
  - Measurement of T-cell Cytokine Secretion
  - RNA-Sequencing
  - RNA-Sequencing Analysis
  - Chromatin Immunoprecipitation (ChIP)
  - ChIP-Sequencing Analysis
- QUANTIFICATION AND STATISTICAL ANALYSIS
- DATA AND CODE AVAILABILITY

SUPPLEMENTAL INFORMATION

Supplemental Information can be found online at https://doi.org/10.1016/j.ccell.2019.08.008.

ACKNOWLEDGMENTS

We thank the Peter MacCallum Cancer Center Molecular Genomics Core and the flow cytometry facilities at the Peter MacCallum Cancer Center and Cambridge Institute for Medical Research. We thank Andrew Flies and Jocelyn Darby (University of Tasmania) for the DFT cells. We thank the following funders for fellowship, scholarship, and grant support: Cancer Research UK Clinician Scientist Fellowship C53779/A20097 (to M.L.B.), Leukaemia Foundation Australia Senior Fellowship and Howard Hughes Medical Institute International Research Scholarship 55008729 (to M.A.D.), Peter and Julie Alston Centenary fellowship (to K.D.S.), Wellcome Trust Principal Research Fellowship 101835/Z/13/Z (to P.J.L.), Peter MacCallum Postgraduate Scholarship (to C.E.S.), NHMRC Postgraduate Scholarship (to K.L.C.), Maddie Riewoldt's Vision 064728 (to Y.-C.C.), Victorian Cancer Agency (to E.Y.N.L.), CSL Centenary fellowship (to S.-J.D.), National Breast Cancer Foundation Fellowship ECF-17-005 (to P.A.B.), Addenbrooke's Charitable Trust and NIHR Cambridge BRC (to M.L.B. and P.J.L.), NHMRC grants 1085015, 1106444 (to M.A.D) and 1128984 (to M.A.D. and S.-J.D.).

AUTHOR CONTRIBUTIONS

M.L.B., P.J.L., K.D.S., and M.A.D. designed the research and interpreted data. M.L.B. and M.A.D. supervised the research and wrote the manuscript. M.L.B., C.E.S., K.L.C., A.K., C.C.B., O.G., S.J., S.Q.W., L.T., S.H., G.R.G., and E.A.-Y. conducted experiments and analyzed data. M.L.B. conducted the CRISPR screen and E.Y.N.L. analyzed the screen data. Y.-C.C., D.V., and E.Y.N.L. analyzed the genomic data. A.K. and S.J. performed animal experiments supervised by K.D.S. and C.C. E.M.M., H.V.S., M.T.McC., R.K.P., B.J.S., S.S., S.-J.D., P.A.B., R.T., and C.C. provided critical expertise and/or reagents and contributed to manuscript preparation.

DECLARATION OF INTERESTS

M.T.M. and R.K.P. are employees of GlaxoSmithKline. M.A.D. has been a member of advisory boards for CTX CRC, Storm Therapeutics, Celgene, and Cambridge Epigenetix.

Received: March 27, 2019
Revised: June 26, 2019
Accepted: August 24, 2019
Published: September 26, 2019

REFERENCES

Abou El, H.M., Huang, K., Eswara, M.B., Zhao, M., Song, L., Yu, T., Liu, Y., Liu, J.C., McCurdy, S., Ma, A., et al. (2015). Cancer cells hijack PRC2 to modify multiple cytokine pathways. PLoS One *10*, e0126466.

Aggarwal, R., Huang, J., Alumkal, J.J., Zhang, L., Feng, F.Y., Thomas, G.V., Weinstein, A.S., Friedl, V., Zhang, C., Witte, O.N., et al. (2018). Clinical and genomic characterization of treatment-emergent small-cell neuroendocrine prostate cancer: a multi-institutional prospective study. J. Clin. Oncol. *36*, 2492–2503.

Agudo, J., Park, E.S., Rose, S.A., Alibo, E., Sweeney, R., Dhainaut, M., Kobayashi, K.S., Sachidanandam, R., Baccarini, A., Merad, M., and Brown, B.D. (2018). Quiescent tissue stem cells evade immune surveillance. Immunity *48*, 271–285.

Alexandrov, L.B., Nik-Zainal, S., Wedge, D.C., Aparicio, S.A., Behjati, S., Biankin, A.V., Bignell, G.R., Bolli, N., Borg, A., Borresen-Dale, A.L., et al. (2013). Signatures of mutational processes in human cancer. Nature *500*, 415–421.

Ardehali, M.B., Anselmo, A., Cochrane, J.C., Kundu, S., Sadreyev, R.I., and Kingston, R.E. (2017). Polycomb repressive complex 2 methylates elongin A to regulate transcription. Mol. Cell *68*, 872–884.e6.

Aubrey, B.J., Kelly, G.L., Kueh, A.J., Brennan, M.S., O'Connor, L., Milla, L., Wilcox, S., Tai, L., Strasser, A., and Herold, M.J. (2015). An inducible lentiviral guide RNA platform enables the identification of tumor-essential genes and tumor-promoting mutations in vivo. Cell Rep. *10*, 1422–1432.

Barretina, J., Caponigro, G., Stransky, N., Venkatesan, K., Margolin, A.A., Kim, S., Wilson, C.J., Lehar, J., Kryukov, G.V., Sonkin, D., et al. (2012). The Cancer Cell Line Encyclopedia enables predictive modelling of anticancer drug sensitivity. Nature *483*, 603–607.

(D) Cell surface MHC-I in *EED* or *EZH2* KO K-562 Cas9 cells transduced with either control sgRNA or *NLRC5*-, *RFX5*-, *RELA*-, or *IRF1*-specific sgRNA. Bars represent mean fold change in expression from independent experiments indicated by points.
(E) IRF1 ChIP-seq in K-562 cells treated with EZH2i (EPZ-011989 3 μM) or ethanol for 10 days. Plots show IRF1 occupancy at MHC-I genes.
(F) qRT-PCR analysis of MHC-I gene expression in *EED* KO K-562 compared with IFN-γ-treated control cells. Bars indicate mean fold change in expression relative to untreated control parental K-562 from independent experiments.
See also Figure S8.

Bernards, R., Dessain, S.K., and Weinberg, R.A. (1986). N-myc amplification causes down-modulation of MHC class I antigen expression in neuroblastoma. Cell *47*, 667–674.

Bernstein, B.E., Mikkelsen, T.S., Xie, X., Kamal, M., Huebert, D.J., Cuff, J., Fry, B., Meissner, A., Wernig, M., Plath, K., et al. (2006). A bivalent chromatin structure marks key developmental genes in embryonic stem cells. Cell *125*, 315–326.

Bracken, A.P., Pasini, D., Capra, M., Prosperini, E., Colli, E., and Helin, K. (2003). EZH2 is downstream of the pRB-E2F pathway, essential for proliferation and amplified in cancer. EMBO J. *22*, 5323–5335.

Burr, M.L., Sparbier, C.E., Chan, Y.C., Williamson, J.C., Woods, K., Beavis, P.A., Lam, E.Y.N., Henderson, M.A., Bell, C.C., Stolzenburg, S., et al. (2017). CMTM6 maintains the expression of PD-L1 and regulates anti-tumour immunity. Nature *549*, 101–105.

Canadas, I., Thummalapalli, R., Kim, J.W., Kitajima, S., Jenkins, R.W., Christensen, C.L., Campisi, M., Kuang, Y., Zhang, Y., Gjini, E., et al. (2018). Tumor innate immunity primed by specific interferon-stimulated endogenous retroviruses. Nat. Med. *24*, 1143–1150.

Chen, L., Alexe, G., Dharia, N.V., Ross, L., Iniguez, A.B., Conway, A.S., Wang, E.J., Veschi, V., Lam, N., Qi, J., et al. (2018). CRISPR-Cas9 screen reveals a MYCN-amplified neuroblastoma dependency on EZH2. J. Clin. Invest. *128*, 446–462.

Chiappinelli, K.B., Strissel, P.L., Desrichard, A., Li, H., Henke, C., Akman, B., Hein, A., Rote, N.S., Cope, L.M., Snyder, A., et al. (2015). Inhibiting DNA methylation causes an interferon response in cancer via dsRNA including endogenous retroviruses. Cell *162*, 974–986.

Comet, I., Riising, E.M., Leblanc, B., and Helin, K. (2016). Maintaining cell identity: PRC2-mediated regulation of transcription and cancer. Nat. Rev. Cancer *16*, 803–810.

Diebold, S.S., Cotten, M., Koch, N., and Zenke, M. (2001). MHC class II presentation of endogenously expressed antigens by transfected dendritic cells. Gene Ther. *8*, 487–493.

Durbin, J.E., Hackenmiller, R., Simon, M.C., and Levy, D.E. (1996). Targeted disruption of the mouse Stat1 gene results in compromised innate immunity to viral disease. Cell *84*, 443–450.

ENCODE Project Consortium (2012). An integrated encyclopedia of DNA elements in the human genome. Nature *489*, 57–74.

Flies, A.S., Lyons, A.B., Corcoran, L.M., Papenfuss, A.T., Murphy, J.M., Knowles, G.W., Woods, G.M., and Hayball, J.D. (2016). PD-L1 is not constitutively expressed on Tasmanian devil facial tumor cells but is strongly upregulated in response to IFN-gamma and can be expressed in the tumor microenvironment. Front. Immunol. *7*, 581.

Gao, J., Shi, L.Z., Zhao, H., Chen, J., Xiong, L., He, Q., Chen, T., Roszik, J., Bernatchez, C., Woodman, S.E., et al. (2016). Loss of IFN-gamma pathway genes in tumor cells as a mechanism of resistance to anti-CTLA-4 therapy. Cell *167*, 397–404.

Gardner, E.E., Lok, B.H., Schneeberger, V.E., Desmeules, P., Miles, L.A., Arnold, P.K., Ni, A., Khodos, I., de, S.E., Nguyen, T., et al. (2017). Chemosensitive relapse in small cell lung cancer proceeds through an EZH2-SLFN11 axis. Cancer Cell *31*, 286–299.

George, J., Lim, J.S., Jang, S.J., Cun, Y., Ozretic, L., Kong, G., Leenders, F., Lu, X., Fernandez-Cuesta, L., Bosco, G., et al. (2015). Comprehensive genomic profiles of small cell lung cancer. Nature *524*, 47–53.

Gilan, O., Lam, E.Y., Becher, I., Lugo, D., Cannizzaro, E., Joberty, G., Ward, A., Wiese, M., Fong, C.Y., Ftouni, S., et al. (2016). Functional interdependence of BRD4 and DOT1L in MLL leukemia. Nat. Struct. Mol. Biol. *23*, 673–681.

Goswami, S., Apostolou, I., Zhang, J., Skepner, J., Anandhan, S., Zhang, X., Xiong, L., Trojer, P., Aparicio, A., Subudhi, S.K., et al. (2018). Modulation of EZH2 expression in T cells improves efficacy of anti-CTLA-4 therapy. J. Clin. Invest. *128*, 3813–3818.

Gubin, M.M., Zhang, X., Schuster, H., Caron, E., Ward, J.P., Noguchi, T., Ivanova, Y., Hundal, J., Arthur, C.D., Krebber, W.J., et al. (2014). Checkpoint blockade cancer immunotherapy targets tumour-specific mutant antigens. Nature *515*, 577–581.

Harutyunyan, A.S., Krug, B., Chen, H., Papillon-Cavanagh, S., Zeinieh, M., De Jay, N., Deshmukh, S., Chen, C.C.L., Belle, J., Mikael, L.G., et al. (2019). H3K27M induces defective chromatin spread of PRC2-mediated repressive H3K27me2/me3 and is essential for glioma tumorigenesis. Nat. Commun. *10*, 1262.

Holoch, D., and Margueron, R. (2017). Mechanisms regulating PRC2 recruitment and enzymatic activity. Trends Biochem. Sci. *42*, 531–542.

Jiang, L., Huang, J., Higgs, B.W., Hu, Z., Xiao, Z., Yao, X., Conley, S., Zhong, H., Liu, Z., Brohawn, P., et al. (2016). Genomic landscape survey identifies SRSF1 as a key oncodriver in small cell lung cancer. PLoS Genet. *12*, e1005895.

Karantanos, T., Chistofides, A., Barhdan, K., Li, L., and Boussiotis, V.A. (2016). Regulation of T cell differentiation and function by EZH2. Front. Immunol. *7*, 172.

Kearney, C.J., Vervoort, S.J., Hogg, S.J., Ramsbottom, K.M., Freeman, A.J., Lalaoui, N., Pijpers, L., Michie, J., Brown, K.K., Knight, D.A., et al. (2018). Tumor immune evasion arises through loss of TNF sensitivity. Sci. Immunol. *3*, https://doi.org/10.1126/sciimmunol.aar3451.

Kobayashi, K.S., and van den Elsen, P.J. (2012). NLRC5: a key regulator of MHC class I-dependent immune responses. Nat. Rev. Immunol. *12*, 813–820.

Koike-Yusa, H., Li, Y., Tan, E.P., Velasco-Herrera Mdel, C., and Yusa, K. (2014). Genome-wide recessive genetic screening in mammalian cells with a lentiviral CRISPR-guide RNA library. Nat. Biotechnol. *32*, 267–273.

Konig, R., Chiang, C.Y., Tu, B.P., Yan, S.F., DeJesus, P.D., Romero, A., Bergauer, T., Orth, A., Krueger, U., Zhou, Y., and Chanda, S.K. (2007). A probability-based approach for the analysis of large-scale RNAi screens. Nat. Methods *4*, 847–849.

Kulkarni, S., Qi, Y., O'HUigin, C., Pereyra, F., Ramsuran, V., McLaren, P., Fellay, J., Nelson, G., Chen, H., Liao, W., et al. (2013). Genetic interplay between HLA-C and MIR148A in HIV control and Crohn disease. Proc. Natl. Acad. Sci. U S A *110*, 20705–20710.

Kundaje, A., Meuleman, W., Ernst, J., Bilenky, M., Yen, A., Heravi-Moussavi, A., Kheradpour, P., Zhang, Z., Wang, J., Ziller, M.J., et al. (2015). Integrative analysis of 111 reference human epigenomes. Nature *518*, 317–330.

Landsberg, J., Kohlmeyer, J., Renn, M., Bald, T., Rogava, M., Cron, M., Fatho, M., Lennerz, V., Wolfel, T., Holzel, M., and Tuting, T. (2012). Melanomas resist T-cell therapy through inflammation-induced reversible dedifferentiation. Nature *490*, 412–416.

Langmead, B., and Salzberg, S.L. (2012). Fast gapped-read alignment with Bowtie 2. Nat. Methods *9*, 357–359.

Lee, C.H., Holder, M., Grau, D., Saldana-Meyer, R., Yu, J.R., Ganai, R.A., Zhang, J., Wang, M., Leroy, G., Dobenecker, M.W., et al. (2018). Distinct stimulatory mechanisms regulate the catalytic activity of polycomb repressive complex 2. Mol. Cell *70*, 435–448.

Lewis, P.W., Muller, M.M., Koletsky, M.S., Cordero, F., Lin, S., Banaszynski, L.A., Garcia, B.A., Muir, T.W., Becher, O.J., and Allis, C.D. (2013). Inhibition of PRC2 activity by a gain-of-function H3 mutation found in pediatric glioblastoma. Science *340*, 857–861.

Li, H., Liefke, R., Jiang, J., Kurland, J.V., Tian, W., Deng, P., Zhang, W., He, Q., Patel, D.J., Bulyk, M.L., et al. (2017). Polycomb-like proteins link the PRC2 complex to CpG islands. Nature *549*, 287–291.

Manguso, R.T., Pope, H.W., Zimmer, M.D., Brown, F.D., Yates, K.B., Miller, B.C., Collins, N.B., Bi, K., LaFleur, M.W., Juneja, V.R., et al. (2017). In vivo CRISPR screening identifies Ptpn2 as a cancer immunotherapy target. Nature *547*, 413–418.

Margueron, R., Justin, N., Ohno, K., Sharpe, M.L., Son, J., Drury, W.J., III, Voigt, P., Martin, S.R., Taylor, W.R., De Marco, V., et al. (2009). Role of the polycomb protein EED in the propagation of repressive histone marks. Nature *461*, 762–767.

Marincola, F.M., Shamamian, P., Simonis, T.B., Abati, A., Hackett, J., O'Dea, T., Fetsch, P., Yannelli, J., Restifo, N.P., and Mule, J.J. (1994). Locus-specific analysis of human leukocyte antigen class I expression in melanoma cell lines. J. Immunother. Emphasis Tumor Immunol. *16*, 13–23.

Mehta, A., Kim, Y.J., Robert, L., Tsoi, J., Comin-Anduix, B., Berent-Maoz, B., Cochran, A.J., Economou, J.S., Tumeh, P.C., Puig-Saus, C., and Ribas, A. (2018). Immunotherapy resistance by inflammation-induced dedifferentiation. Cancer Discov. *8*, 935–943.

Meuwissen, R., Linn, S.C., Linnoila, R.I., Zevenhoven, J., Mooi, W.J., and Berns, A. (2003). Induction of small cell lung cancer by somatic inactivation of both Trp53 and Rb1 in a conditional mouse model. Cancer Cell *4*, 181–189.

Minoux, M., Holwerda, S., Vitobello, A., Kitazawa, T., Kohler, H., Stadler, M.B., and Rijli, F.M. (2017). Gene bivalency at Polycomb domains regulates cranial neural crest positional identity. Science *355*, eaal2913.

Morgens, D.W., Wainberg, M., Boyle, E.A., Ursu, O., Araya, C.L., Tsui, C.K., Haney, M.S., Hess, G.T., Han, K., Jeng, E.E., et al. (2017). Genome-scale measurement of off-target activity using Cas9 toxicity in high-throughput screens. Nat. Commun. *8*, 15178.

Murchison, E.P., Tovar, C., Hsu, A., Bender, H.S., Kheradpour, P., Rebbeck, C.A., Obendorf, D., Conlan, C., Bahlo, M., Blizzard, C.A., et al. (2010). The Tasmanian devil transcriptome reveals Schwann cell origins of a clonally transmissible cancer. Science *327*, 84–87.

Oser, M.G., Niederst, M.J., Sequist, L.V., and Engelman, J.A. (2015). Transformation from non-small-cell lung cancer to small-cell lung cancer: molecular drivers and cells of origin. Lancet Oncol. *16*, e165–e172.

Pan, D., Kobayashi, A., Jiang, P., Ferrari de, A.L., Tay, R.E., Luoma, A.M., Tsoucas, D., Qiu, X., Lim, K., Rao, P., et al. (2018). A major chromatin regulator determines resistance of tumor cells to T cell-mediated killing. Science *359*, 770–775.

Patel, S.J., Sanjana, N.E., Kishton, R.J., Eidizadeh, A., Vodnala, S.K., Cam, M., Gartner, J.J., Jia, L., Steinberg, S.M., Yamamoto, T.N., et al. (2017). Identification of essential genes for cancer immunotherapy. Nature *548*, 537–542.

Paulson, K.G., Voillet, V., McAfee, M.S., Hunter, D.S., Wagener, F.D., Perdicchio, M., Valente, W.J., Koelle, S.J., Church, C.D., Vandeven, N., et al. (2018). Acquired cancer resistance to combination immunotherapy from transcriptional loss of class I HLA. Nat. Commun. *9*, 3868.

Peng, D., Kryczek, I., Nagarsheth, N., Zhao, L., Wei, S., Wang, W., Sun, Y., Zhao, E., Vatan, L., Szeliga, W., et al. (2015). Epigenetic silencing of TH1-type chemokines shapes tumour immunity and immunotherapy. Nature *527*, 249–253.

Qi, W., Zhao, K., Gu, J., Huang, Y., Wang, Y., Zhang, H., Zhang, M., Zhang, J., Yu, Z., Li, L., et al. (2017). An allosteric PRC2 inhibitor targeting the H3K27me3 binding pocket of EED. Nat. Chem. Biol. *13*, 381–388.

Ramsuran, V., Hernandez-Sanchez, P.G., O'HUigin, C., Sharma, G., Spence, N., Augusto, D.G., Gao, X., Garcia-Sepulveda, C.A., Kaur, G., Mehra, N.K., and Carrington, M. (2017). Sequence and phylogenetic analysis of the untranslated promoter regions for HLA class I genes. J. Immunol. *198*, 2320–2329.

Rene, C., Lozano, C., and Eliaou, J.F. (2016). Expression of classical HLA class I molecules: regulation and clinical impacts: Julia Bodmer Award Review 2015. HLA *87*, 338–349.

Restifo, N.P., Esquivel, F., Kawakami, Y., Yewdell, J.W., Mule, J.J., Rosenberg, S.A., and Bennink, J.R. (1993). Identification of human cancers deficient in antigen processing. J. Exp. Med. *177*, 265–272.

Roulois, D., Loo, Y.H., Singhania, R., Wang, Y., Danesh, A., Shen, S.Y., Han, H., Liang, G., Jones, P.A., Pugh, T.J., et al. (2015). DNA-demethylating agents target colorectal cancer cells by inducing viral mimicry by endogenous transcripts. Cell *162*, 961–973.

Sabari, J.K., Lok, B.H., Laird, J.H., Poirier, J.T., and Rudin, C.M. (2017). Unravelling the biology of SCLC: implications for therapy. Nat. Rev. Clin. Oncol. *14*, 549–561.

Sato, T., Kaneda, A., Tsuji, S., Isagawa, T., Yamamoto, S., Fujita, T., Yamanaka, R., Tanaka, Y., Nukiwa, T., Marquez, V.E., et al. (2013). PRC2 overexpression and PRC2-target gene repression relating to poorer prognosis in small cell lung cancer. Sci. Rep. *3*, 1911.

Sheng, W., LaFleur, M.W., Nguyen, T.H., Chen, S., Chakravarthy, A., Conway, J.R., Li, Y., Chen, H., Yang, H., Hsu, P.H., et al. (2018). LSD1 ablation stimulates anti-tumor immunity and enables checkpoint blockade. Cell *174*, 549–563.

Shin, D.S., Zaretsky, J.M., Escuin-Ordinas, H., Garcia-Diaz, A., Hu-Lieskovan, S., Kalbasi, A., Grasso, C.S., Hugo, W., Sandoval, S., Torrejon, D.Y., et al. (2017). Primary resistance to PD-1 blockade mediated by JAK1/2 mutations. Cancer Discov. *7*, 188–201.

Siddle, H.V. (2017). Cancer as a contagious disease. HLA *89*, 209–214.

Siddle, H.V., Kreiss, A., Tovar, C., Yuen, C.K., Cheng, Y., Belov, K., Swift, K., Pearse, A.M., Hamede, R., Jones, M.E., et al. (2013). Reversible epigenetic down-regulation of MHC molecules by devil facial tumour disease illustrates immune escape by a contagious cancer. Proc. Natl. Acad. Sci. U S A *110*, 5103–5108.

Stafford, J.M., Lee, C.H., Voigt, P., Descostes, N., Saldana-Meyer, R., Yu, J.R., Leroy, G., Oksuz, O., Chapman, J.R., Suarez, F., et al. (2018). Multiple modes of PRC2 inhibition elicit global chromatin alterations in H3K27M pediatric glioma. Sci. Adv. *4*, eaau5935.

Sutherland, K.D., Proost, N., Brouns, I., Adriaensen, D., Song, J.Y., and Berns, A. (2011). Cell of origin of small cell lung cancer: inactivation of Trp53 and Rb1 in distinct cell types of adult mouse lung. Cancer Cell *19*, 754–764.

Tilburgs, T., and Strominger, J.L. (2013). CD8+ effector T cells at the fetal-maternal interface, balancing fetal tolerance and antiviral immunity. Am. J. Reprod. Immunol. *69*, 395–407.

Tumeh, P.C., Harview, C.L., Yearley, J.H., Shintaku, I.P., Taylor, E.J., Robert, L., Chmielowski, B., Spasic, M., Henry, G., Ciobanu, V., et al. (2014). PD-1 blockade induces responses by inhibiting adaptive immune resistance. Nature *515*, 568–571.

Ueda, T., Sanada, M., Matsui, H., Yamasaki, N., Honda, Z.I., Shih, L.Y., Mori, H., Inaba, T., Ogawa, S., and Honda, H. (2012). EED mutants impair polycomb repressive complex 2 in myelodysplastic syndrome and related neoplasms. Leukemia *26*, 2557–2560.

van 't Veer, L.J., Beijersbergen, R.L., and Bernards, R. (1993). N-myc suppresses major histocompatibility complex class I gene expression through down-regulation of the p50 subunit of NF-kappa B. EMBO J. *12*, 195–200.

Voigt, P., Tee, W.W., and Reinberg, D. (2013). A double take on bivalent promoters. Genes Dev. *27*, 1318–1338.

Wang, L., Jin, Q., Lee, J.E., Su, I.H., and Ge, K. (2010). Histone H3K27 methyltransferase Ezh2 represses Wnt genes to facilitate adipogenesis. Proc. Natl. Acad. Sci. U S A *107*, 7317–7322.

Wang, D., Quiros, J., Mahuron, K., Pai, C.C., Ranzani, V., Young, A., Silveria, S., Harwin, T., Abnousian, A., Pagani, M., et al. (2018). Targeting EZH2 reprograms intratumoral regulatory T cells to enhance cancer immunity. Cell Rep. *23*, 3262–3274.

Wassef, M., and Margueron, R. (2017). The multiple facets of PRC2 alterations in cancers. J. Mol. Biol. *429*, 1978–1993.

Wee, Z.N., Li, Z., Lee, P.L., Lee, S.T., Lim, Y.P., and Yu, Q. (2014). EZH2-mediated inactivation of IFN-gamma-JAK-STAT1 signaling is an effective therapeutic target in MYC-driven prostate cancer. Cell Rep. *8*, 204–216.

Zaretsky, J.M., Garcia-Diaz, A., Shin, D.S., Escuin-Ordinas, H., Hugo, W., Hu-Lieskovan, S., Torrejon, D.Y., Abril-Rodriguez, G., Sandoval, S., Barthly, L., et al. (2016). Mutations associated with acquired resistance to PD-1 blockade in melanoma. N. Engl. J. Med. *375*, 819–829.

Zeid, R., Lawlor, M.A., Poon, E., Reyes, J.M., Fulciniti, M., Lopez, M.A., Scott, T.G., Nabet, B., Erb, M.A., Winter, G.E., et al. (2018). Enhancer invasion shapes MYCN-dependent transcriptional amplification in neuroblastoma. Nat. Genet. *50*, 515–523.

Zingg, D., Arenas-Ramirez, N., Sahin, D., Rosalia, R.A., Antunes, A.T., Haeusel, J., Sommer, L., and Boyman, O. (2017). The histone methyltransferase Ezh2 controls mechanisms of adaptive resistance to tumor immunotherapy. Cell Rep. *20*, 854–867.

STAR★METHODS

KEY RESOURCES TABLE

REAGENT or RESOURCE	SOURCE	IDENTIFIER
Antibodies		
Alexa Fluor 488 monoclonal mouse anti-human HLA-A,B,C	BioLegend	Cat# 311413; clone W6/32; RRID: AB_493133
APC monoclonal mouse anti-human HLA-A,B,C	BioLegend	Cat# 311410; clone W6/32; RRID: AB_314879
APC-Vio770 anti-HLA class I Bw6	Miltenyi Biotec	Cat# 130-099-837; clone REA143; RRID: AB_2652034
APC anti-HLA class I Bw6	Miltenyi Biotec	Cat# 130-099-845; clone REA143; RRID: AB_2652026
APC mouse anti-mouse MHC class I (H-2Kb)	eBioscience, Thermo Fisher Scientific	Cat# 17-5958-82; clone AF6-88.5.5.3; RRID: AB_1311280
APC anti-mouse H-2Kb/SIINFEKL	Miltenyi Biotec	Cat#130-103-024; Clone 25-D1.16; RRID: AB_2651952
Monoclonal mouse anti-human MHC class I heavy chain	Origene	Cat# AM33035PU-N; Clone HC10; RRID: AB_2728622
Monoclonal mouse anti-HSP60 (C-10)	Santa Cruz	Cat# sc-376240; RRID: AB_10986282
Monoclonal mouse anti-human EZH2	BD Transduction Laboratories	Cat# 563491; clone 11/EZH2; RRID: AB_2738239
Monoclonal Rabbit anti-H3K27me3 (Lys27)	Cell Signaling Technology	Cat# 9733; clone C36B11; RRID: AB_2616029
Polyclonal rabbit anti-H3K27ac	Abcam	Cat# ab4729; RRID: AB_2118291
Polyclonal rabbit anti-H3K4me3	Abcam	Cat# ab8580; RRID: AB_306649
Monoclonal rabbit anti-IRF1	Cell Signaling Technology	Cat# 8478; clone D5E4; RRID: AB_10949108
Polyclonal rabbit anti-STAT1	Merck-Millipore	Cat# 06-501; RRID: AB_310145
Monoclonal rabbit anti-phospho-Stat1 (Tyr701)	Cell Signaling Technology	Cat# 9167 Clone 58D6; RRID: AB_561284
Polyclonal rabbit anti-mouse NCAM	Abcam	Cat# ab95153; RRID: AB_10975468
Monoclonal rabbit anti-CD3	Abcam	Cat# ab16669; clone SP7; RRID: AB_443425
Monoclonal rabbit anti-mouse CD8 alpha	Abcam	Cat# ab209775; clone EPR20305
FITC Monoclonal rat anti-mouse CD45	BioLegend	Cat# 103108; clone 30-F11; RRID: AB_312973
APC monoclonal rat anti-mouse CD3	BioLegend	Cat# 100236; clone 17A2; RRID: AB_2561456
PE monoclonal rat anti-mouse CD8a	BioLegend	Cat# 100708; clone 53-6.7; RRID: AB_312747
Pacific Blue anti-mouse CD31	BioLegend	Cat# 102422; clone 390; RRID: AB_10612926
Pacific Blue anti-mouse TER-119	BioLegend	Cat# 116232, clone TER-119; RRID: AB_2251160
Chemicals, Peptides, and Recombinant Proteins		
EPZ-011989 (EZH2 inhibitor)	Selleck Chemicals	Cat# S7805; CASRN: 1598383-40-4
EED-226 (EED inhibitor)	Selleck Chemicals	Cat# S8496; CASRN: 2083627-02-3
GSK-503 (EZH2 inhibitor)	Selleck Chemicals	Cat# S7804; CASRN: 1346572-63-1
Recombinant human interferon gamma	Sigma-Aldrich	Cat# I17001
Recombinant mouse interferon gamma	Abcam	Cat# ab9922
Recombinant mouse interleukin-2	Abcam	Cat# ab9856
SIINFEKL (OVA peptide)	Sigma-Aldrich	Cat# S7951; CASRN: 138831-86-4
Recombinant Tasmanian Devil interferon gamma	Andrew Flies, University of Tasmania	Flies et al., 2016
Critical Commercial Assays		
Cytometric bead array Mouse Th1/Th2 cytokine kit	BD Biosciences	Cat# 551287

(Continued on next page)

Continued

REAGENT or RESOURCE	SOURCE	IDENTIFIER
Deposited Data		
Sequencing Data (ChIP-seq, RNAseq, CRISPR screen)	This paper	NCBI: PRJNA527170; GEO: GSE129382
Mendeley Dataset unprocessed scans	This paper	https://doi.org/10.17632/yf4vcxy469.1
ChIP-seq: mouse neural crest	(Minoux et al., 2017)	GEO: GSE89435
ChIP-seq: BE2-C neuroblastoma	(Zeid et al., 2018)	GEO: GSE80151
ChIP-seq: NT2-D1 embryonal carcinoma	(ENCODE Project Consortium, 2012)	GEO: GSE31755, https://www.encodeproject.org/
H3K27me3, H3K4me3, H3K27ac and H3K36me3 ChIPseq data from NIH Roadmap Epigenomics Mapping Consortium	(Kundaje et al., 2015)	http://www.roadmapepigenomics.org/
Cancer Cell Line Encyclopaedia	(Barretina et al., 2012)	https://portals.broadinstitute.org/ccle
RNAseq primary SCLC	(Jiang et al., 2016)	GSE60052
RNAseq primary SCLC	(Sato et al., 2013)	GSE43346
Experimental Models: Cell Lines		
Human: Kelly	Paul Ekert (Murdoch Children's Research Institute)	RRID: CVCL_2092
Human: IMR-32	Paul Ekert (Murdoch Children's Research Institute)	RRID: CVCL_0346
Human: NCI-H82	Jonathan Yewdell (National Institute of Allergy and Infectious Diseases)	RRID: CVCL_1591
Human: NCI-H146	Jonathan Yewdell (National Institute of Allergy and Infectious Diseases)	RRID: CVCL_1473
Human: NCI-H69	ATCC	RRID: CVCL_1579
Tasmanian Devil Facial Tumor 1 (DFT1) C5065	Andrew Flies (University of Tasmania)	RRID: CVCL_LB79
Human: K-562	ATCC	Cat#CCL-243; RRID: CVCL_0004
Human: HEK 293ET	Felix Randow (MRC-LMB, Cambridge, UK)	RRID: CVCL_6996
Human: MCC-002	This paper	
Drosophila: S2	ATCC	RRID: CRL-1963
Mouse: RP-48	This paper	
Mouse: RP-116	This paper	
Mouse: RP-186	This paper	
Experimental Models: Organisms/Strains		
Mouse: C57BL/6	Walter and Eliza Hall Institute	
Mouse: Balb/c	Walter and Eliza Hall Institute	
Mouse: C57BL/6 OTI	Walter and Eliza Hall Institute	
Mouse: NSG (NOD-scid IL2Rgamma^{null})	Walter and Eliza Hall Institute	
Oligonucleotides		
See Table S3	(Siddle et al., 2013; Kulkarni et al., 2013; Ramsuran et al., 2017)	
Recombinant DNA		
pCMV HA EED WT	(Bracken et al., 2003)	Addgene: 24231
MSCV-hygro-FLAG-Ezh2 wild-type	(Wang et al., 2010)	Addgene: 24926
MSCV-hygro-FLAG-Ezh2 F6671	(Wang et al., 2010)	Addgene: 24927
pCDH-EF1 HA-FLAG-H3.3 WT Puro	(Lewis et al., 2013)	
pCDH-EF1-HA-FLAG-H3.3 K27M Puro	(Lewis et al., 2013)	
pcDNA3-OVA	(Diebold et al., 2001)	Addgene: 64599
pHRSIN-P$_{SFFV}$-EZH2-P$_{PGK}$-Puro	This paper	

(*Continued on next page*)

REAGENT or RESOURCE	SOURCE	IDENTIFIER
Continued		
pHRSIN-P_{SFFV}-EZH2 F667I-P_{PGK}-Puro	This paper	
pHRSIN-P_{SFFV}-EED-P_{PGK}-Puro	This paper	
pHRSIN-P_{SFFV}-EED W364A -P_{PGK}-Puro	This paper	
MSCV-OVA-IRES-mCherry	This paper	
pKLV-U6gRNA(BbsI)-$Puro_{2A}$BFP	(Koike-Yusa et al., 2014)	Addgene: 50946
pHRSIN-P_{SFFV}-Cas9-P_{PGK}-Blasticidin	(Burr et al., 2017)	
FUCas9Cherry	(Aubrey et al., 2015)	Addgene: 70182
Bassik Human CRISPR Knockout Library	(Morgens et al., 2017)	Addgene: 101296-101934
Software and Algorithms		
Redundant siRNA activity (RSA)Algorithm	(Konig et al., 2007)	https://admin-ext.gnf.org/publications/RSA/
Fastx Clipper	Hannon Laboratory	http://hannonlab.cshl.edu/fastx_toolkit/
Bowtie2	(Langmead and Salzberg, 2012)	http://bowtie-bio.sourceforge.net/bowtie2/index.shtml
Bcl2fastq	Illumina	https://support.illumina.com/downloads/bcl2fastq-conversion-software-v2-20.html
HALO v2.2.1870	Indica Labs	http://www.indicalab.com/halo/
inForm	Perkin Elmer	Cat#CLS135781 https://www.perkinelmer.co.uk/product/inform-cell-analysis-one-seat-cls135781

LEAD CONTACT AND MATERIALS AVAILABILITY

Further information and requests for resources and reagents should be directed to and will be fulfilled by the Lead Contact, Mark Dawson (mark.dawson@petermac.org).

EXPERIMENTAL MODEL AND SUBJECT DETAILS

Human Lung Cancer Samples

Patient 1 and patient 3 participated in the CASCADE (CAncer tissue Collection After DEath) rapid autopsy program, which was approved by the Peter MacCallum Cancer Centre Ethics Committee (HREC 11/102). Patient 2 participated in the Thoracic Malignancies Prospective Cohort Study, approved by the Peter MacCallum Cancer Centre Ethics Committee (HREC 11/88). Informed consent was obtained from all patients.

Clinical Details

Patient 2 was a 63 year old female non-smoker, who was diagnosed with a primary lung adenocarcinoma with an EGFR exon 19 deletion. She underwent a right upper lobectomy to remove the tumor. 3 years later she was diagnosed with metastatic disease, and biopsy confirmed lung adenocarcinoma, which retained the original EGFR mutation (adenocarcinoma samples in Figure S6D). She was initially treated with the EGFR inhibitor Erlotinib, followed by Osimertinib after developing an EGFR T790M mutation that confers Erlotinib resistance. After 2 years of EGFR inhibitor treatment she developed progressive disease and biopsy showed small cell carcinoma, which remained positive for the EGFR exon 19 deletion (SCLC samples in Figure 6D). She received chemotherapy for the small cell carcinoma but had ongoing progressive disease and died 7 months later.

Patient 3 was a 59 year old female non-smoker, who was diagnosed with metastatic (stage 4) EGFR mutant lung adenocarcinoma. She was treated with the EGFR inhibitor Gefitinib. 16 months later she developed progressive disease and was treated with chemotherapy, followed by Erlotinib and then Afatinib as she failed to respond. She had ongoing progressive disease and repeat biopsy showed small cell transformation (SCLC samples in Figure 6D). She received chemotherapy followed by nivolumab, however failed to respond to this and died 3 months later.

Cell Lines

Human neuroblastoma cell lines Kelly and IMR-32 were a gift from Paul Ekert (Murdoch Children's Research Institute). Human SCLC lines NCI-H82 and NCI-H146 were a gift from Jonathan Yewdell (National Institute of Allergy and Infectious Diseases, Bethesda). Tasmanian Devil Facial Tumor 1 (DFT1) C5065 cells were a gift from Andrew Flies (University of Tasmania) and were established with approval from Tasmanian Government Department of Primary Industries, Parks, Water and the Environment (DPIPWE). HEK-293ET cells were a gift from Dr. Felix Randow (MRC-LMB, Cambridge, UK). K-562 and NCI-H69 cells were from ATCC. Human Merkel Cell Carcinoma line MCC-002 was derived from a patient with Merkel cell polyoma virus positive Merkel Cell Carcinoma. Informed

patient consent and Institutional Review Board approval were obtained according to the guidelines of the Australian National Health and Medical Research Council approved by the Peter MacCallum Cancer Centre Human Research Ethics Committee (Protocol: 14-113). The tumor was mechanically disaggregated to a single cell suspension and propagated in RPMI-1640, supplemented with 2 mM Glutamax, 100 IU/mL Penicillin, 100 μg/mL Streptomycin and 5% heat-inactivated fetal calf serum (HI-FCS). Mouse SCLC cell lines RP-48, RP-116 and RP-186 were established from primary lung tumors in a genetically engineered model driven by conditional bi-allelic inactivation of the tumor suppressor genes *Trp53* and *Rb1* (p53Rb) (Sutherland et al., 2011; Meuwissen et al., 2003). These mice carry conditional alleles for *Rb1* (floxed exon 19) and *Trp53* (floxed exons 2–10). Intra-nasal delivery of an adenoviral to CMV-Cre-recombinase vector to p53Rb mice results in somatic inactivation of *Trp53* and *Rb1* in pulmonary cells and the subsequent development of high-grade neuroendocrine tumors, which have been shown to be morphologically and phenotypically similar to human SCLC, with a median latency of 200-300 days (Sutherland et al., 2011; Meuwissen et al., 2003). Tumors were mechanically disaggregated and cultured in DMEM/F12 (Gibco) with 15 mM HEPES, supplemented with 2 mM Glutamax (Invitrogen), 100 IU/mL Penicillin, 100 μg/mL Streptomycin, 4 μg/mL Hydrocortisone (Sigma-Aldrich), 5 ng/mL EGF (Invitrogen), 5 mL of Insulin-Transferrin-Selenium solution (Life Technologies) and 10% HI-FCS. Tumor cells grew as spherical aggregates in suspension. Other human and DFT1 cell lines were cultured in RPMI-1640 or DMEM (HEK-293ET), supplemented with 2 mM Glutamax, 100 IU/mL Penicillin, 100 μg/mL Streptomycin and 10% HI-FCS. CD8 T cells isolated from mice were cultured in complete RPMI-1640 media (10% HI-FBS, 100 IU/mL Penicillin, 100 μg/mL Streptomycin, 1 mM sodium pyruvate, 2 mM Glutamax and 0.05 nM 2-mercaptoethanol. Drosophila S2 cells (ATCC catalog number CRL-1963; Biovest part number OO.763/OO.627) were cultured in Schneider's *Drosophila* media (Life Technologies) supplemented with 10% FBS. All cell lines were cultured in 5% CO_2 at 37°C with the exception of C5065 cells, which were cultured in 5% CO_2 at 35°C and Drosophila S2, which were cultured at room temperature.

Cell lines were authenticated by STR profiling through the Australian Genome Research Facility (Melbourne, Victoria). Cell lines were regularly tested and verified to be mycoplasma-negative by PCR analysis through the Victorian Infectious Diseases Reference Laboratory (Melbourne, Victoria).

Animal Experiments

For *in vivo* experiments, mSCLC RP-116 cells or RP-116 *Ezh2* KO clones, were resuspended in 50:50 PBS:Matrigel (Growth factor reduced) and injected subcutaneously in a 100 μL volume into female C57BL/6 or Balb/c mice, purchased from the Walter and Eliza Hall Institute (Melbourne, Australia). Experiments in Figures 6C and 6D were performed using *Ezh2* KO clone 1. The survival endpoint was when tumors reached a size of 500 mm^3. Mice were used between 6 and 12 weeks of age. All animal studies were approved in advance by the Peter MacCallum Animal Ethics and Experimentation Committee and the Walter and Eliza Hall Institute Animal Ethics Committee and all ethical obligations were met.

METHOD DETAILS

Chemicals

EPZ-011989, EED-226 and GSK-503 were purchased from Selleck Chemicals. For each inhibitor, titration experiments were performed to determine the maximally effective, tolerated dose in each cell line. Doses used and duration of treatment are indicated in the figure legends. Cells were typically treated for 7-12 days prior to analysis. Drugs were refreshed 3 times per week. For interferon induction of MHC-I expression, human cells were treated with recombinant human interferon gamma (Sigma-Aldrich) and mouse cells with recombinant mouse interferon gamma (Abcam) at a dose of 10ng/mL for 24 hours (unless specified otherwise in figure legend) prior to analysis. Recombinant Tasmanian devil interferon gamma was generated and purified as previously described (Flies et al., 2016).

Plasmids

pCDH-EF1-Puro lentiviral vectors encoding HA-FLAG-tagged wildtype H3.3 and H3.3 K27M were a kind gift from Peter Lewis (University of Wisconsin) (Lewis et al., 2013). To generate lentiviral EED and EZH2 expression plasmids, wild-type EED was PCR amplified from pCMV HA EED WT (Addgene 24231, a gift from Kristian Helin) (Bracken et al., 2003) and subcloned into pHRSIN-P_{SFFV}-eGFP-P_{PGK}-Puro via BamHI and XhoI, replacing the GFP. Mouse EZH2 and EZH2 F667I were subcloned into pHRSIN-P_{SFFV}-eGFP-P_{PGK}-Puro via BamHI and XhoI following PCR amplification from MSCVhygro-F-Ezh2 wild-type or F667I (Addgene 24926 and 24927, a gift from Kai Ge) (Wang et al., 2010). For all constructs the N-terminal tag (HA or FLAG) was removed during cloning. The EED W364A mutant was generated by site-directed mutagenesis of wildtype EED that had been subcloned into pcDNA3. Following sequence verification, EED W364A was subcloned into pHRSIN-P_{SFFV}-eGFP-P_{PGK}-Puro. All plasmids were verified by Sanger sequencing analysis through the Australian Genome Research Facility (Melbourne, Victoria). An ovalbumin (OVA) expressing retroviral vector was generated by PCR amplification of full-length chicken OVA from pcDNA3-OVA (Addgene 64599, a gift from Sandra Diebold & Martin Zenke) (Diebold et al., 2001) and cloned into MSCV-IRES-mCherry via EcoRI and NotI sites.

CRISPR sgRNA Library

The screen was performed using the Bassik Human CRISPR KO Library (a gift from Michael Bassik, Addgene 101296-101934). This 10-sgRNA-per-gene CRISPR/Cas9 deletion library was designed to target all ~20,500 protein-coding human genes. The library contains two distinct classes of negative control gRNAs: non-targeting control sgRNA with no binding sites in the genome and

safe-targeting sgRNA targeting genomic locations with no annotated function. Further details are described in Morgens DW et al (Morgens et al., 2017). Library sgRNAs are expressed in the pMCB320 lentiviral sgRNA expression vector, which encodes puromycin and mCherry selection markers.

CRISPR Screen

K-562 cells were transduced with a lentiviral vector encoding Cas9 and selected with blasticidin. For the screen, 10^8 K-562 Cas9 cells were infected with the pooled lentiviral genome-wide sgRNA library at a multiplicity of infection of 0.3. The percent of cells infected was determined by flow-cytometry based evaluation of mCherry positive (sgRNA expressing) cells 72 hr following transduction. Infected cells were selected with 1 μg/mL puromycin for 72 hr, commencing 48 hr after transduction. Rare MHC-I positive cells were enriched by three rounds of FACS sorting at day 7, day 15 and day 30 following transduction with the sgRNA library. For the first and second sorts, cells were stained with APC-conjugated anti-human HLA-A,B,C antibody (W6/32, BioLegend) for 15 minutes on ice and washed with PBS prior to sorting for mCherry positive (sgRNA expressing) MHC-I positive cells on a BD Influx cell sorter. For the third sort, two parallel sorts were performed using either a pan-HLA-A, B, C-specific antibody or using an HLA-B specific antibody (REA143, Miltenyi Biotec). Genomic DNA was extracted (Puregene Core Kit A, Qiagen) from both the sorted cells and an unselected pool of mutagenized cells grown for the same amount of time. sgRNA sequences were amplified by two rounds of PCR, with the second round primers containing adaptors for Illumina sequencing. Samples were sequenced with single-end 50 bp reads on an Illumina HiSeq. The sequence reads were trimmed to remove the constant portion of the sgRNA sequences with fastx clipper (http://hannonlab.cshl.edu/fastx_toolkit/), then mapped to the reference sgRNA library with bowtie2. After filtering to remove multi-aligning reads, the read counts were computed for each sgRNA. The RSA algorithm (Konig et al., 2007) was used to rank the genes for which targeting sgRNA were significantly enriched in the sorted populations compared to the control unsorted populations grown in parallel.

CRISPR/Cas9-Mediated Gene Disruption and Generation of Knockout Clones

Single guide RNA (sgRNA) oligonucleotides (Sigma-Aldrich) were cloned into lentiviral expression vector pKLV-U6gRNA(BbsI)-$_{PGK}$puro$_{2A}$BFP as described (Addgene 50946, a gift from Kosuke Yusa). For CRISPR/Cas9 mediated gene disruption, cells were first transduced with the Cas9 expression vector pHRSIN-P_{SFFV}-Cas9-P_{PGK}-Blasticidin (Burr et al., 2017) or FUCas9Cherry (a gift from Marco Herold, Addgene 70182), and selected with blasticidin or sorted for mCherry expression respectively. To generate polyclonal populations with targeted gene disruption, cells were subsequently transduced with pKLV-gRNA-$_{PGK}$puro$_{2A}$BFP encoding either gene specific sgRNAs or with a control sgRNA targeting a 'safe' genomic location with no annotated function (Morgens et al., 2017). Efficient functional CRISPR/Cas9 mediated gene disruption of *EED* and *EZH2* was confirmed by immunoblot for H3K27me3 and of *IRF1*, *STAT1* and *RFX5* was confirmed by loss of IFN-γ-induced MHC-I upregulation. To generate *EED* and *EZH2* KO clones used in complementation experiments, sgRNAs targeting either *EED* or *EZH2* were transiently expressed using lentiviral infection coupled with treatment with the integrase inhibitor raltegravir (Selleck Chemicals), which effectively inhibits lentiviral integration. This method allows highly efficient CRISPR/Cas9-mediated gene disruption following transient sgRNA expression in non-adherent cells. Cas9-expressing K-562 or mSCLC cells were transduced with pKLV-gRNA-$_{PGK}$puro$_{2A}$BFP expressing *EED* or *EZH2* specific sgRNA. 2 nM raltegravir was added during transduction and continued for 10 days. Effective inhibition of integration was confirmed by gradual loss of BFP expression from day 3 following infection. Control cells were transduced with a control sgRNA targeting a 'safe' genomic location and treated under the same conditions. Cells were selected with puromycin 2 μg/mL for 24 hr, beginning 24 hr following transduction, and subsequently single cell sorted into 96-well plates using a BD FACSAria Fusion flow cytometer. Clones were screened by immunoblot for H3K27me3 and EZH2 and FACS for cell surface MHC-I. Identified *EED*/*EZH2* KO clones were verified to be BFP negative and sensitive to puromycin to exclude integration of the lentiviral sgRNA vector. To generate *Ezh2* KO mSCLC (RP-116) clones lacking Cas9 expression for *in vivo* experiments, Cas9 and the sgRNAs were both transiently expressed as described above and single cell clones established. For complementation experiments, selected *EED* or *EZH2* KO K-562 or mSCLC RP-116 clones were infected with pHRSIN-P_{PGK}-Puromycin encoding wildtype or mutant EED or EZH2 and selected with puromycin.

Lentiviral Production and Transduction

Lentivirus was produced by triple transfection of HEK-293ET cells with a lentiviral transfer vector, and the packaging plasmids psPAX2 and pMD.G at a 0.5:0.35:0.15 ratio. Transfection was performed using JetPEI reagent as recommended by the manufacturer. The viral supernatant was collected 60 hr following transfection, filtered through a 0.45 μm filter, and added to target cells.

Flow Cytometry

Cells were washed in PBS and stained on ice for 20-30 min in PBS plus 2% FCS. After washing in PBS/2% FCS, samples were either resuspended in PBS/2% FCS or fixed in 1% paraformaldehyde (PFA). Data were acquired on a BD LSR Fortessa or BD FACSymphony and analysed in FlowJo.

Immunoblotting

Cells were lysed in 1% SDS in 100 mM Tris-HCl pH 8.0 with Roche complete EDTA-free protease inhibitor at room temperature. DNA was fragmented either by sonication or adding Benzonase (Sigma) 1:100. Lysates were heated to 70°C in SDS sample buffer with

50 mM DTT for 10 min, separated by SDS-PAGE, and transferred to PVDF membrane (Millipore). Membranes were blocked in 5% milk in TBS + 0.1% Tween-20, probed with the indicated antibodies, and reactive bands visualised using West Pico (Thermo Fisher Scientific).

Fluorescent Multiplex Immunohistochemistry

4 μm thick formalin-fixed paraffin-embedded sections of mSCLC tumors grown in BALB/c or C57BL/6 mice were baked at 60°C for 30 minutes prior to being loaded onto the Leica BOND RX for automated fluorescent multiplex immunohistochemistry. The first heat induced antigen retrieval was performed with EDTA pH 9. Tissue sections were incubated with a protein block supplied by the manufacturer within the Opal immunostaining kit (NEL811001KT, Perkin Elmer) for 10 min and then immersed in 3% hydrogen peroxide for 10 min. The first primary antibody, rabbit α-mouse NCAM (95153, Abcam), 1:400, was applied to tissue sections and incubated at room temperature for 1 hour followed by a 10 minute incubation with an α-rabbit HRP-conjugated secondary antibody (NEF812001EA, Perkin Elmer), 1:1000. This was followed by 10 min incubation with tyramide-conjugated fluorophore working solution, consisting of fluorophore diluted to 1:150 in the amplification diluent supplied by Perkin Elmer. This process was repeated for each of the following antibodies (with the exception of the hydrogen peroxide block which was only performed once). Citrate buffer pH 6 was used for the second heat induced antigen retrieval followed by incubation with monoclonal rabbit α-CD3 antibody (16669, Abcam), 1:100. EDTA pH 9 was used for the final heat induced antigen retrieval followed by incubation with rabbit α-mouse CD8-alpha antibody (209775, Abcam), 1:2000. DAPI was then applied to tissue sections for 3 minutes at room temperature.

Image acquisition of stained tissue sections was performed on the Vectra 3.0 Multispectral Imaging Platform (Perkin Elmer). Spectral un-mixing of fluorophores was performed using the software, inForm (Perkin Elmer), whilst digital analysis of tissue sections was performed using the software, HALO v2.2.1870 (Indica Labs, USA). The analysis algorithm developed for this study used the Highplex FL v.2.0 module, specific for analysis of multiplex immunohistochemical stained tissue sections.

Isolation and In Vitro Activation of $CD8^+$ T Cells

OT-I TCR Transgenic mice were purchased from the Walter and Eliza Hall Institute (Melbourne, Australia). Splenocytes were harvested from the spleen of OT-I mice and stimulated by incubation with 300 ng/mL of SIINFEKL (OVA peptide) (Sigma-Aldrich) for 24 hr to expand $CD8^+$ OT-I T cells. After washing to remove the peptide, cells were cultured in media supplemented with IL-2 at 100 U/mL (Abcam) for an additional 2 days. T cells were subsequently isolated from the mononuclear layer using Ficoll separation and cultured with IL-2 for an additional 3 days before use in co-culture assays. Successful enrichment of a $CD8^+$ T cell population was confirmed by flow cytometry staining for CD45, CD3 and CD8.

T-cell Cytotoxicity Assays

Peptide Pulsing Assay

mSCLC cells were pretreated as indicated with 3 μM EPZ-011989 or ethanol control for 10 days, and then incubated with or without IFN-γ 10 ng/mL. After thorough washing to remove the inhibitor and IFN-γ, cells were pulsed with 1 ng/mL of SIINFEKL (OVA peptide) at 37°C for 2 hours, which binds to cell surface MHC-I (H-2Kb). Cells were subsequently washed to remove unbound peptide and then mixed at a 1:1 ratio with control mCherry positive untreated mSCLC cells (expressing an MSCV mCherry control vector). Cell mixtures were individually plated in 96-well plates and T cells added at a range of different effector:target ratios. Assays were set up in triplicate wells for each co-culture condition. After incubation at 37°C for 24 hr, samples were analyzed by flow cytometry to determine the percent killing of peptide-pulsed cells for each pretreatment condition. This was determined for each effector:target ratio by comparing the relative percent of target (mCherry negative) tumor cells remaining in the wells that received T cells compared to the wells that did not receive T cells. Tumor cells were identified as the viable (Fixable Violet negative), CD45 negative population. As an additional control to evaluate whether tumor cell killing was MHC-I antigen-specific, assays were set up as described above but omitting the peptide pulsing step to determine whether tumor cell killing was dependent on the presence of the peptide specifically recognized by the OT-I T-cell receptor.

OVA Peptide Processing and Presentation Assay

Ovalbumin (OVA) expressing mSCLC cells (mSCLC) were generated by infection with MSCV-OVA-mCherry. mSCLC-OVA cells were pretreated as previously described with 3 μM EPZ-011989 or ethanol control for 10 days, and then incubated with or without 10 ng/mL IFN-γ. After thorough washing to remove the inhibitor and IFN-γ, pretreated mSCLC-OVA cells (mCherry positive) were mixed with untreated parental mSCLC cells (mCherry negative) at a 1:1 ratio. Cell mixtures were analyzed by flow cytometry at baseline to determine the percent of mSCLC-OVA (mCherry positive) cells. Cell mixtures were individually plated in 96-well plates and T cells added at a range of different effector:target ratios. Assays were set up in triplicate wells for each co-culture condition. After incubation at 37°C for the indicated times, co-cultures were analyzed by flow cytometry as described above. The relative depletion of mSCLC-OVA cells in the presence or absence of T cells was calculated by comparing the relative percent of mSCLC-OVA (mCherry positive) cells present at each timepoint compared to the percent of mSCLC-OVA (mCherry positive) cells present at baseline.

Measurement of T-cell Cytokine Secretion

$1x10^5$ mSCLC or mSCLC-OVA cells, pretreated as indicated in the figure legends, were co-cultured with 2×10^5 OT-I T cells in 96-well plates. Co-cultures were performed in triplicate for each condition. The cell culture media was collected after 24 hours of co-culture. The levels of TNFα and IFN-γ were measured with the BD cytometric bead array Mouse Th1/Th2 cytokine kit and analyzed by flow cytometry.

qRT–PCR

mRNA was prepared with a Qiagen RNeasy kit, and cDNA synthesis was performed with a SuperScript VILO kit (Life Technologies), per the manufacturers' instructions. Quantitative PCR analysis was performed on an Applied Biosystems StepOnePlus System with SYBR Green reagents. All samples were assayed in triplicate. Relative expression levels were determined with the ΔCt method and normalized to *GAPDH* and/or *ACTB*.

RNA-Sequencing

RNA was extracted using the Qiagen RNeasy kit. RNA concentration was quantified using a Qubit Fluorometer (Thermo Fisher Scientific). Libraries were prepared using QuantSeq 3′ mRNA-seq Library Prep kit (Lexigen). Libraries were sequenced on the NextSeq500 using 75 bp single end chemistry.

RNA-Sequencing Analysis

Bcl2fastq (Illumina) was used to perform sample demultiplexing and to convert BCL files generated from the sequencing instrument into FastQ files. Reads were aligned to the human genome (G1k V37), or mouse genome (MM10) using HiSAT2 and reads were assigned to genes using htseq-count. Differential expression was calculated using either limma, voom or DESeq2. Genes with a false discovery rate corrected for multiple testing using the method of Benjamini and Hochberg below 0.05 and a fold change greater than 1.5 were considered significantly differentially expressed. Heatmaps were generated in R using pheatmap.

Chromatin Immunoprecipitation (ChIP)

Chromatin immunoprecipitation was performed as described previously (Gilan et al., 2016). Cells were cross-linked with 1% formaldehyde for 15 min at room temperature, and cross-linking was stopped by the addition of 0.125 M glycine. Cells were washed in ice cold PBS and either flash frozen and stored at -80°C or directly lysed in 1% SDS, 10 mM EDTA, 50 mM Tris-HCl, pH 8.0, and Roche complete EDTA-free protease inhibitor. For normalization of H3K27me3, H3K4me3 and H3K27ac ChIP, Drosophila S2 cells cross-linked and lysed under the same conditions were mixed with the target cell lysates at a ratio of 1:4 (Drosophila S2 cell number:target cell number). Lysates were sonicated in a Covaris ultrasonicator to achieve a mean DNA fragment size of 500 bp. Samples were diluted 1:10 in modified RIPA buffer (1% Triton X-100, 0.1% deoxycholate, 90 mM NaCl, 10 mM Tris-HCl pH 8.0, and protease inhibitors) and incubated rotating with antibody for a minimum of 12 hr at 4°C. Antibodies used were rabbit anti-H3K27me3 (C37B11, Cell Signaling), rabbit anti-H3K27ac (ab4729, Abcam), rabbit anti-H3K4me3 (ab8580, Abcam) and rabbit α-IRF-1 (D5E4, Cell Signaling), 8 to 10 μg per sample. Protein A Dynabeads (Life Technologies) were added to bind the antibody and associated chromatin and samples incubated for 2 hr at 4°C. After washing twice with low salt buffer (0.1% SDS, 1% Triton X-100, 20 mM Tris-HCl pH8.0, 2 mM EDTA, 150 mM NaCl), once with high salt buffer (0.1% SDS, 1% Triton X-100, 20 mM Tris-HCl pH 8.0, 2 mM EDTA, 500 mM NaCl) and once with TE (10 mM Tris [pH 8.0], 1 mM EDTA), samples were eluted from the beads for 30 min at 65°C in elution buffer (1% SDS, 0.1 M $NaHCO_3$). Eluates were reverse cross-linked overnight by heating at 65°C with RNase A and 0.2 M NaCl and then purified using the QIAquick PCR purification kit (Qiagen). DNA was analyzed by qPCR on an Applied Biosystems StepOnePlus System with SYBR Green reagents. Sequencing libraries were prepared from eluted DNA using Rubicon ThruPLEX DNA-seq kit or NEB Next ChIP-seq library preparation kit. Libraries were size selected between 200-700 bps and sequenced on the NextSeq500 using 75 bp single-end chemistry.

ChIP-Sequencing Analysis

Bcl2fastq (Illumina) was used to perform sample demultiplexing and to convert BCL files generated from the sequencing instrument into FastQ files. Reads were aligned to the human genome (GRCh37) or mouse genome (GRCm38), combined with fly genome (BDGP5) or a modified Tasmanian devil genome (Devil_ref v7.0) with BWA-mem. The scaffold GL857536.1 encoding the *TAP2* gene was replaced with a BAC clone (accession FQ482140) encoding the whole TAP cluster of genes (Cheng Y 2012. BMC Genomics, Antigen-presenting genes and genomic copy number variations in Tasmanian devil MHC), in the Tasmanian devil genome. Peak calling was performed with MACS2 with default parameters, and deepTools was used for normalization. Genome-browser images of ChIP–seq data was generated by converting files to TDF files with igvtools and viewing in IGV. ChIP-seq coverage across selected genomic regions was calculated with BEDtools57. ChIP heatmap was created using data generated by ngsplot, the transcriptional start site (tss) is 2kb upstream of the gene start site, genebody was separated into 5 even bins, and the transcriptional end site (tes) is the end of the gene plus 2kb downstream. Chromatin state analysis was performed using ChromHMM with either bed or bam files with H3K27me3, H3K27ac, H3K4me3 and H3K36me3 ChIP-seq data for each cell type, specifying a 9 state parameter. Active (tss) was assigned to high H3K27ac and H3K4me3 at 500 bp around the tss, bivalent tss was assigned to high H3K27me3 and H3K4me3 at 500 bp around the tss. Strong transcription was assigned to

high H3K36me3 signal, weak transcription was assigned to low H3K36me3 signal, repressed polycomb was assigned to high H3K27me3 signal, and inactive genes was assigned to no signal along the genebody. The heatmap for the chromatin state was generated in R.

QUANTIFICATION AND STATISTICAL ANALYSIS

Statistical analysis was carried out using GraphPad Prism 8. Details of statistical analysis performed in Methods Details of STAR Methods and in the figure legends. Data were reported as mean ± SEM or independent replicates shown as individual data points, as indicated in the figure legends. Significance was defined as $p < 0.05$.

DATA AND CODE AVAILABILITY

Sequencing data has been deposited into the sequence read archive, hosted by the National Centre for Biotechnology Information. The accession number for the sequencing data reported in this paper is NCBI sequence read archive: PRJNA527170. Unprocessed data (immunoblot scans) are available at https://doi.org/10.17632/yf4vcxy469.1.

Cancer Cell, Volume *36*

Supplemental Information

An Evolutionarily Conserved Function of Polycomb Silences the MHC Class I Antigen Presentation Pathway and Enables Immune Evasion in Cancer

Marian L. Burr, Christina E. Sparbier, Kah Lok Chan, Yih-Chih Chan, Ariena Kersbergen, Enid Y.N. Lam, Elizabeth Azidis-Yates, Dane Vassiliadis, Charles C. Bell, Omer Gilan, Susan Jackson, Lavinia Tan, Stephen Q. Wong, Sebastian Hollizeck, Ewa M. Michalak, Hannah V. Siddle, Michael T. McCabe, Rab K. Prinjha, Glen R. Guerra, Benjamin J. Solomon, Shahneen Sandhu, Sarah-Jane Dawson, Paul A. Beavis, Richard W. Tothill, Carleen Cullinane, Paul J. Lehner, Kate D. Sutherland, and Mark A. Dawson

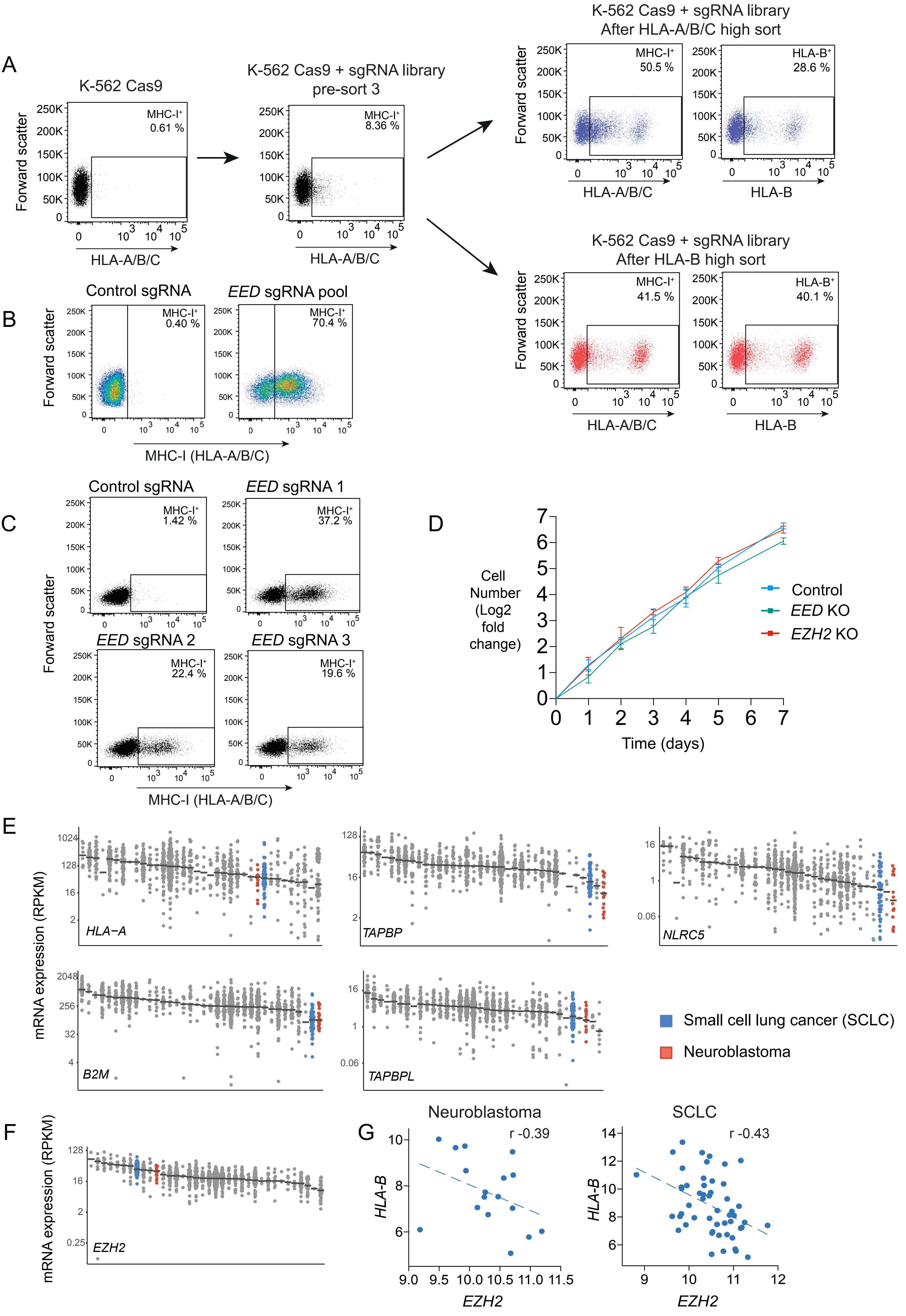
A
K-562 Cas9
MHC-I+ 0.61 %
K-562 Cas9 + sgRNA library pre-sort 3
MHC-I+ 8.36 %
K-562 Cas9 + sgRNA library After HLA-A/B/C high sort
MHC-I+ 50.5 %
HLA-B+ 28.6 %
K-562 Cas9 + sgRNA library After HLA-B high sort
MHC-I+ 41.5 %
HLA-B+ 40.1 %
Forward scatter
HLA-A/B/C
HLA-B
B
Control sgRNA
MHC-I+ 0.40 %
EED sgRNA pool
MHC-I+ 70.4 %
MHC-I (HLA-A/B/C)
C
Control sgRNA
MHC-I+ 1.42 %
EED sgRNA 1
MHC-I+ 37.2 %
EED sgRNA 2
MHC-I+ 22.4 %
EED sgRNA 3
MHC-I+ 19.6 %
MHC-I (HLA-A/B/C)
D
Cell Number (Log2 fold change)
Time (days)
Control
EED KO
EZH2 KO
E
mRNA expression (RPKM)
HLA−A
TAPBP
NLRC5
B2M
TAPBPL
Small cell lung cancer (SCLC)
Neuroblastoma
F
mRNA expression (RPKM)
EZH2
G
Neuroblastoma
r -0.39
SCLC
r -0.43
HLA-B
EZH2

Figure S1. Related to Figure 1. PRC2 components maintain silencing of MHC-I expression in MHC-I deficient cancers. (A) K-562 cells stably expressing Cas9 were mutagenized by infection with a pooled lentiviral sgRNA library and rare MHC-I high cells were enriched by 3 successive rounds of FACS sorting for mCherry positive (containing sgRNA vector) MHC-I positive cells. FACS plots show MHC-I expression in: unsorted K-562 Cas9 cells (left), K-562 Cas9 cells transduced with the CRISPR sgRNA library before the third sort enrichment (middle), and the same cells after sort enrichment with either a pan-HLA-A,B,C antibody (top right panels) or with an HLA-B specific antibody (bottom right panels). (B) Cell surface MHC-I in K-562 Cas9 cells transduced with a pool of 3 sgRNAs targeting *EED* or a control sgRNA. (C) Cell surface MHC-I in K-562 Cas9 cells transduced with individual sgRNAs targeting *EED* or a control sgRNA. (D) *In vitro* proliferation of *EED* and *EZH2* KO K-562 clones compared to control parental cells. Graph shows mean and SEM log2 fold change in cell number compared to baseline from 4 experiments. (E and F) mRNA expression of MHC-I genes (E) and *EZH2* (F) in 920 cell lines in Cancer Cell Line Encyclopedia. Each dot represents an individual cancer cell line, clustered by tumor type (RPKM log2 scale). Black line indicates median. (G) Correlation plot showing mRNA expression data for *EZH2* and *HLA-B* in neuroblastoma and SCLC from Cancer Cell Line Encyclopedia (accessed via http://www.cbioportal.org/). Pearson's correlation coefficient (r).

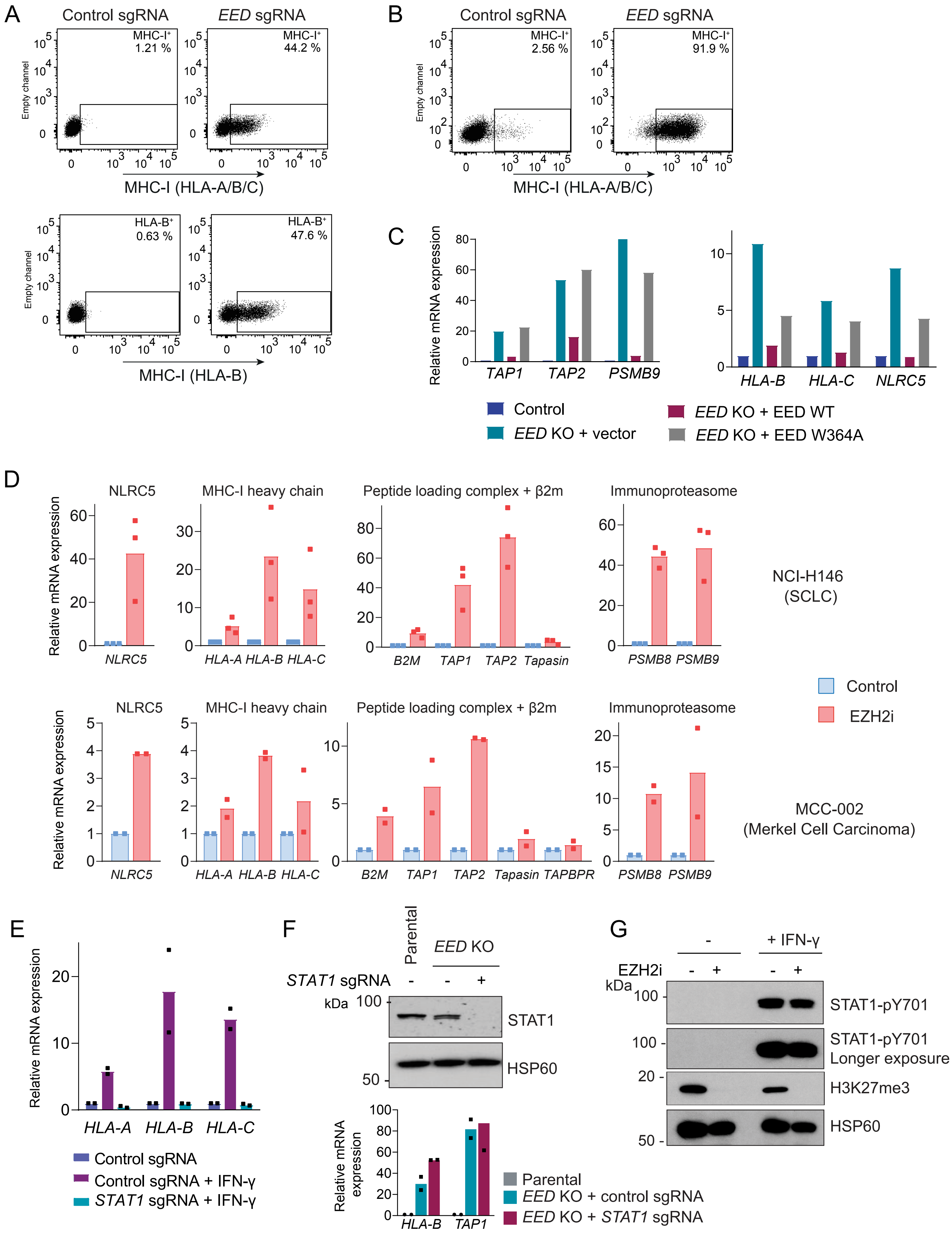

A
Control sgRNA
EED sgRNA
MHC-I+ 1.21 %
MHC-I+ 44.2 %
Empty channel
MHC-I (HLA-A/B/C)
HLA-B+ 0.63 %
HLA-B+ 47.6 %
MHC-I (HLA-B)
B
Control sgRNA
EED sgRNA
MHC-I+ 2.56 %
MHC-I+ 91.9 %
MHC-I (HLA-A/B/C)
C
Relative mRNA expression
TAP1
TAP2
PSMB9
HLA-B
HLA-C
NLRC5
Control
EED KO + vector
EED KO + EED WT
EED KO + EED W364A
D
NLRC5
MHC-I heavy chain
Peptide loading complex + β2m
Immunoproteasome
Relative mRNA expression
NLRC5
HLA-A HLA-B HLA-C
B2M TAP1 TAP2 Tapasin
PSMB8 PSMB9
NCI-H146 (SCLC)
Control
EZH2i
B2M TAP1 TAP2 Tapasin TAPBPR
MCC-002 (Merkel Cell Carcinoma)
E
Relative mRNA expression
HLA-A HLA-B HLA-C
Control sgRNA
Control sgRNA + IFN-γ
STAT1 sgRNA + IFN-γ
F
Parental
EED KO
STAT1 sgRNA
kDa
STAT1
HSP60
Relative mRNA expression
HLA-B TAP1
Parental
EED KO + control sgRNA
EED KO + STAT1 sgRNA
G
+ IFN-γ
EZH2i
kDa
STAT1-pY701
STAT1-pY701 Longer exposure
H3K27me3
HSP60

Figure S2. Related to Figure 2. Genetic disruption or pharmacological inhibition of PRC2 restores MHC-I expression in MHC-I low cancers. (A and B) Cell surface MHC-I in Cas9 expressing human neuroblastoma (Kelly) (A) or SCLC (NCI-H146) (B) following transduction with a pool of 2 sgRNAs targeting *EED* compared to control sgRNA. (C) qRT-PCR analysis of MHC-I gene expression in *EED* KO K-562 cells transduced with lentiviral vectors encoding wild-type EED, EED W364A or GFP (vector). Expression of MHC-I APP genes relative to control parental K-562 Cas9 cells. Bars show mean of technical triplicates from a representative experiment. (D) qRT-PCR analysis of MHC-I gene expression in cells treated with EZH2 inhibitor (EZH2i), GSK-503 (5 μM) in NCI-H146 and EPZ-011989 (3 μM) in MCC-002, compared to DMSO or ethanol (ETOH) treated control. Analyses performed after 10 days of treatment. (E) qRT-PCR analysis of K-562 Cas9 cells expressing *STAT1* targeting or control sgRNA treated with or without IFN-γ 10 ng/ml for 24 hr. (F) *EED* knockout K-562 Cas9 cells were transduced with control or *STAT1* targeting sgRNA and analyzed by immunoblot and qRT-PCR compared to parental K-562 Cas9 cells. (G) Immunoblot analysis of K-562 cells treated with EPZ-011989 3 μM or ETOH control for 10 days and pulsed with or without IFN-γ 10 ng/ml for 24 hr. For D, E and F, bars indicate the mean fold change from independent experiments and points denote the mean of technical triplicates from individual experiments.

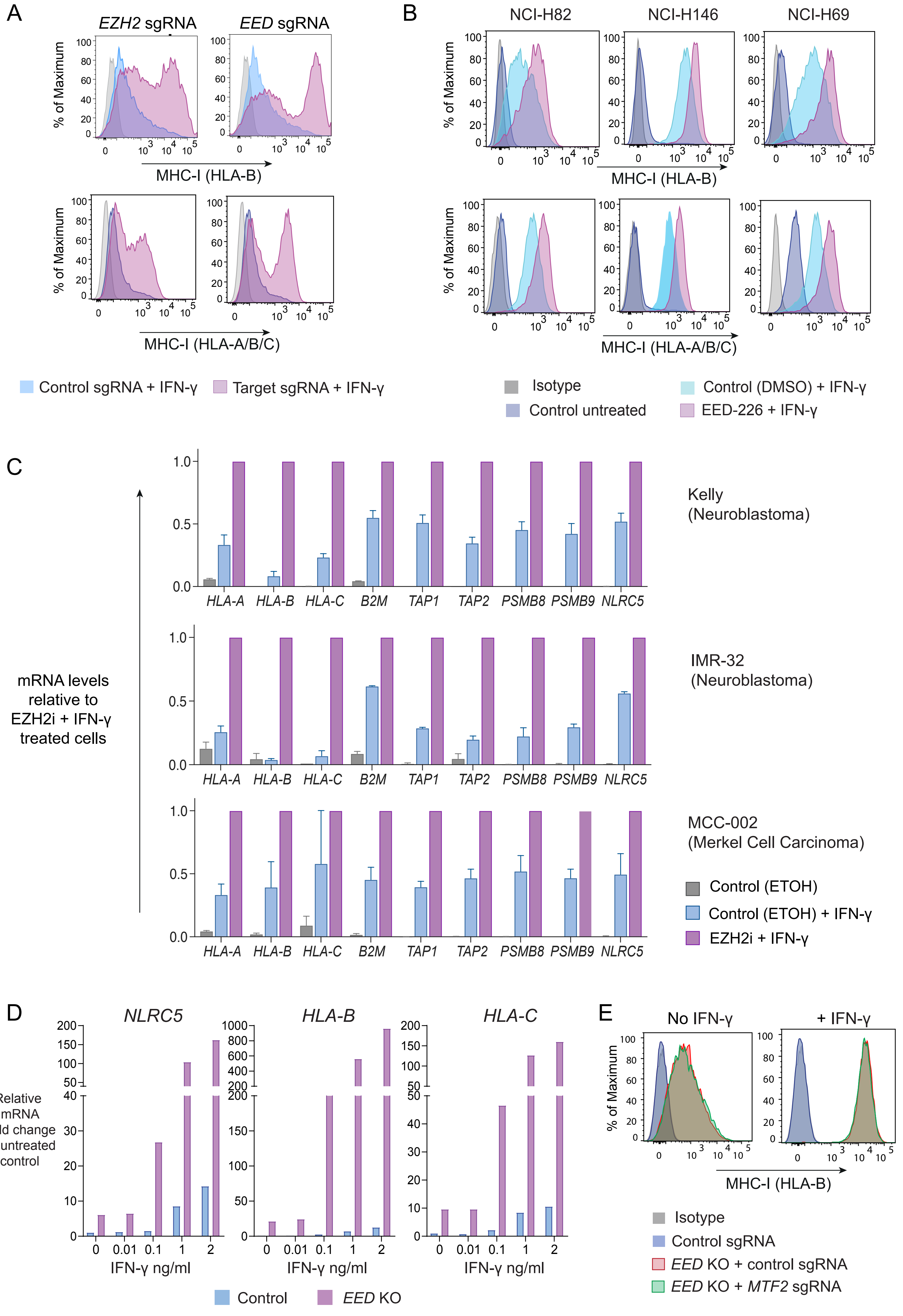

A
EZH2 sgRNA
EED sgRNA
% of Maximum
MHC-I (HLA-B)
MHC-I (HLA-A/B/C)
Control sgRNA + IFN-γ
Target sgRNA + IFN-γ
B
NCI-H82
NCI-H146
NCI-H69
% of Maximum
MHC-I (HLA-B)
MHC-I (HLA-A/B/C)
Isotype
Control untreated
Control (DMSO) + IFN-γ
EED-226 + IFN-γ
C
mRNA levels relative to EZH2i + IFN-γ treated cells
HLA-A HLA-B HLA-C B2M TAP1 TAP2 PSMB8 PSMB9 NLRC5
Kelly (Neuroblastoma)
IMR-32 (Neuroblastoma)
MCC-002 (Merkel Cell Carcinoma)
Control (ETOH)
Control (ETOH) + IFN-γ
EZH2i + IFN-γ
D
NLRC5
HLA-B
HLA-C
Relative mRNA Fold change vs untreated control
IFN-γ ng/ml
Control
EED KO
E
No IFN-γ
+ IFN-γ
% of Maximum
MHC-I (HLA-B)
Isotype
Control sgRNA
EED KO + control sgRNA
EED KO + MTF2 sgRNA

Figure S3. Related to Figure 3. Loss of PRC2 function augments IFN-γ induced MHC-I expression in MHC-I low cancers. (A) Cell surface MHC-I in K-562 Cas9 cells transduced with sgRNA targeting *EED* or *EZH2*, or control sgRNA, and pulsed with IFN-γ 10 ng/ml for 24 hr. (B) Cell surface MHC-I in human SCLC incubated with EED inhibitor (EED-226 5 μM) or DMSO control for 10 days and pulsed with IFN-γ 10 ng/ml for 24 hr. (C) qRT-PCR analysis in the indicated human cancer cells treated with EPZ-011989 3 μM or ETOH control for 10 days and pulsed with IFN-γ 10 ng/ml for 24 hr. mRNA levels are expressed proportional to expression in cells treated with combination of EZH2i and IFN-γ. Graphs show mean ± SEM from independent experiments. (D) qRT-PCR analysis of MHC-I gene expression in *EED* KO or parental control K-562 cells incubated with the indicated concentrations of IFN-γ for 24 hr. (E) Cell surface HLA-B in *EED* knockout K-562 Cas9 cells expressing control sgRNA or two *MTF2*-specific sgRNAs. In right panel, cells were incubated with IFN-γ 10 ng/ml for 24 hr.

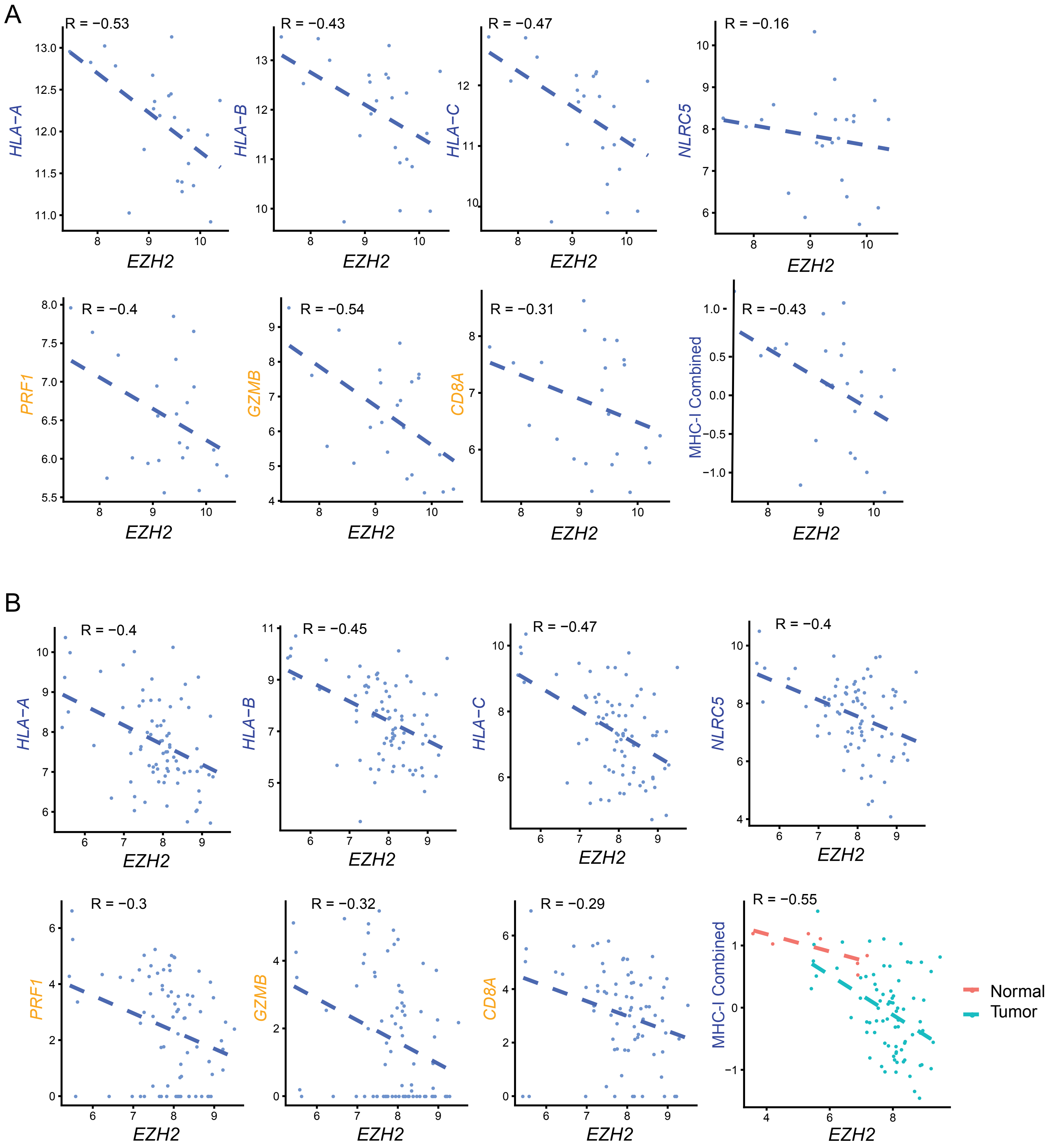
A
R = −0.53
HLA−A
EZH2
R = −0.43
HLA−B
EZH2
R = −0.47
HLA−C
EZH2
R = −0.16
NLRC5
EZH2
R = −0.4
PRF1
EZH2
R = −0.54
GZMB
EZH2
R = −0.31
CD8A
EZH2
R = −0.43
MHC-I Combined
EZH2
B
R = −0.4
HLA−A
EZH2
R = −0.45
HLA−B
EZH2
R = −0.47
HLA−C
EZH2
R = −0.4
NLRC5
EZH2
R = −0.3
PRF1
EZH2
R = −0.32
GZMB
EZH2
R = −0.29
CD8A
EZH2
R = −0.55
MHC-I Combined
EZH2
Normal
Tumor

Figure S4. Related to Figures 4 and 6. *EZH2* expression is negatively correlated with expression of MHC-I genes and markers of $CD8^+$ T cells in primary human SCLC tumor samples. (A and B) Correlation plots show expression of MHC-I genes, T cell genes and *EZH2* in 23 primary SCLC tumor samples analyzed using Affymetrix Human Genome Array (A) and 79 primary SCLC tumors and 7 normal lung tissue samples analyzed by RNA-sequencing (B). Plots show normalized log2 mRNA expression of the indicated genes and the Pearson's correlation coefficient (R). The combined MHC-I score represents the mean normalized log2 expression of multiple MHC-I genes (*HLA-A, HLA-B, HLA-C, TAP1, TAP2, TAPBP, B2M, NLRC5, PSMB8, PSMB9, ERAP1, ERAP2*). MHC-I genes are indicated in blue and $CD8^+$ T cell genes in orange. *PRF1* (perforin); *GZMB* (granzyme B). Data in A are from GSE43346 (Sato et al, 2013) and data in B are from GSE60052 (Jiang et al, 2016).

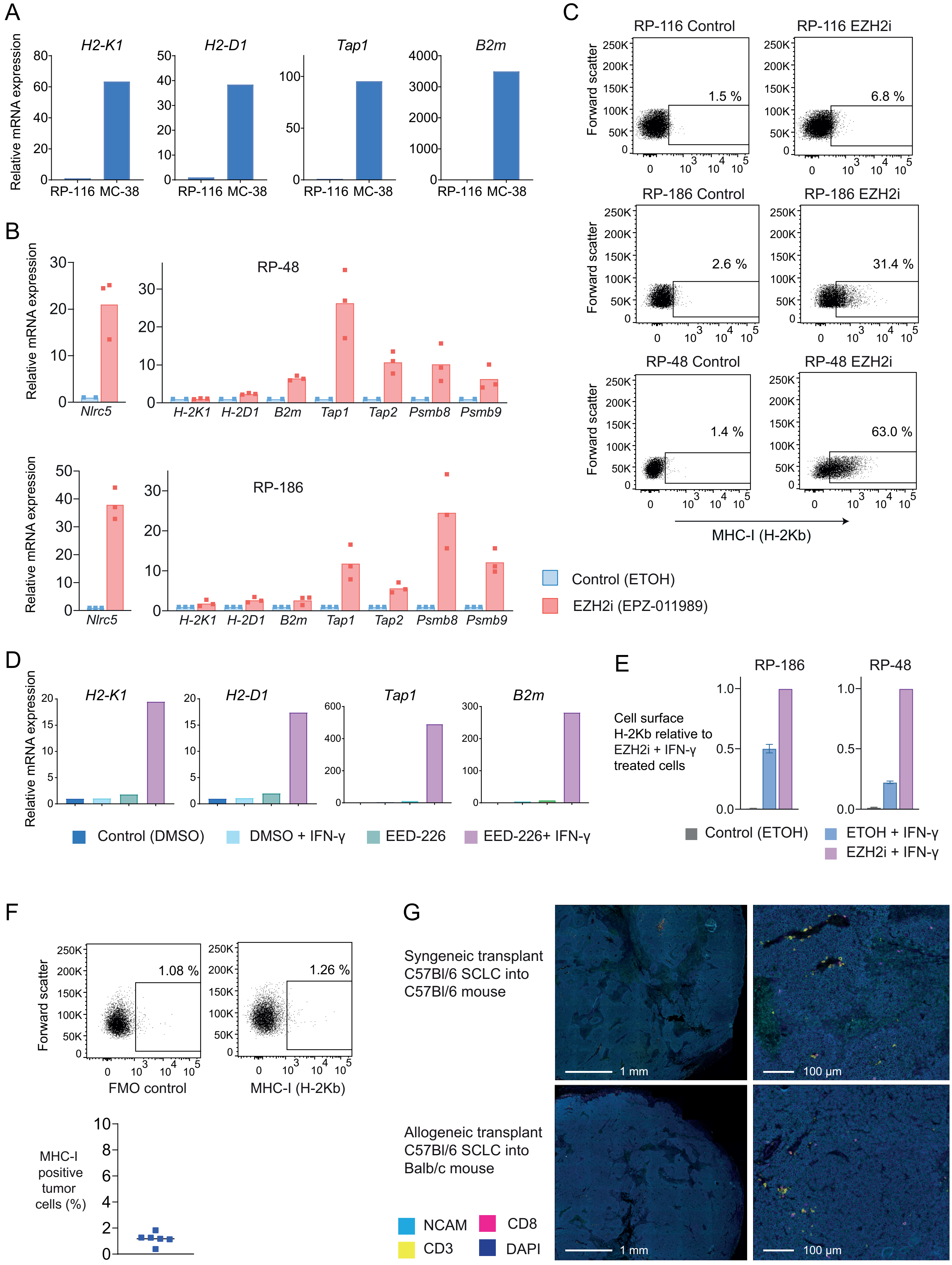

A
Relative mRNA expression
H2-K1
H2-D1
Tap1
B2m
RP-116 MC-38
B
Relative mRNA expression
RP-48
RP-186
Nlrc5
H-2K1 H-2D1 B2m Tap1 Tap2 Psmb8 Psmb9
Control (ETOH)
EZH2i (EPZ-011989)
C
RP-116 Control
RP-116 EZH2i
RP-186 Control
RP-186 EZH2i
RP-48 Control
RP-48 EZH2i
Forward scatter
1.5 %
6.8 %
2.6 %
31.4 %
1.4 %
63.0 %
MHC-I (H-2Kb)
D
Relative mRNA expression
H2-K1
H2-D1
Tap1
B2m
Control (DMSO)
DMSO + IFN-γ
EED-226
EED-226+ IFN-γ
E
RP-186
RP-48
Cell surface H-2Kb relative to EZH2i + IFN-γ treated cells
Control (ETOH)
ETOH + IFN-γ
EZH2i + IFN-γ
F
Forward scatter
1.08 %
1.26 %
FMO control
MHC-I (H-2Kb)
MHC-I positive tumor cells (%)
G
Syngeneic transplant C57Bl/6 SCLC into C57Bl/6 mouse
Allogeneic transplant C57Bl/6 SCLC into Balb/c mouse
1 mm
100 µm
NCAM
CD8
CD3
DAPI

Figure S5. Related to Figures 4 and 6. PRC2 silences MHC-I expression in mouse *Trp53⁻ Rb1⁻* SCLC tumors. (A) qRT-PCR analysis of MHC-I gene expression in mouse SCLC RP-116 cells and mouse colorectal carcinoma MC-38 cells. Bars depict mean fold difference in mRNA expression in MC-38 relative to RP-116 from a representative experiment**.** (B and C) qRT-PCR (B) and flow cytometry analysis (C) of MHC-I expression in the indicated mSCLC cells treated with EZH2i (EPZ-011989 3 μM) or ETOH control. Analysis after 10 days of treatment. For B, bars show mean fold change in mRNA expression compared to control from 3 experiments and individual points denote the mean of technical triplicates from each experiment. (D) qRT-PCR analysis in RP-116 cells treated with EED-226 5 μM or DMSO control for 10 days and pulsed with or without IFN-γ 10 ng/ml for 24 hr. (E) Flow cytometry analysis of cell surface MHC-I (H-2Kb) in mSCLC RP-186 and RP-48. Cells treated with EZH2i (EPZ-011989 3 μM) or ETOH control for 10 days and pulsed with IFN-γ 10 ng/ml for 24 hr. Relative MFI is expressed proportional to MFI of cells treated with combination of EZH2i and IFN-γ. Mean ± SEM of 3 experiments. (F) Cell surface MHC-I expression in mouse SCLC (RP-116) tumors harvested and digested following subcutaneous transplant into C57Bl/6 mice. Tumor cells were identified as the viable cells negative for CD45, CD31 and Ter199 and MHC-I positive cells were gated with reference to fluorescence minus one (FMO) control. Graph shows results from 6 mice. Horizontal black line indicates mean. (G) Multiplex immunohistochemistry of mSCLC RP-116 tumors transplanted into C57Bl/6 or Balb/c mice. Tumors were harvested and fixed at a size of 500 mm^3. NCAM marks SCLC tumor cells and T cells were stained with CD3 and CD8. Representative images of intratumoral staining are shown.

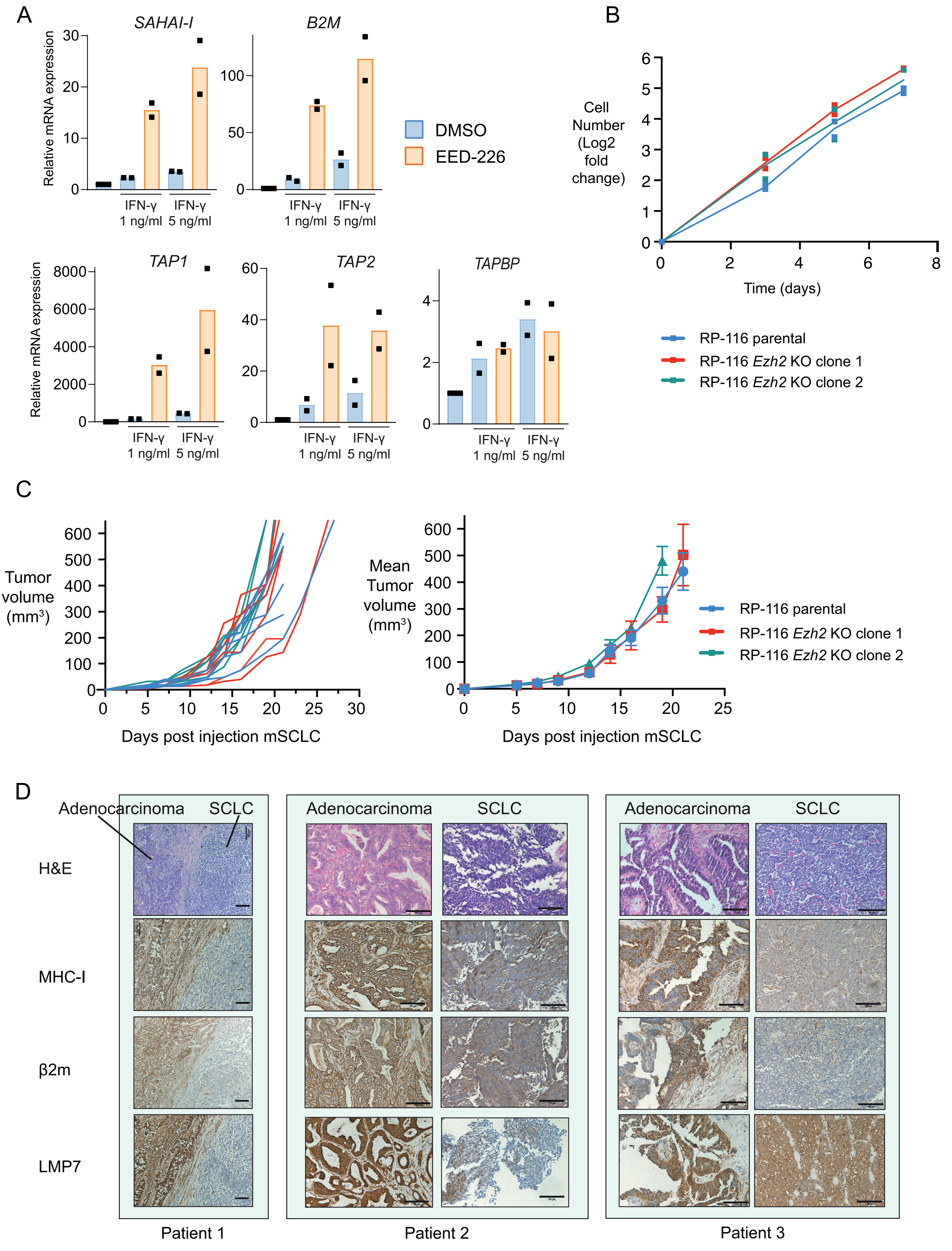

A
SAHAI-I
B2M
TAP1
TAP2
TAPBP
Relative mRNA expression
IFN-γ 1 ng/ml
IFN-γ 5 ng/ml
DMSO
EED-226
B
Cell Number (Log2 fold change)
Time (days)
RP-116 parental
RP-116 Ezh2 KO clone 1
RP-116 Ezh2 KO clone 2
C
Tumor volume (mm3)
Mean Tumor volume (mm3)
Days post injection mSCLC
RP-116 parental
RP-116 Ezh2 KO clone 1
RP-116 Ezh2 KO clone 2
D
Adenocarcinoma
SCLC
H&E
MHC-I
β2m
LMP7
Patient 1
Patient 2
Patient 3

Figure S6. Related to Figure 6. PRC2-mediated silencing of MHC-I antigen presentation promotes immune evasion. (A) qRT-PCR analysis of MHC-I gene expression in DFT1 cells C5065 treated with EED-226 3 μM or DMSO control for 10 days and pulsed with the indicated doses of devil IFN-γ for 6 hr. Bars denote mean fold change in mRNA expression relative to non-interferon treated DMSO control from independent experiments and individual points denote the mean of technical triplicates from each experiment. *SAHAI-1* encodes Tasmanian devil MHC-I. (B) Proliferation of parental RP-116 and RP-116 *Ezh2* knockout clones *in vitro*. Line indicates mean log2 fold change in cell number compared to baseline. Individual points indicate the results of independent experiments. (C) mSCLC tumor growth following subcutaneous transplant of 10^6 parental or *Ezh2* knockout RP-116 cells into NOD-scid IL2Rγ^{null} mice. Endpoint at a tumor volume of 500 mm^3. 6 mice per group. Right panel shows mean ± SEM of tumor growth. *Ezh2* KO clone 1 used for experiment in Figure 6C and 6D. (D) Immunohistochemistry for MHC-I, β2m and LMP7 (immunoproteasome) in patients with transformation of EGFR mutant lung adenocarcinoma to SCLC. Patient 1 was described in Figure 6E, additional staining of post-mortem tumor samples is shown. Clinical information relating to patient 2 and patient 3 is described in the methods. All scale bars are 100 μm.

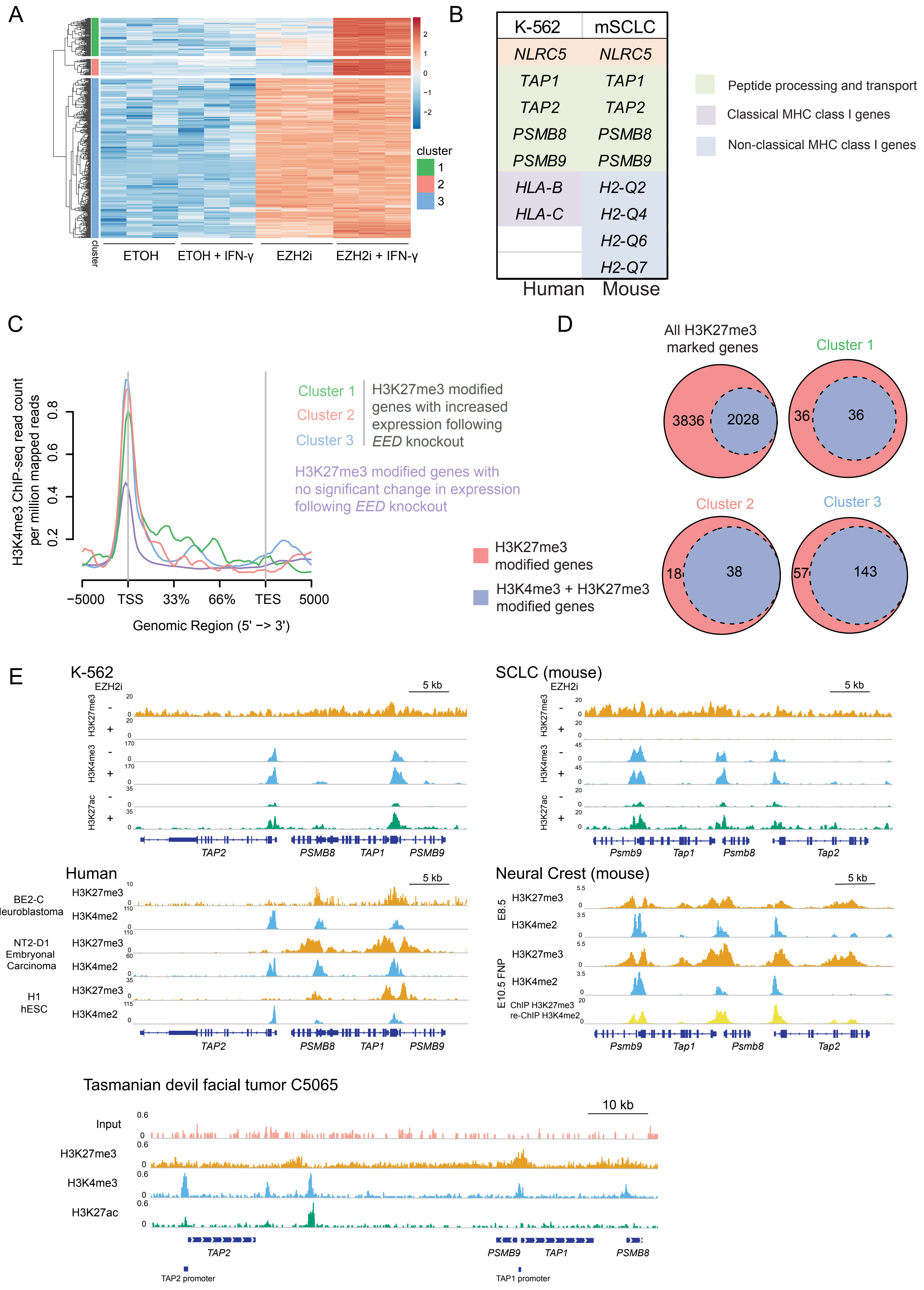

A
cluster
ETOH
ETOH + IFN-γ
EZH2i
EZH2i + IFN-γ
cluster
1
2
3
B
K-562
mSCLC
NLRC5
NLRC5
TAP1
TAP1
TAP2
TAP2
PSMB8
PSMB8
PSMB9
PSMB9
HLA-B
H2-Q2
HLA-C
H2-Q4
H2-Q6
H2-Q7
Human
Mouse
Peptide processing and transport
Classical MHC class I genes
Non-classical MHC class I genes
C
H3K4me3 ChIP-seq read count per million mapped reads
Cluster 1
Cluster 2
Cluster 3
H3K27me3 modified genes with increased expression following EED knockout
H3K27me3 modified genes with no significant change in expression following EED knockout
−5000
TSS
33%
66%
TES
5000
Genomic Region (5' −> 3')
D
All H3K27me3 marked genes
Cluster 1
3836
2028
36
36
Cluster 2
Cluster 3
18
38
57
143
H3K27me3 modified genes
H3K4me3 + H3K27me3 modified genes
E
K-562
EZH2i
5 kb
H3K27me3
H3K4me3
H3K27ac
TAP2
PSMB8
TAP1
PSMB9
SCLC (mouse)
Psmb9
Tap1
Psmb8
Tap2
Human
BE2-C Neuroblastoma
NT2-D1 Embryonal Carcinoma
H1 hESC
H3K4me2
Neural Crest (mouse)
E8.5
E10.5 FNP
ChIP H3K27me3 re-ChIP H3K4me2
Tasmanian devil facial tumor C5065
10 kb
Input
TAP2 promoter
TAP1 promoter

Figure S7. Related to Figure 7. An evolutionarily conserved function of PRC2 maintains bivalency at MHC-I gene promoters in MHC-I low cancers. (A) Heatmap displaying H3K27me3 modified genes upregulated by > 1.5 log2 fold change in mouse SCLC cells (RP-116) treated with EPZ-011989 3 μM for 10 days and pulsed with IFN-γ 10 ng/ml compared to ETOH treated control cells. Genes were clustered using Euclidean clustering, and clusters were separated using a kmeans of 3. Red indicates higher expression and blue indicates lower expression. (B) H3K27me3 modified MHC-I genes identified in clusters 2 and 3 in either K-562 cells or mSCLC cells. (C) H3K4me3 average ChIP-seq read density profiles in H3K27me3 modified genes derepressed following *EED* depletion in K-562 cells (all genes in heatmap in figure 7A, separated by cluster) compared to the H3K4me3 read density profile of H3K27me3 modified genes that are not induced. (D) Venn diagrams show the number H3K27me3 modified genes with concurrent H3K4me3 modification (purple) identified within each cluster of the heatmap in K-562 cells. (E) Bivalent H3K27me3 and H3K4me3 modification of MHC-I gene promoters in human, mouse and Tasmanian devil cells and mouse neural crest cells. K-562, mSCLC (RP-116) and Tasmanian devil ChIP-seq was performed in house. K-562 and mSCLC were treated with EPZ-011989 3 μM or ETOH control for 10 days prior to harvest. Neural crest data from GSE89435 (Minoux et al., 2017), BE2-C (neuroblastoma) from GSE80151 (Zeid et al., 2018), H1-hESC from ENCODE (https://www.encodeproject.org/).

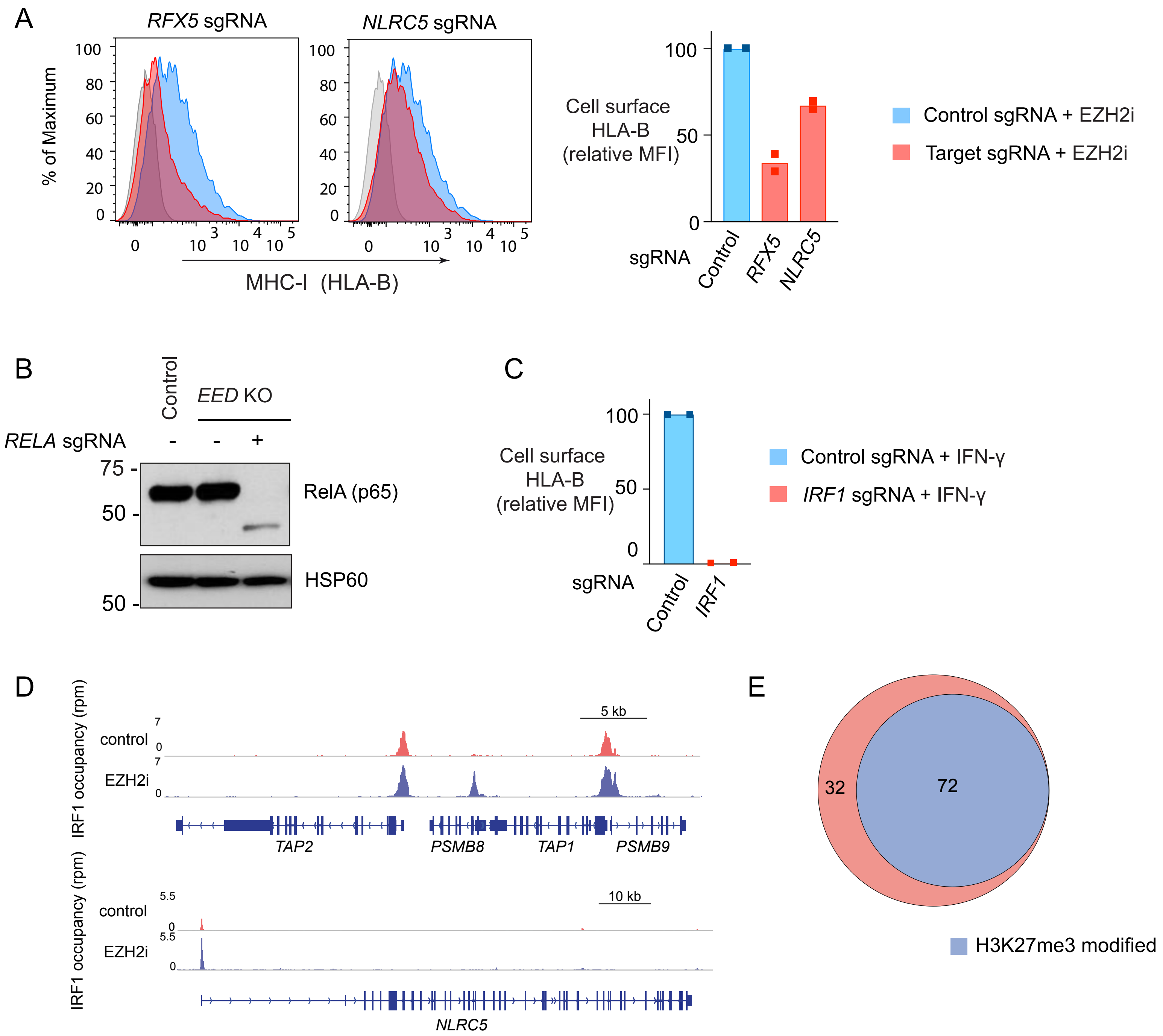
A
RFX5 sgRNA
NLRC5 sgRNA
% of Maximum
MHC-I (HLA-B)
Cell surface HLA-B (relative MFI)
Control sgRNA + EZH2i
Target sgRNA + EZH2i
sgRNA
Control
RFX5
NLRC5
B
Control
EED KO
RELA sgRNA
RelA (p65)
HSP60
C
Cell surface HLA-B (relative MFI)
Control sgRNA + IFN-γ
IRF1 sgRNA + IFN-γ
sgRNA
Control
IRF1
D
IRF1 occupancy (rpm)
control
EZH2i
5 kb
TAP2
PSMB8
TAP1
PSMB9
10 kb
NLRC5
E
32
72
H3K27me3 modified

Figure S8. Related to Figure 8. Loss of PRC2 provides a permissive chromatin environment to facilitate MHC-I expression. (A) Kelly Cas9 cells transduced with either control, *NLRC5*- or *RFX5*-specific sgRNA were treated with EPZ-011989 3 μM for 10 days prior to analysis for cell surface HLA-B. Bars represent mean fold change in HLA-B mean fluorescence intensity (MFI) in *RFX5* or *NLRC5* depleted cells compared to control cells from independent experiments, indicated by individual points. (B) RelA immunoblot in *EED* knockout K-562 Cas9 cells transduced with control or *RELA*-specific sgRNA. (C) Cell surface HLA-B in K-562 Cas9 cells expressing control or *IRF1*-specific sgRNA following incubation with IFN-γ 10 ng/ml for 24 hr. Bars represent mean fold change in HLA-B MFI from independent experiments. (D) IRF1 occupancy at MHC-I APP genes. ChIP-seq using an IRF1-specific antibody in K-562 cells treated with EPZ-011989 3μM or ETOH control for 10 days. (E) Venn diagram contains genes showing increased IRF1 binding (fold increase > 4 at tss) in EZH2i treated compared to control cells (n = 104). The number of these genes that were H3K27me3 modified is indicated in purple (n=72). Overall, IRF1 binding was detected at the tss of 1169 genes, of which 161 were H3K27me3 modified. IRF1 and H3K27me3 ChIP-seq were performed in K-562 cells treated with EPZ-011989 3μM or ETOH control for 10 days.
